# Associations of Vitamin D Receptor (ApaI, FokI, TaqI, BsmI) Polymorphisms with Neurodegenerative Diseases in the Middle East, North Africa and Turkiye (MENA&T) Region: A Systematic Review and Meta-Analysis Toward Population-Specific Precision Medicine

**DOI:** 10.3390/jpm16060277

**Published:** 2026-05-22

**Authors:** Ahmed Abo Kalam, Jameela Roshanuddin, BalaSubramani Gattu Linga, Faisal E. Ibrahim, Rand Hamdan, Thomas Farrell, Zeena Saeed BU Shurbak, Wael M. Y. Mohamed, Nader Al-Dewik

**Affiliations:** 1 Department of Research, Women’s Wellness and Research Center (WWRC), Hamad Medical Corporation (HMC), Doha 3050, Qatar; v-aabokalam@hamad.qa (A.A.K.); juddin1@hamad.qa (J.R.); blinga@hamad.qa (B.G.L.); fibrahim23@hamad.qa (F.E.I.); rand.mahmoud1608@gmail.com (R.H.); tfarrell@hamad.qa (T.F.); 2 Qatar Preterm and Precision Medicine Research Clinic, Women’s Wellness and Research Center (WWRC), Hamad Medical Corporation (HMC), Doha 3050, Qatar; 3 Translational and Precision Medicine Research Lab, Women’s Wellness and Research Center (WWRC), Hamad Medical Corporation (HMC), Doha 3050, Qatar; 4 Translational Research Institute (TRI), Hamad Medical Corporation (HMC), Doha 3050, Qatar; 5 Faculty of Medicine, Qatar University, Doha 2713, Qatar; 6 Department of Obstetrics and Gynecology, Women’s Wellness and Research Center (WWRC), Hamad Medical Corporation (HMC), Doha 3050, Qatar; zshurbak@hamad.qa; 7 Basic Medical Science Department, Kulliyyah of Medicine, International Islamic University Malaysia (IIUM), Kuantan 25200, Malaysia; 8 Newborn Screening Unit, Neonatal Intensive Care Unit (NICU), Department of Pediatrics and Neonatology, Women’s Wellness and Research Center (WWRC), Hamad Medical Corporation (HMC), Doha 3050, Qatar; 9 Genomics and Precision Medicine (GPM), College of Health & Life Science (CHLS), Hamad Bin Khalifa University (HBKU), Doha 34110, Qatar; 10 Faculty of Health and Social Care Sciences, Kingston University, St. George’s University of London, London KT1 2EE, UK

**Keywords:** vitamin D receptor, *VDR* polymorphisms, multiple sclerosis, Parkinson’s disease, Alzheimer’s disease, Middle East, North Africa, and Türkiye (MENA&T), personalized medicine, precision medicine

## Abstract

**Background**: Vitamin D receptor (*VDR*) polymorphisms have been widely investigated as genetic determinants of neurodegenerative diseases, yet findings remain inconsistent and population-dependent. Evidence from the Middle East, North Africa, and Türkiye (MENA&T) regions, which is characterized by widespread vitamin D deficiency and distinct genetic backgrounds, has not been comprehensively synthesized. **Methods**: We conducted a systematic review and meta-analysis evaluating associations between four common *VDR* polymorphisms (ApaI rs7975232, FokI rs2228570, TaqI rs731236, and BsmI rs1544410) and the risk of multiple sclerosis (MS), Parkinson’s disease (PD), and Alzheimer’s disease (AD) in MENA&T populations. Six databases were searched from inception to November 2025. Pooled odds ratios (ORs) with 95% confidence intervals (CIs) were estimated using fixed- and random-effects models across multiple genetic contrasts. Subgroup analyses by ethnicity were conducted for MS. Study quality was assessed using the Newcastle–Ottawa Scale (NOS), and the certainty of evidence was assessed using the Grading of Recommendations Assessment, Development, and Evaluation (GRADE). **Results**: Nineteen unique case–control studies (20 reports), including 4744 participants, were included. For MS, the ApaI polymorphism showed consistent associations with increased risk across genetic models (random-effects ORs = 1.4–1.9), with stronger effects in Arab and Iranian populations and no association in Turkish cohorts. FokI showed associations with MS under selected genetic models, particularly recessive and homozygous contrasts, although findings were not consistent across all analytical approaches. TaqI showed model-dependent associations with substantial heterogeneity, while BsmI showed no significant association. For AD, a meta-analysis of two studies showed no significant associations. For PD, ApaI showed associations with increased risk across several models without heterogeneity; however, these findings were based on a limited number of studies. Overall certainty of evidence ranged from very low to moderate. **Conclusions**: In MENA&T populations, *VDR* ApaI polymorphism shows consistent evidence of association with MS susceptibility, while FokI may be associated under specific genetic models; evidence for AD and PD remains limited and should be considered exploratory. These findings highlight population-specific genetic heterogeneity and underscore the need for further large-scale studies to confirm these associations. These population-specific genetic associations underscore the importance of incorporating *VDR* genotyping into precision medicine frameworks for neurodegenerative disease risk stratification in MENA&T populations, where vitamin D deficiency is highly prevalent.

## 1. Introduction

Neurodegenerative disorders, such as Multiple Sclerosis (MS), Parkinson’s Disease (PD), and Alzheimer’s Disease (AD), represent an increasing global health burden driven by population ageing and marked geographic variation in disease incidence, prevalence, and outcomes [1]. Although these conditions differ in clinical phenotype and underlying pathology, they share multifactorial aetiologies shaped by complex interactions between genetic susceptibility and environmental exposures. Understanding disease risks and associations vary across populations, and identifying modifiable pathways within this context remains a central objective in contemporary neuroepidemiology. Vitamin D has emerged as a promising modifiable risk factor due to its immunomodulatory and neuroprotective properties and the strong influence of geography, lifestyle, and cultural practices on vitamin D status [2,3,4,5].

The human Vitamin D receptor (*VDR*) gene spans approximately 100 kb on chromosome 12q13–14 and contains multiple common single-nucleotide polymorphisms (SNPs) that may alter receptor function or expression and have been extensively investigated in relation to neuroimmune and neurodegenerative disorders [6,7,8,9,10,11]. Among these variants, four have been most extensively studied: FokI (rs2228570), BsmI (rs1544410), ApaI (rs7975232), and TaqI (rs731236) have been most frequently examined in population-based genetic association studies [7,12,13,14,15,16]. These SNPs, located in the 5’ region (FokI) or 3’ region/introns (BsmI, ApaI, TaqI) of the gene, can influence *VDR*’s transcriptional activity or mRNA stability. By modulating *VDR* signaling, such polymorphisms have been proposed as genetic contributors to inter-individual and inter-population differences in susceptibility to immune-mediated and neurodegenerative disorders [14,17].

*VDR* is a ligand-activated transcription factor expressed in various central nervous system (CNS) cell types and brain regions linked to cognition and movement, suggesting its relevance to neurodegeneration. Vitamin D influences the brain both through local metabolic signaling and by regulating genes involved in neuroinflammation, oxidative stress, synaptic function, and neuronal survival. *VDR* signaling may modify neurodegeneration by: (1) limiting neuroinflammation via innate immune modulation; (2) supporting antioxidant defenses and redox balance, which counters oxidative damage seen in MS, PD, and AD; (3) promoting neurotrophic and synaptic health, reducing neuronal vulnerability and aiding repair; and (4) maintaining blood–brain barrier integrity and neuroprotective homeostasis, all mechanisms connected to neurodegenerative conditions [18,19,20].

In MS, an immune-mediated demyelinating disease of the CNS, the vitamin D–*VDR* axis is consistently implicated [21]. Epidemiologic studies demonstrate an inverse relationship between vitamin D status and MS prevalence, and experimental evidence indicates that *VDR* signaling is required for vitamin D’s protective effects in autoimmune demyelination models [21,22,23,24]. Numerous case–control studies have evaluated associations between *VDR* SNPs and MS risk; however, findings have been heterogeneous and often population-specific. For example, a Turkish case–control study reported that higher frequencies of the FokI T allele and the TaqI C allele among MS patients compared with controls, whereas other investigations have reported weaker, null, or inconsistent associations across different populations [17,25].

PD and AD are age-related neurodegenerative disorders for which vitamin D biology has attracted increasing epidemiologic interest [26,27,28,29]. Although observational and prospective studies have linked low circulating vitamin D levels to PD and AD outcomes, these findings remain associational. In PD, lower vitamin D in patients may reflect reduced mobility and limited sunlight exposure rather than a causal effect of vitamin D on disease risk [30]. Similarly, meta-analyses of dementia and AD show an inverse association with vitamin D status but caution that causal inference is limited by residual confounding and the observational nature of the data [31,32,33,34,35,36]. Experimentally, *VDR* activation in animal models can reduce dopaminergic neuron loss and amyloid pathology through anti-inflammatory and antioxidative mechanisms [14,18]. On the other hand, genetic association studies have examined whether *VDR* polymorphisms contribute to PD or AD susceptibility. Meta-analyses suggest that certain *VDR* variants (e.g., BsmI or TaqI) may modestly increase AD susceptibility in some populations. Likewise, recent meta-analytic evidence indicates that the *VDR* polymorphisms (ApaI, BsmI, FokI, and TaqI) have been investigated in relation to PD, with generally modest overall effects. However, substantial ethnic variability has been observed, implying that genetic background, vitamin D exposure, lifestyle factors, and population structure may jointly modulate *VDR*-associated disease susceptibility [14,37].

The Middle East, North Africa, and Türkiye (MENA&T) region provides a biologically and epidemiologically informative setting for examining *VDR* genetics, given the high burden of vitamin D deficiency, the region’s unique population structure, and its underrepresentation in prior genetic syntheses [15]. The MENA region and Türkiye provide a particularly relevant setting for examining *VDR* genetics. Paradoxically, despite high sunlight exposure, vitamin D deficiency is prevalent among MENA populations due to lifestyle and cultural factors [38,39]. This widespread hypovitaminosis D may amplify the effects of functional genetic variation in *VDR*, such that polymorphisms-exert greater influence on dysregulation or neurodegeneration [7,40,41]. In MS, the MENA region has experienced a rising incidence in recent decades, coinciding with urbanization, smoking, and nutritional changes [42]. Within this context, preliminary studies (predominantly in Türkiye and Egypt) have reported associations between *VDR* polymorphisms and MS susceptibility, including significant findings for FokI and TaqI variants in Turkish cohorts and *VDR* haplotypes in Egyptian populations [25,43]. In contrast, data on *VDR* polymorphisms in MENA populations with AD or PD remain scarce.

Vitamin D exerts its effects through the *VDR*, a ligand-activated nuclear transcription factor expressed in immune and CNS cell types. Functional variation in *VDR* may alter transcriptional responses in the context of reduced vitamin D availability [44]. Genetic variants in the *VDR* gene alter receptor function and vitamin D signalling efficiency, thereby influencing susceptibility to neuroinflammatory and neurodegenerative conditions [45].

To date, no systematic synthesis has evaluated the association between *VDR* polymorphisms and neurodegenerative disease risk specifically in the MENA&T populations. Existing meta-analyses have largely focused on European or East Asian cohorts, limiting generalizability to regions with distinct genetic and environmental contexts [14,46]. In accordance with Preferred Reporting Items for Systematic Reviews and Meta-Analyses (PRISMA) guidelines, the present study aims to systematically identify, critically appraise, and quantitatively synthesize available evidence on associations between four key *VDR* polymorphisms (FokI, BsmI, ApaI, and TaqI) and susceptibility to MS, PD, and AD in populations from the Middle East, North Africa, and Türkiye. By pooling effect estimates, assessing heterogeneity, and exploring population-specific patterns, this meta-analysis seeks to clarify the contribution of *VDR* genetic variation to neurodegenerative disease risk in these understudied populations and to inform future translational and precision medicine research in the context of epidemiology and public health.

From a personalized medicine perspective, identifying population-specific genetic risk markers is essential for developing tailored prevention and intervention strategies. *VDR* polymorphisms represent candidate pharmacogenomic markers that may inform individualized vitamin D supplementation strategies and risk-stratified screening protocols for neurodegenerative diseases in genetically distinct populations. This study contributes to the growing evidence base for precision approaches to neurodegenerative disease prevention in underrepresented populations.

## 2. Materials and Methods

### 2.1. Search Strategy and Eligibility Criteria

We systematically searched PubMed, Scopus, EMBASE, the Cochrane Library, Web of Science, and Google Scholar from database inception to 12 November 2025. The search strategy was designed to identify studies examining the association between *VDR* gene polymorphisms and neurodegenerative diseases, using combinations of controlled vocabulary terms and free-text keywords related to vitamin D and its receptor, *VDR* genetic variants (ApaI, TaqI, BsmI, and FokI, including corresponding rs identifiers), genetic association and genotyping, and neurodegenerative conditions including MS, PD, and AD, with geographic terms covering the Middle East, North Africa, and Türkiye. Full search strategies for all databases are provided in Table S1.

The review was conducted in accordance with the PRISMA 2020 guidelines and methodological guidance from the Cochrane Handbook for Systematic Reviews (version 6.5), and was registered prospectively in the International Prospective Register of Systematic Reviews (PROSPERO; CRD420251180010) [47,48,49]. The completed PRISMA 2020 checklist is provided in the Supplementary Materials. Although the original protocol focused on populations from the Middle East and North Africa, Türkiye was included following protocol registration due to its frequent inclusion in Middle Eastern and West Asian genetic epidemiology, documented genetic admixture with neighboring populations, and comparable environmental and vitamin D-related exposure profiles relevant to *VDR* polymorphism research. This modification did not alter eligibility criteria, outcomes, or analytical methods.

Studies were eligible for inclusion if they met the following criteria: (1) observational genetic association studies with case–control designs; (2) conducted among adult or predominantly adult populations, including mixed-age cohorts, with participants diagnosed with MS, PD, or AD according to established clinical or diagnostic criteria; (3) conducted in populations from the Middle East, North Africa, or Türkiye, with cases and controls drawn from the same underlying population and predominantly of MENA&T ancestry; (4) evaluated one or more *VDR* polymorphisms, namely, ApaI/rs7975232, TaqI/rs731236, BsmI/rs1544410, FokI/rs2228570; (5) included a disease-free control group drawn from the same source population; (6) reported genotype or allele frequencies sufficient to calculate odds ratios (ORs), with corresponding confidence intervals (CIs); and (7) provided genetic data of acceptable quality, including clearly defined genotyping methods.

Studies were excluded if they met any of the following criteria: (1) non-human studies, including animal, in vitro, or post-mortem human studies were excluded only if an appropriate case–control design or extractable genotype data were not available; (2) reviews, meta-analyses, case reports, case series, editorials, and commentaries; conference abstracts were excluded unless they reported extractable genotype or allele frequency data sufficient for quantitative synthesis; (3) studies without extractable genotype or allele frequency data; (4) studies lacking an appropriate disease-free comparator group or using diseased, mixed, or undefined controls; (5) studies conducted in non-MENA&**T** populations or mixed populations without separable regional data; or (6) studies evaluating vitamin D levels, supplementation, or genes other than *VDR* without reporting results for the specified polymorphisms.

All retrieved records were imported into EndNote 21 (Clarivate Analytics, Philadelphia, PA, USA), and duplicate studies were removed using Rayyan (Rayyan Systems Inc., Cambridge, MA, USA). Title and abstract screening were conducted independently by Ahmed, Jameela, and Bala, with discrepancies resolved through discussion. Full-text screening was performed by Ahmed and Jameela, with disagreements resolved by consensus. The study selection process is summarized using a PRISMA flow diagram. The references of all included studies were manually reviewed to identify additional eligible articles.

### 2.2. Data Extraction and Quality of Evidence

Data extraction was performed independently by Ahmed and Jameela using a standardized data extraction form. Extracted information included: first author, year of publication, country, ethnicity, study design, disease type, genotyping method, sample size of cases and controls, age, sex distribution, diagnostic criteria, Hardy–Weinberg equilibrium (HWE) status in controls, *VDR* polymorphism, rs identifier, genotype counts, genotype harmonization procedures (including allele orientation, reference allele alignment, and consistent coding of genotypes across studies), as well as the risk-of-bias assessment tool and overall risk-of-bias judgment. Any discrepancies in data extraction were resolved through discussion and consensus between the reviewers. When critical data was missing, incomplete, or unclear, corresponding authors were contacted for clarification; studies were excluded from quantitative synthesis if required data could not be obtained or reliably inferred.

As all included studies were case–control designs, methodological quality was assessed using the Newcastle–Ottawa Scale (NOS) for case–control studies, which evaluates studies across the domains of selection of study groups, comparability of cases and controls, and ascertainment of exposure [50]. Quality assessment was conducted independently by Ahmed and Rand, with disagreements resolved by discussion. Publication bias was assessed using funnel plots and Egger’s regression test. The Grading of Recommendations Assessment, Development, and Evaluation (GRADE) approach was applied to assess the certainty of evidence for each genetic association outcome, considering risk of bias, inconsistency, indirectness, imprecision, and publication bias [51,52].

### 2.3. Genetic Models

To ensure consistency across studies, genetic associations were evaluated using five standard genetic models: allelic (A vs. G), dominant (AA + GA vs. GG), recessive (AA vs. GA + GG), homozygous (AA vs. GG), and heterozygous (GA vs. GG). For harmonization purposes, the G allele was defined as the major allele and the A allele as the minor allele across all analyses, irrespective of the nomenclature used in individual studies. Because genotype reporting varied across publications, all reported genotype labels and allele definitions were systematically mapped within the data extraction sheet to a unified G/A framework, based strictly on each study’s original genotype descriptions. Genotype counts were extracted according to the author’s reported genotype definitions, and allele and model-specific counts were derived only after verification to ensure internal consistency. Throughout the manuscript. This approach ensured that allele coding, genotype mapping, and model construction were applied uniformly across all included studies, minimizing the risk of misclassification or direction-of-effect errors in the pooled analyses. We recognize that applying a uniform G/A allele coding framework across ethnically diverse MENA&T populations involves an inherent assumption that the same allele functions as the ‘risk’ allele across all populations. However, population-specific differences in allele frequencies, linkage disequilibrium structure, and haplotype backgrounds may modify or even reverse the direction of genetic effects. To mitigate potential bias, allele coding was verified against each study’s original genotype descriptions, and all genotype counts were mapped to the unified framework only after confirming internal consistency. The subgroup analyses by ethnicity were designed, in part, to detect population-specific effect modifications that could indicate differential allele effects.

### 2.4. Outcomes

The primary outcome of this systematic review and meta-analysis was the association between *VDR* gene polymorphisms ApaI (rs7975232), TaqI (rs731236), BsmI (rs1544410), and FokI (rs2228570) and the risk of developing MS, PD, or AD among adult populations from the MENA&T. Associations were evaluated across predefined genetic comparison frameworks, including allele-based and genotype-based contrasts, and were synthesized separately for each disease and each polymorphism. When available, outcome estimates were stratified by population characteristics, including country or ethnicity, sex, genotyping method, and HWE status in control groups. Secondary outcomes included assessments related to genotyping quality, consistency and robustness of associations across studies, potential small-study effects, and the overall certainty of evidence supporting each genetic association.

### 2.5. Statistical Analysis

All statistical analyses were performed in R (version 4.5.2) (R Foundation for Statistical Computing, Vienna, Austria) using the meta package (version 8.1-0). Genetic associations were quantified using ORs with corresponding 95% CIs, and statistical significance was defined as a *p*-value < 0.05. Across all analyses, the minor allele (A) was designated as the effect allele in accordance with standard genome-wide association study (GWAS) conventions, and effect estimates were standardized across studies. Meta-analyses were conducted when two or more studies provided comparable data for a given polymorphism, disease, and genetic model. For comparisons reported by a single study, results were summarized using narrative synthesis. Random-effects models (DerSimonian–Laird variance) were applied to all comparisons to account for between-study variability, with fixed-effect models (Mantel–Haenszel) additionally estimated for comparison. Between-study heterogeneity was assessed using the I^2^ statistic, τ^2^ estimates, and Cochran’s Q test, with heterogeneity considered statistically significant at *p*-value < 0.10 for the Q test.

Subgroup analyses were performed only for MS, for which sufficient data were available, and stratified by ethnicity (Arab, Iranian, and Turkish). HWE was assessed in control groups using standard methods, and sensitivity analyses included leave-one-out cross-validation and excluding studies that deviated from HWE to evaluate the robustness of the pooled estimates. Publication bias was assessed using funnel plot inspection and, where appropriate, formal statistical tests for small-study effects. Given multiple genetic comparisons across polymorphisms and inheritance models, results were interpreted cautiously, emphasizing consistency across models, heterogeneity patterns, and sensitivity analyses rather than reliance on nominal statistical significance alone. Results of meta-analyses were presented using forest plots for each genetic model, and the influence of individual studies was illustrated using leave-one-out sensitivity plots. An overdominant genetic model was prespecified in the study protocol but was not evaluated in the final analyses; this deviation did not affect the primary conclusions of the study.

## 3. Results

**Figure 1:**
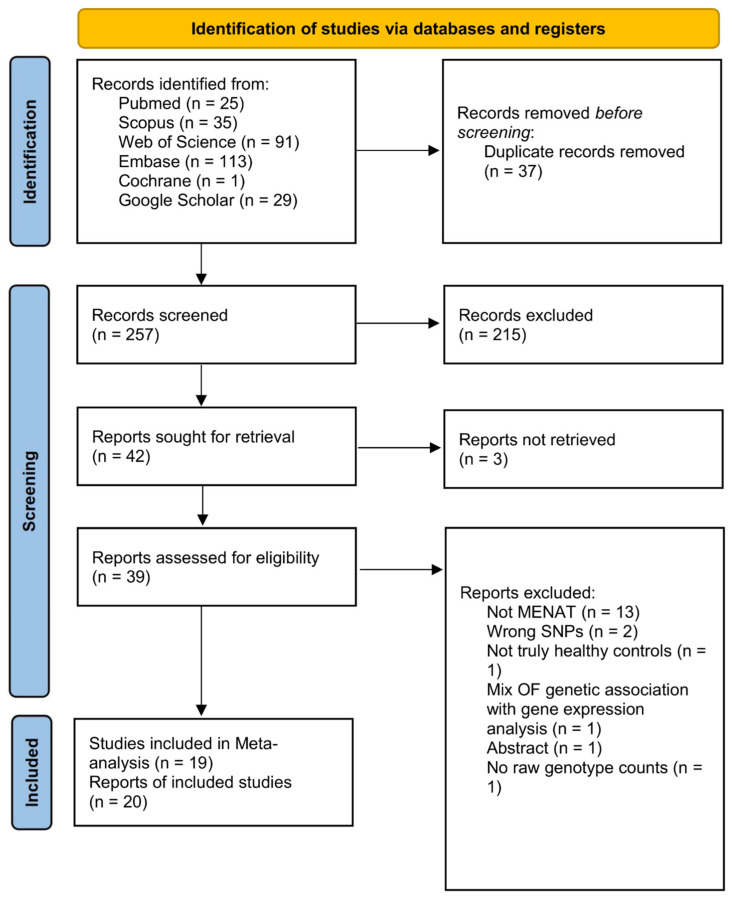
Preferred Reporting Items for Systematic Reviews and Meta-Analyses (PRISMA) flow diagram illustrates the identification of records through database searching, screening of titles and abstracts, eligibility assessment of full-text reports, and final inclusion of studies in systematic review and meta-analysis.

A total of 294 records were identified through database searching. After removal of duplicates and clearly ineligible records, 42 articles were retained for further assessment. Of these, three full-text articles could not be retrieved, leaving 39 for eligibility assessment. Following full-text review, 19 reports were excluded for the following main reasons: studies conducted outside the MENA&T (n = 13), evaluation of non-target SNPs (wrong SNPs; n = 2), use of non-healthy or poorly defined control populations (n = 1), combined genetic association with gene-expression analysis without separable genotype data (n = 1), availability as abstract only (n = 1), and lack of extractable raw genotype counts (n = 1). A total of 20 reports met the eligibility criteria and were included in the review; however, these reports corresponded to 19 unique studies [16,21,25,43,53,54,55,56,57,58,59,60,61,62,63,64,65,66,67], as two reports originated from the same study population [63,68]. The study selection process is illustrated in Figure 1.

The main characteristics of the 19 included studies are summarized in Table 1. All included studies were case–control in design and evaluated associations between *VDR* gene polymorphisms and neurodegenerative diseases. The studies investigated MS, PD, and AD across populations from the MENA&T, with representation of Arab, Iranian, and Turkish populations. The *VDR* polymorphisms assessed included ApaI, TaqI, BsmI, and FokI, with variation in the specific SNPs examined across studies. Sample sizes, diagnostic criteria, genotyping methods, and population characteristics varied between studies and are detailed in Table 1.

Methodological quality assessment of the included studies, evaluated using the NOS [50], is presented in Table 2. All 19 studies were judged to be of high methodological quality, reflecting appropriate case and control selection, adequate comparability between study groups, and reliable exposure ascertainment. No studies were excluded based on quality assessment. The overall certainty of evidence, assessed using the GRADE framework, ranged from low to moderate across outcomes, primarily reflecting the observational design of the included studies, imprecision in some pooled estimates, and inconsistency across studies. Detailed GRADE assessments for each genetic association are provided in Table S2.

### 3.1. ApaI (rs7975232) Polymorphism and Risk of MS

**Figure 2:**
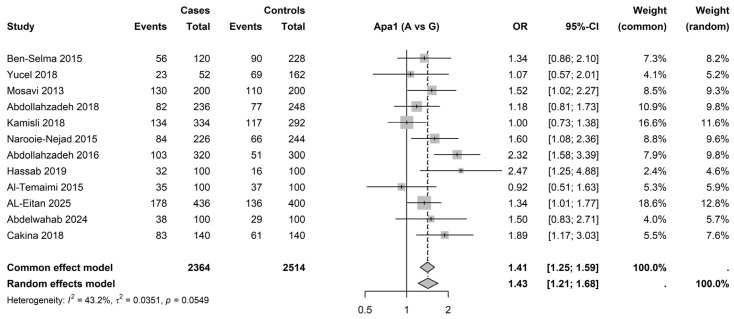
**Forest plot of the association between the vitamin D receptor (*VDR*) ApaI polymorphism (rs7975232) and risk of multiple sclerosis (MS) under the allelic genetic model (A vs. G).** In this analysis, events represent the number of A alleles observed among cases and controls, and odds ratios (ORs) were calculated by comparing A versus G allele frequencies between groups. Study-specific ORs with 95% confidence intervals (CIs) are shown, along with pooled estimates derived from fixed-effect (common-effect) and random-effects models. Between-study heterogeneity was moderate (I^2^ = 43.2%), indicating meaningful variability across studies. Potential sources of heterogeneity, including differences in country and ethnicity, genotyping methods, sample size, and study-specific design characteristics, were explored through predefined sensitivity and subgroup analyses [16,21,43,53,54,56,58,60,62,63,65,67]. Squares represent study-specific odds ratios (ORs), with square size proportional to study weight. Horizontal lines indicate 95% confidence intervals (CIs). Diamonds represent pooled effect estimates for fixed-effect and random-effects models. The solid vertical line indicates the line of no effect (OR = 1), while the dashed vertical line represents the pooled effect estimate.

Twelve studies were included in the meta-analysis assessing the association between the ApaI (rs7975232) polymorphism and susceptibility to MS [16,21,43,53,54,56,58,60,62,63,65,67]. In the allelic model, increased odds of effect (OR = 1.41, 95% CI 1.25–1.59; *p* < 0.0001) and random-effects (OR = 1.43, 95% CI 1.21–1.68; *p* < 0.0001) models (Figure 2 and Figure S1). Similar statistically significant associations were observed in the dominant model (fixed-effect OR = 1.65, 95% CI 1.39–1.95; *p* < 0.0001; random-effects OR = 1.67, 95% CI 1.31–2.14; *p* = 0.0002) (Figure S2), recessive model (fixed-effect OR = 1.43, 95% CI 1.14–1.80; *p* = 0.0023; random-effects OR = 1.41, 95% CI 1.11–1.78; *p* = 0.0044) (Figure S3), homozygous model (fixed-effect OR = 1.94, 95% CI 1.49–2.54; *p* < 0.0001; random-effects OR = 1.91, 95% CI 1.33–2.75; *p* = 0.0005) (Figure S4), and heterozygous model (fixed-effect OR = 1.57, 95% CI 1.31–1.89; *p* < 0.0001; random-effects OR = 1.61, 95% CI 1.25–2.06; *p* = 0.0007) with consistent direction and magnitude of effect across both fixed-effect and random-effects models. (Figure S5) (Table 3).

In ethnicity-stratified analyses, statistically significant associations were observed in Arab and Iranian populations across most genetic models, whereas no statistically significant associations were identified in Turkish populations under any model (Figures S6–S8) (Table 4). Across analyses, heterogeneity was present in several models; however, pooled estimates remained stable in sensitivity analyses, including leave-one-out analyses and the exclusion of studies that deviated from the HWE. Funnel plot inspection and Egger’s regression test did not indicate evidence of publication bias; however, these findings should be interpreted cautiously given the limited number of studies (Table 3; Figures S1–S5). According to the GRADE framework, the certainty of evidence for the ApaI–MS association is moderate.

### 3.2. FokI (rs2228570) Polymorphism and Risk of MS

**Figure 3:**
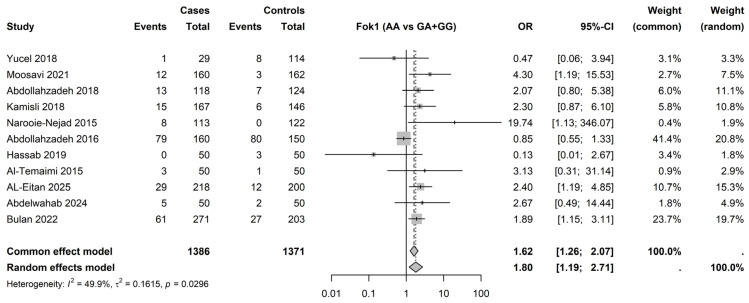
**Forest plot of the association between the vitamin D receptor (*VDR*) FokI polymorphism (rs2228570) and risk of multiple sclerosis (MS) under the recessive genetic model (AA vs. GA + GG).** In this analysis, events represent the number of individuals homozygous for the AA genotype among cases and controls. Study-specific odds ratios (ORs) with 95% confidence intervals (CIs) are shown, together with pooled estimates derived from fixed-effect (common-effect) and random-effects models. Between-study heterogeneity was moderate to substantial (I^2^ = 49.9%), indicating meaningful variability across studies. Potential sources of heterogeneity, including differences in ethnicity, country of origin, sample size, and genotyping methods, were explored through predefined sensitivity and subgroup analyses [16,21,25,43,54,57,58,60,62,65,68]. Squares represent study-specific odds ratios (ORs), with square size proportional to study weight. Horizontal lines indicate 95% confidence intervals (CIs). Diamonds represent pooled effect estimates for fixed-effect and random-effects models. The solid vertical line indicates the line of no effect (OR = 1), while the dashed vertical line represents the pooled effect estimate.

Eleven studies were included in the meta-analysis assessing the association between the FokI (rs2228570) polymorphism and susceptibility to MS [16,25,43,54,57,58,60,62,65,69]. In the overall population, statistically significant associations were observed under several genetic models. In the allelic model (A vs. G), increased odds of MS was observed under the fixed-effect model (OR = 1.35, 95% CI 1.19–1.52; *p* < 0.0001), whereas the association was attenuated and did not reach statistical significance under the random-effects model (OR = 1.28, 95% CI 1.00–1.63; *p* = 0.0521) (Figure 3 and Figure S11). Similarly, the dominant model (AA + AG vs. GG) showed a statistically significant association under the fixed-effect model (OR = 1.36, 95% CI 1.16–1.59; *p* = 0.0002), but not under the random-effects model (OR = 1.27, 95% CI 0.96–1.66; *p* = 0.0916), indicating inconsistency across analytical approaches (Figure S12). In contrast, statistically significant associations were observed under the recessive model (fixed-effect OR = 1.62, 95% CI 1.26–2.07; *p* = 0.0001; random-effects OR = 1.80, 95% CI 1.19–2.71; *p* = 0.0055) and the homozygous model (AA vs. GG) (fixed-effect OR = 2.04, 95% CI 1.52–2.73; *p* < 0.0001; random-effects OR = 2.04, 95% CI 1.37–3.04; *p* = 0.0004) (Figures S13 and S14). The heterozygous model (AG vs. GG) demonstrated a statistically significant association under the fixed-effect model (OR = 1.21, 95% CI 1.02–1.44; *p* = 0.0265), but not under the random-effects model (OR = 1.17, 95% CI 0.91–1.50; *p* = 0.2120), further reflecting model-dependent findings (Figure S15) (Table 3).

In ethnicity-stratified analyses, associations with MS susceptibility varied across populations. Among Arab populations, no consistent associations were observed across most genetic models, whereas statistically significant associations were detected under the recessive and homozygous models. In Iranian populations, statistically significant associations were observed primarily under the dominant and heterozygous models, while evidence for recessive and homozygous effects was less consistent. In Turkish populations, statistically significant associations were identified under the recessive and homozygous models, whereas allelic, dominant, and heterozygous models did not show consistent associations. These findings further support the presence of population-specific effects and model-dependent associations (Figures S16–S18) (Table 4).

Overall, heterogeneity was present in several analyses, indicating variability in effect estimates across studies, but pooled estimates remained stable in sensitivity analyses (Leave-one-out). Funnel plot inspection and Egger’s regression test did not indicate evidence of publication bias; however, these findings should be interpreted cautiously given the borderline number of studies (Table 3; Figures S11–S15). According to the GRADE framework, the certainty of evidence for the association between FokI polymorphism and MS ranged from low to moderate across genetic models, depending on the genetic model evaluated.

### 3.3. TaqI (rs731236) Polymorphism and Risk of MS

**Figure 4:**
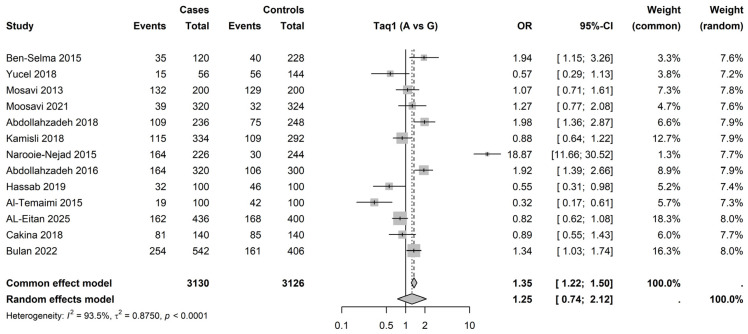
**Forest plot of the association between the vitamin D receptor (*VDR*) TaqI polymorphism (rs731236) and risk of multiple sclerosis (MS) under the allelic genetic model (A vs. G).** In this analysis, events represent the number of A alleles observed among cases and controls, and odds ratios (ORs) were calculated by comparing allele frequencies between groups. Study-specific ORs with 95% confidence intervals (CIs) are shown, together with pooled estimates derived from fixed-effect (common-effect) and random-effects models. Between-study heterogeneity was substantial (I^2^ = 93.5%), indicating considerable variability across studies. Potential sources of heterogeneity, including differences in ethnicity, country of origin, sample size, genotyping methods, and study design, were explored through predefined sensitivity and subgroup analyses [16,21,25,43,53,54,56,57,58,60,62,63,67]. Squares represent study-specific odds ratios (ORs), with square size proportional to study weight. Horizontal lines indicate 95% confidence intervals (CIs). Diamonds represent pooled effect estimates for fixed-effect and random-effects models. The solid vertical line indicates the line of no effect (OR = 1), while the dashed vertical line represents the pooled effect estimate.

Thirteen studies were included in the meta-analysis evaluating the association between the TaqI (rs731236) polymorphism and susceptibility to MS [16,21,25,43,53,54,56,57,58,60,62,63,67]. In the overall population, model-dependent and inconsistent associations were observed. In the allelic model (A vs. G), a statistically significant association was observed under the fixed-effect model (OR = 1.35, 95% CI 1.22–1.50; *p* < 0.0001), whereas no statistically significant association was observed under the random-effects model, indicating inconsistency across analytical approaches (OR = 1.25, 95% CI 0.74–2.12; *p* = 0.399) (Figure 4 and Figure S19).

Similarly, the dominant model (AA + GA vs. GG) showed a statistically significant association under the fixed-effect model (OR = 1.47, 95% CI 1.27–1.71; *p* < 0.0001), but not under the random-effects model (OR = 1.41, 95% CI 0.75–2.65; *p* = 0.2824), further reflecting model-dependent findings (Figure S20). Comparable patterns of inconsistency were observed in the recessive model (fixed-effect OR = 1.43, 95% CI 1.18–1.73; *p* = 0.0002; random-effects OR = 1.24, 95% CI 0.54–2.86; *p* = 0.6074), the homozygous model (AA vs. GG) (fixed-effect OR = 1.73, 95% CI 1.40–2.14; *p* < 0.0001; random-effects OR = 1.42, 95% CI 0.46–4.42; *p* = 0.5433), and the heterozygous model (GA vs. GG) (fixed-effect OR = 1.36, 95% CI 1.16–1.60; *p* = 0.0002; random-effects OR = 1.41, 95% CI 0.82–2.29; *p* = 0.1725) (Figures S21–S23) (Table 3). In ethnicity-stratified analyses, no consistent associations were observed across genetic models within Arab or Turkish populations. In Iranian populations, statistically significant associations were observed under selected models, particularly the dominant and heterozygous models, while other models showed inconsistent and non-robust findings (Figures S24–S26) (Table 4). Overall, these findings suggest a lack of consistent association across populations and genetic models.

Overall, substantial heterogeneity was observed across models; however, sensitivity analyses using leave-one-out approaches and excluding studies that deviated from the HWE did not materially alter the pooled estimates. Funnel plot inspection and Egger’s regression test did not indicate evidence of publication bias; however, these findings should be interpreted cautiously given the borderline number of studies (Table 3; Figures S19–S23). According to the GRADE framework, the certainty of evidence for the association between the TaqI polymorphism and MS ranged from low to moderate across genetic models, depending on the genetic model evaluated. Taken together, the evidence for the TaqI polymorphism appears inconsistent and should be interpreted with caution.

### 3.4. BsmI (rs1544410) Polymorphism and Risk of MS

Ten studies were included in the meta-analysis evaluating the association between the BsmI (rs1544410) polymorphism and susceptibility to MS [16,21,25,43,57,60,62,65,67,68]. In the overall population, no statistically significant associations were observed under any genetic model. In the allelic model (A vs. G), pooled estimates were not significant under either the fixed-effect model (OR = 1.05, 95% CI 0.94–1.18; *p* = 0.3619) or the random-effects model (OR = 1.02, 95% CI 0.81–1.29; *p* = 0.8479) (Figure S28). Similarly, no significant associations were detected in the dominant model (AA + GA vs. GG) (fixed-effect OR = 1.04, 95% CI 0.88–1.23; *p* = 0.6464; random-effects OR = 1.02, 95% CI 0.78–1.33; *p* = 0.8952) (Figure S29), recessive model (AA vs. GA + GG) (fixed-effect OR = 1.13, 95% CI 0.91–1.41; *p* = 0.2607; random-effects OR = 1.18, 95% CI 0.84–1.64; *p* = 0.3345) (Figure S30), homozygous model (AA vs. GG) (fixed-effect OR = 1.16, 95% CI 0.91–1.47; *p* = 0.2337; random-effects OR = 1.14, 95% CI 0.71–1.84; *p* = 0.5855) (Figure S31), or heterozygous model (GA vs. GG) (fixed-effect OR = 1.01, 95% CI 0.84–1.20; *p* = 0.9539; random-effects OR = 1.00, 95% CI 0.80–1.25; *p* = 0.9838) (Figure S32) (Table 3). Significant heterogeneity was observed across most models; however, leave-one-out sensitivity analyses did not materially alter the pooled estimates. In ethnicity-stratified analyses, no significant associations were observed among Arab **or** Turkish populations across genetic models. In Iranian populations, borderline or model-specific associations were observed in selected fixed-effect analyses; however, these findings were not robust under random-effects models and were therefore deemed inconsistent (Figures S33–S35) (Table 4).

Overall, substantial heterogeneity was observed across several analyses, indicating variability in effect estimates across studies, but pooled estimates remained stable in sensitivity analyses. Funnel plot inspection and Egger’s regression test did not indicate evidence of publication bias; however, these findings should be interpreted cautiously given the borderline number of studies (Table 3; Figures S28–S32). According to the GRADE framework, the certainty of evidence for the association between the BsmI polymorphism and MS ranged from low to moderate, depending on the genetic model evaluated.

### 3.5. Vitamin D Receptor Polymorphisms and Risk of PD and AD: Exploratory Analyses

Meta-analyses were performed to evaluate the association between *VDR* polymorphisms and AD based on a limited number of studies (n = 2) [55,61]. For the ApaI (rs7975232) polymorphism, no statistically significant associations with AD susceptibility were observed across genetic models. In the allelic model, pooled estimates were not statistically significant under either the fixed-effect (OR = 1.09, 95% CI 0.84–1.40; *p* = 0.5193) or the random-effects model (OR = 1.10, 95% CI 0.80–1.51; *p* = 0.5541), with no significant heterogeneity. The dominant model showed statistically non-significant results under fixed-effect (OR = 1.32, 95% CI 0.89–1.96; *p* = 0.1691) and random-effects models (OR = 1.30, 95% CI 0.55–3.07; *p* = 0.5490), with significant heterogeneity; sensitivity analysis indicated that omission of a single study altered the pooled estimate (Khorshid et al.). No statistically significant associations were observed in the recessive model (fixed-effect OR = 0.92, 95% CI 0.60–1.40; *p* = 0.6906; random-effects OR = 0.89, 95% CI 0.53–1.49; *p* = 0.6617) or the homozygous model (fixed-effect OR = 0.87, 95% CI 0.50–1.52; *p* = 0.6312; random-effects OR = 0.87, 95% CI 0.50–1.52; *p* = 0.6314), both without significant heterogeneity. The heterozygous model was also statistically non-significant under fixed-effect (OR = 1.41, 95% CI 0.93–2.13; *p* = 0.1029) and random-effects models (OR = 1.37, 95% CI 0.49–3.84; *p* = 0.5500), with significant heterogeneity and sensitivity to the exclusion of one study (Khorshid et al.) (Figure S9). According to the GRADE framework, the certainty of evidence for the ApaI–AD association is very low. These findings should be interpreted with caution given the limited number of studies and observed sensitivity to single-study effects.

For the TaqI (rs731236) polymorphism, no statistically significant associations with AD susceptibility were detected under any genetic model. In the allelic model, pooled estimates were not statistically significant under either the fixed-effect (OR = 1.14, 95% CI 0.88–1.47; *p* = 0.3280) or random-effects model (OR = 1.14, 95% CI 0.88–1.47; *p* = 0.3286), with no significant heterogeneity. Likewise, the dominant model (fixed-effect OR = 1.31, 95% CI 0.92–1.85; *p* = 0.1312; random-effects OR = 1.31, 95% CI 0.90–1.90; *p* = 0.1524), recessive model (fixed-effect OR = 0.93, 95% CI 0.56–1.54; *p* = 0.7861; random-effects OR = 0.93, 95% CI 0.56–1.54; *p* = 0.7864), homozygous model (fixed-effect OR = 1.10, 95% CI 0.65–1.89; *p* = 0.7148; random-effects OR = 1.11, 95% CI 0.65–1.89; *p* = 0.7139), and heterozygous model (fixed-effect OR = 1.38, 95% CI 0.96–2.01; *p* = 0.0860; random-effects OR = 1.39, 95% CI 0.93–2.01; *p* = 0.1123) did not demonstrate significant associations, and no substantial heterogeneity was observed (Figure S27). According to the GRADE framework, the certainty of evidence for the TaqI–AD association is very low. These findings are based on a limited evidence base and should be interpreted cautiously.

Evidence for the association between *VDR* FokI (rs2228570) and BsmI (rs1544410) polymorphisms and AD was limited to a single case–control study conducted in a Turkish population (Dursun et al.) [59]. In this study, no statistically significant associations were observed between AD risk and either FokI or BsmI genotypes or alleles. For FokI, comparisons of genotype and allele frequencies between AD patients and controls yielded ORs close to unity, with non-significant *p*-values (*p* > 0.05). Similarly, for BsmI, neither allelic nor genotypic analyses demonstrated a significant association with AD susceptibility, with ORs not indicating increased or decreased risk and *p*-values exceeding 0.05. Overall, the available evidence for *VDR* polymorphisms in AD is sparse, inconsistent, and should be considered exploratory.

Meta-analysis was conducted to evaluate the association between the *VDR* ApaI (rs7975232) polymorphism and PD based on limited number of studies (n = 2) [59,66]. In the allelic model (A vs. G), a statistically significant association with increased odds of PD was observed under both the fixed-effect model (OR = 1.31, 95% CI 1.05–1.63; *p* = 0.0161) and the random-effects model (OR = 1.31, 95% CI 1.05–1.63; *p* = 0.0161), with no evidence of heterogeneity. Similarly, the dominant model (AA + AG vs. GG) showed a statistically significant association (fixed-effect OR = 1.45, 95% CI 1.07–1.97; *p* = 0.0181; random-effects OR = 1.45, 95% CI 1.07–1.97; *p* = 0.0184), without significant heterogeneity. The recessive model (AA vs. AG + GG) did not demonstrate a statistically significant association under either the fixed-effect (OR = 1.41, 95% CI 0.90–2.22; *p* = 0.1330) or random-effects models (OR = 1.41, 95% CI 0.90–2.22; *p* = 0.1377). In contrast, significant associations were observed in the homozygous model (AA vs. GG) (fixed-effect OR = 1.72, 95% CI 1.06–2.81; *p* = 0.0291; random-effects OR = 1.72, 95% CI 1.06–2.81; *p* = 0.0296) and the heterozygous model (AG vs. GG) (fixed-effect OR = 1.39, 95% CI 1.01–1.92; *p* = 0.0465; random-effects OR = 1.39, 95% CI 1.01–1.92; *p* = 0.0472), with no significant heterogeneity across analyses (Figure S10). However, these findings are based on a limited number of studies and should be interpreted with caution, accordingly these findings should be considered exploratory. According to the GRADE framework, the certainty of evidence for the ApaI–PD association is very low.

Evidence for the association between *VDR* FokI (rs2228570), TaqI (rs731236), and BsmI (rs1544410) polymorphisms and PD was limited to two case–control studies (Alaylıoğlu et al., Turkish population; Fahmy et al., Egyptian population) [59,66]. For FokI, the Turkish study reported no significant association with PD in either allelic (*p* = 0.47) or genotypic (*p* = 0.75) analyses, while the Egyptian study likewise found no significant association for FokI genotypes (*p* = 0.548) or alleles (*p* = 0.51); however, quantitative pooling was not performed because (Fahmy et al.) did not report genotype counts separately for heterozygous and homozygous minor-allele carriers, precluding harmonized meta-analysis. For TaqI, the Turkish study found no significant association with PD risk in genotypic (*p* = 0.24) or allelic (*p* = 0.58) comparisons; TaqI was not assessed in the Egyptian cohort. For BsmI, no significant association with PD was observed in the Turkish study (genotypic *p* = 0.66; allelic *p* = 0.44), and this polymorphism was not evaluated in the Egyptian study. Overall, the available evidence for *VDR* polymorphisms in PD is limited and inconsistent, and should be interpreted as exploratory.

## 4. Discussion

This systematic review and meta-analysis synthesize available evidence on the association between *VDR* gene polymorphisms ApaI, TaqI, BsmI, and FokI, and the risk of MS, PD, and AD in populations from the MENA&T. The findings demonstrate heterogeneous and, in some cases, model-dependent patterns of association across polymorphisms, diseases, and populations, highlighting the importance of population-specific genetic epidemiology in regions characterized by widespread vitamin D deficiency and distinct ancestral backgrounds.

For MS, consistent evidence of associations were observed for the ApaI, while the FokI polymorphisms showed associations under selected genetic models, with effect estimates remaining statistically significant under random-effects assumptions for ApaI, whereas findings for FokI were not consistently replicated across all analytical approaches. These associations were most evident in Arab and Iranian populations, whereas no consistent associations were observed in Turkish subgroups. From an epidemiological perspective, this pattern suggests potential effect modification by population context rather than universal genetic susceptibility. Differences in allele frequencies, linkage disequilibrium structure, consanguinity rates, and unmeasured environmental exposures (particularly vitamin D status) are plausible contributors to the observed heterogeneity. The biological plausibility of these findings is supported by epidemiologic evidence linking vitamin D deficiency to MS risk and by experimental data demonstrating *VDR*-dependent immunomodulatory effects [2,70,71]. In contrast, TaqI exhibited model-dependent and inconsistent associations with substantial heterogeneity, and BsmI showed no evidence of consistent association with MS risk in any model. However, the concentration of heterogeneity within Iranian studies underscores the need for cautious interpretation, as population substructure, small sample size, and residual confounding may influence pooled estimates [72]. It is also important to consider that multiple polymorphisms, genetic models, and subgroup analyses were evaluated, which increases the risk of type I error. Although formal correction methods such as Bonferroni adjustment could be applied, such corrections are overly conservative in meta-analytic settings where comparisons are not fully independent (e.g., genetic models within the same polymorphism are correlated). Greater emphasis was placed on findings that were consistent across genetic models and robust under both fixed-effect and random-effects assumptions. Isolated statistically significant results, particularly those not replicated across models, were interpreted with caution. Furthermore, the assumption of uniform allele coding across ethnically diverse populations is a simplification. Population-specific allele frequency differences—for example, the minor allele in one population may be the major allele in another—could introduce directional bias if the functional allele differs across populations. Although we harmonized allele coding systematically, the absence of individual-level data precluded formal evaluation of population-specific allele effects, and this remains an important limitation.

In the context of AD, meta-analysis based on a limited number of studies (n = 2) revealed no statistically significant associations for ApaI or TaqI polymorphisms, while evidence for FokI and BsmI was limited to a single study precluding robust conclusions. Similarly, for PD, ApaI showed statistically significantly association with increased odds of PD, particularly under dominant and homozygous models, whereas FokI, TaqI, and BsmI did not show consistent associations across the limited available studies. The limited number of studies for AD and PD limits the robustness of these conclusions and underscores the need for further genetic association studies in these populations [14,70]. Accordingly, these findings should be interpreted as exploratory.

Several meta-analyses showed high levels of variation between studies, particularly for TaqI, where I^2^ values were over 80–90% in most genetic models. This heterogeneity likely arises from multiple factors. For example, the studies included participants from various populations across the MENA&T region—such as Arab (Tunisian, Egyptian, Kuwaiti, Jordanian), Iranian, and Turkish groups—which have distinct genetic backgrounds, differences in allele frequencies, and unique patterns of linkage disequilibrium that could influence the association between TaqI and disease in different ways. Second, methodological heterogeneity arises from differences in genotyping platforms (PCR-RFLP vs. TaqMan assays vs. MassARRAY), diagnostic criteria for MS (McDonald 2001 vs. 2010 vs. 2017 criteria, or unspecified), and sample sizes ranging from 50 to 382 cases. Third, unmeasured environmental confounders—particularly vitamin D status, which varies considerably within and between MENA&T countries—may interact with TaqI genotype to produce population-specific effects. Fourth, for TaqI specifically, some studies (e.g., Narooie-Nejad et al.) showed dramatically divergent effect sizes (OR > 10), likely reflecting small sample sizes and stochastic variation. We addressed heterogeneity through random-effects modeling, subgroup analyses by ethnicity, sensitivity analyses (leave-one-out), and exclusion of HWE-deviating studies; however, residual unexplained heterogeneity persists and limits the strength of conclusions for TaqI associations.

Several methodological and contextual factors should be considered when interpreting these results. First, significant between-study heterogeneity was observed in many analyses—particularly in MS analyses dominated by Iranian cohorts—suggesting that population structure, sub-ethnic heterogeneity, and contextual exposures may modify genetic associations. This heterogeneity likely reflects variation in genotyping methods, diagnostic criteria, control selection, and underlying population substructure and admixture [68,73]. Second we acknowledge that the NOS was originally developed for observational epidemiological studies and does not capture several biases specific to genetic association studies. These include population stratification (systematic differences in ancestry between cases and controls), genotyping error or call rate issues, deviations from HWE in controls (which may signal genotyping errors or population substructure), and the appropriateness of the assumed genetic model. More specialized tools such as the Q-Genie (Quality of Genetic Association Studies) instrument or the Venice criteria for credibility assessment of genetic associations would provide more granular quality evaluation for genetic studies [74,75]. We considered these limitations when interpreting the uniformly high NOS scores and emphasize that high NOS ratings should not be equated with absence of genetic-study-specific biases. The certainty of evidence assessed via GRADE ranged from very low to moderate. This reflects the inherent limitations of observational genetic association studies, including imprecision in pooled estimates and small sample sizes, particularly for PD and AD, where the number of available studies was limited [52]. Consequently, statistical power to detect modest genetic effects outside MS was restricted. Third, the meta-analytic evidence base was heavily weighted toward MS, with relatively few studies available for PD and AD, limiting cross-disease comparability and the generalizability and exploratory nature of findings beyond MS. Fourth, while publication bias was formally assessed where feasible, the possibility of residual publication bias cannot be excluded, especially for outcomes with fewer studies and small sample sizes. Fifth, the analysis focused on single *VDR* polymorphisms and did not account for gene–gene interactions, polygenic risk, or haplotype structure, which may better capture the complex genetic architecture underlying neurodegenerative diseases. On the other hand, unmeasured vitamin D status represents a particularly important limitation of this meta-analysis. Vitamin D deficiency is highly prevalent in MENA&T populations (estimated 50–90% prevalence depending on country and subpopulation [38,39,76]), and circulating 25(OH)D levels may act as both a confounder and an effect modifier of *VDR* genetic associations. As a confounder, low vitamin D status is independently associated with increased neurodegenerative disease risk, and if also correlated with *VDR* genotype (through gene–environment correlation), it could inflate or deflate observed genetic associations. As an effect modifier, *VDR* polymorphisms that alter receptor function or expression may exert stronger phenotypic effects under conditions of vitamin D insufficiency, where the functional consequence of reduced *VDR* efficiency is amplified, compared to vitamin D-replete states where sufficient ligand availability may partially compensate for reduced receptor function. None of the included studies incorporated serum 25(OH)D levels as a covariate in their genetic analyses, and only two studies (Moosavi et al., 2021 [57]; Fahmy et al., 2021 [66]) reported vitamin D levels descriptively. This represents a critical gap, as the high prevalence of vitamin D deficiency in the MENA&T region may systematically amplify *VDR*-disease associations compared to populations with higher vitamin D status, limiting the generalizability of our findings. Future studies should incorporate formal gene–environment interaction analyses evaluating *VDR* genotype x vitamin D status interactions to disentangle genetic from environmental contributions to disease risk. Finally, multiple genetic models and polymorphisms were evaluated, introducing a multiple-testing burden; although results were interpreted cautiously with emphasis on consistency across models and sensitivity analyses, false-positive associations cannot be entirely ruled out. Lastly, environmental factors such as sun exposure, dietary vitamin D intake, and supplementation were rarely accounted for, potentially confounding genetic associations [76]. This limitation is particularly relevant given robust evidence linking vitamin D deficiency itself to MS risk, suggesting that unmeasured vitamin D status may confound or modify observed genetic associations [71].

Our findings have important implications for future research in genetic epidemiology and population health. First, they underscore the need for larger, well-powered genetic-association studies in MENA&T populations, particularly for PD and AD, where available data remain sparse [77]. Second, future investigations should incorporate direct measures of vitamin D status, evaluate gene–environment interactions, and integrate functional analyses to better characterize the biological relevance of *VDR* variants and to distinguish genetic susceptibility from environmental and lifestyle influences. Third, population-specific genetic architectures warrant caution when extrapolating findings from other regions, given the unique admixture patterns and the persistent underrepresentation of these populations in global genomic reference datasets. Together, these findings emphasize the importance of regionally focused genetic epidemiology to inform hypothesis generation and guide the design of future mechanistic and longitudinal studies, rather than immediate clinical translation [78].

The population-specific patterns of *VDR*-disease associations identified in this study have direct implications for personalized medicine approaches in the MENA&T region. The differential associations observed between Arab, Iranian, and Turkish subgroups suggest that a ‘one-size-fits-all’ approach to genetic risk assessment is insufficient. *VDR* genotyping, combined with vitamin D status measurement, could enable clinicians to identify individuals at higher genetic risk of neurodegenerative diseases within specific MENA&T populations, facilitating targeted screening and tailored preventive interventions such as population-appropriate vitamin D supplementation regimens. Furthermore, the observed interaction between *VDR* genetic variants and the high regional prevalence of vitamin D deficiency highlights the potential for gene–environment-informed clinical decision-making. Future clinical studies should evaluate whether *VDR* genotype-guided vitamin D supplementation can modify neurodegenerative disease risk in these populations, a critical step toward implementing precision prevention strategies.

While the present meta-analysis is observational and does not directly evaluate therapeutic interventions, the identified *VDR*-disease associations may have several potential clinical applications. First, if validated in prospective studies, *VDR* genotyping could be integrated into risk stratification algorithms for MS, enabling earlier identification of at-risk individuals in MENA&T populations where vitamin D deficiency is endemic. Second, the population-specific nature of these associations (e.g., stronger effects in Arab and Iranian versus Turkish cohorts) suggests that pharmacogenomic approaches to vitamin D supplementation—adjusting dose, timing, or formulation based on *VDR* genotype—may warrant investigation. Experimental evidence demonstrating that *VDR* activation reduces dopaminergic neuron loss and amyloid pathology (in animal models [14,18,31,32]) supports the biological plausibility of *VDR*-targeted therapeutic strategies. Third, given that vitamin D supplementation trials in MS have shown mixed results [4,5], stratification by *VDR* genotype in future clinical trials could help identify subpopulations most likely to benefit from supplementation, thereby improving trial design and clinical outcomes. However, we emphasize that these potential applications remain speculative at this stage and require validation through well-designed prospective cohort studies, Mendelian randomization analyses, and genotype-stratified randomized controlled trials before clinical implementation.

## 5. Conclusions

In this systematic review and meta-analysis of MENA&T populations, common vitamin D receptor polymorphisms demonstrated disease- and population-specific associations with neurodegenerative disorders. The most consistent evidence was observed for associations between the *VDR* ApaI and MS susceptibility, while the FokI variant showed associations under specific genetic models, particularly in Arab and Iranian populations, whereas no corresponding associations were identified in Turkish cohorts. In contrast, evidence for AD was limited and largely null, while findings for PD were based on a small number of studies and modest effect sizes. Accordingly, these findings should be considered exploratory. These results should be viewed as associational rather than causal, given the observational nature of the underlying data, limited statistical power for PD and AD, and the absence of direct measures of vitamin D status or formal gene–environment interaction analyses. These results should not be considered confirmatory and require validation in larger independent cohorts. The overall certainty of evidence ranged from low to moderate, reflecting heterogeneity across studies, small sample sizes for non-MS outcomes, and imprecision in pooled estimates, despite generally good methodological quality as assessed by the NOS. Notwithstanding these limitations, this study provides the first comprehensive synthesis of *VDR* genetic associations with neurodegenerative diseases in MENA&T populations, highlighting substantial population heterogeneity and the limited transferability of genetic risk estimates derived from predominantly European or East Asian cohorts. Future research should prioritize larger, well-powered studies, harmonized genotyping and reporting standards, and integration of environmental and biochemical measures, particularly vitamin D status, to better characterize gene–environment interplay within the vitamin D pathway. Such efforts are essential for refining etiologic understanding and supporting population-specific approaches in neurodegenerative disease epidemiology. These findings contribute to the foundation for future precision medicine approaches in MENA&T populations by identifying population-specific *VDR* genetic risk markers that may, in the future, inform targeted screening, risk stratification, and individualized prevention strategies for neurodegenerative diseases.

## Figures and Tables

**Table 1:** Characteristics of the studies included in the systematic review and meta-analysis, including study design, population, disease type, *VDR* polymorphisms assessed, sample size, and genotyping methods.

Study (Author, Year)	Country	Ethnicity	Study Design	Disease	Cases (n)	Controls (n)	Mean Age (Cases/Controls)	Sex (M/F) Cases/Controls	SNPs Investigated	Diagnostic Criteria	Genotyping Method
Ben-Selma et al., 2015 [53]	Tunisia	Arab	Case–control	Multiple sclerosis	60	114	Not reported explicitly. Men: 43.31 years Women: 35.81 years/Men: 37 years Women: 33 years	22/38; 47/67	TaqI, ApaI	MS diagnosis based on MRI, CSF & evoked potentials; formal criteria not specified	PCR-RFLP
Yucel et al., 2018 [54]	Turkey	Turkish	Case–control	Multiple sclerosis	29	120	33.7 ± 10.7 years/33.1 ± 8.5 years	8/21; 59/61	TaqI, ApaI, FokI	MS diagnosis: criteria not specified	TaqMan SNP Assay
Gezen-Ak et al., 2007 [55]	Turkey	Turkish	Case–control	Alzheimer’s disease	104	109	75.1 ± 5.7 years/73.6 ± 7.3 years	Not reported	ApaI, TaqI	DSM-IV criteria for late-onset AD	PCR-RFLP
Mosavi et al., 2013 [56]	Iran	Iranian	Case–control	Multiple sclerosis	100	100	40 ± 9 years/40 ± 7 years	41/59; 40/60	ApaI, TaqI	RRMS diagnosed using McDonald criteria (MRI, CSF, evoked potentials)	PCR-RFLP
Moosavi et al., 2021 [57]	Iran	Iranian	Case–control	Multiple sclerosis	160	162	34.3 ± 8.3 years/35.4 ± 7.9 years	45/115; 54/108	FokI, BsmI, TaqI	MS was diagnosed according to the McDonald criteria	PCR-RFLP
Abdollahzadeh et al., 2018 [62]	Iran	Iranian	Case–control	Multiple sclerosis	118	124	37.8 ± 2.5/38.2 ± 3.6	36/82; 39/85	FokI, TaqI, ApaI, BsmI,	Relapsing–remitting MS diagnosed according to McDonald criteria	PCR-RFLP
Kamisli et al., 2018 [58]	Turkey	Turkish	Case–control	Multiple sclerosis	167	146	39.96 ± 9.46/33.81 ± 7.12	46/121; 58/88	TaqI, FokI, ApaI	Multiple sclerosis was diagnosed according to the McDonald criteria	TaqMan SNP Assay
Alaylıoğlu et al., 2017 [59]	Turkey	Turkish	Case–control	Parkinson’s disease	382	242	61.7 ± 11.4/64.6 ± 10.3	221/161; 118/124	TaqI, ApaI, BsmI, FokI,	Parkinson’s disease was diagnosed according to the UK Parkinson’s Disease Society Brain Bank criteria	PCR-RFLP + LightSNiP
Narooie-Nejad et al., 2015 [63]	Iran	Iranian	Case–control	Multiple sclerosis	113	122	32.4 ± 8.9/30.8 ± 10.2	25/88; 28/94	TaqI, ApaI	Multiple sclerosis was diagnosed according to the McDonald criteria	PCR-RFLP
Abdollahzadeh et al., 2016 [60]	Iran	Iranian	Case–control	Multiple sclerosis	160	150	35.9 ± 2.3/36.8 ± 1.8	40/120; 38/112	ApaI, BsmI, FokI, TaqI	Relapsing–remitting MS diagnosed according to McDonald criteria	PCR-RFLP
Hassab et al., 2019 [43]	Egypt	Arab	Case–control	Multiple sclerosis	50	50	30 ± 8 years/32 ± 11 years	22/28; 18/32	FokI, BsmI, ApaI, TaqI	Multiple sclerosis was diagnosed according to the McDonald criteria	PCR–RFLP
Al-Temaimi et al., 2015 [21]	Kuwait	Arab	Case–control	Multiple sclerosis	50	50	33.44 ± 9.63 years/28.68 ± 7.98 years	17/33; 19/31	TaqI, BsmI, ApaI, FokI	Multiple sclerosis (RRMS/SPMS) diagnosed in a specialist MS clinic; formal criteria not explicitly stated	TaqMan SNP Assay
AL-Eitan et al., 2025 [16]	Jordan	Arab	Case–control	Multiple sclerosis	218	200	35.70 ± 10.20 years/27.08 ± 6.19 years	71/147; 62/138	FokI, BsmI, TaqI, ApaI	Multiple sclerosis was diagnosed according to the McDonald criteria	MassARRAY
Khorshid et al., 2013 [61]	Iran	Iranian	Case–control	Alzheimer’s disease	145	162	78.55 ± 7.80 years/77.14 ± 6.95 years	63/91; 63/99	TaqI, ApaI	Late-onset Alzheimer’s disease was diagnosed according to DSM-IV criteria	PCR–RFLP
Dursun et al., 2013 [64]	Turkey	Turkish	Case–control	Alzheimer’s disease	108	115	74 ± 4.2 years/75.2 ± 6.8 years	Not reported	BsmI, FokI	Late-onset Alzheimer’s disease was diagnosed according to DSM-IV criteria	PCR–RFLP
Abdelwahab et al., 2024 [65]	Egypt	Arab	Case–control	Multiple sclerosis	50	50	Median age cases: 30 years (IQR 23.75–36.0); controls: age-matched (18–40 years)	9/41; 15/35	FokI, ApaI, BsmI	Relapsing–remitting MS diagnosed according to McDonald criteria (2017)	TaqMan SNP Assay
Fahmy et al., 2021 [66]	Egypt	Arab	Case–control	Parkinson disease	50	50	60.8 ± 5.0 years/61.4 ± 5.7 years	34/16; 32/18	FokI, Apal	Idiopathic Parkinson’s disease diagnosed according to MDS clinical diagnostic criteria (2015)	TaqMan PCR
Cakina et al., 2018 [67]	Turkey	Turkish	Case–control	Multiple sclerosis	70	70	44.4 ± 11.2 years/38.2 ± 9.5 years	19/51; 22/48	ApaI, BsmI, TaqI	Multiple sclerosis was diagnosed according to the Revised McDonald Criteria	PCR–RFLP
Bulan et al., 2022 [25]	Turkey	Turkish	Case–control	Multiple sclerosis	271	203	Not reported	Not reported	FokI, BsmI, TaqI	Multiple sclerosis was diagnosed according to the McDonald criteria	PCR–RFLP

**Table 2:** Quality assessment of included studies using the Newcastle–Ottawa Scale (NOS). All studies were rated high quality across selection, comparability, and exposure domains. “✩ = one point awarded in the Newcastle–Ottawa Scale (NOS). NOS scores of 7–9 stars indicate high quality, 4–6 stars indicate moderate quality, and 0–3 stars indicate low quality.”

Study (Author, Year)	Selection	Comparability	Exposure	Total	Number of Stars	Quality Judgement
Ben-Selma et al., 2015 [53]	✩✩✩✩	✩	✩✩	✩✩✩✩✩✩✩	7	High
Yucel et al., 2018 [54]	✩✩✩✩	✩	✩✩	✩✩✩✩✩✩✩	7	High
Gezen-Ak et al., 2007 [55]	✩✩✩✩	✩	✩✩	✩✩✩✩✩✩✩	7	High
Mosavi et al., 2013 [56]	✩✩✩✩	✩✩	✩✩	✩✩✩✩✩✩✩✩	8	High
Moosavi et al., 2021 [57]	✩✩✩✩	✩✩	✩✩	✩✩✩✩✩✩✩✩	8	High
Abdollahzadeh et al., 2018 [62]	✩✩✩✩	✩	✩✩	✩✩✩✩✩✩✩	7	High
Kamisli et al., 2018 [58]	✩✩✩✩	✩	✩✩	✩✩✩✩✩✩✩	7	High
Alaylıoğlu et al., 2017 [59]	✩✩✩✩	✩	✩✩	✩✩✩✩✩✩✩	7	High
Narooie-Nejad et al., 2015 [63]	✩✩✩✩	✩	✩✩	✩✩✩✩✩✩✩	7	High
Abdollahzadeh et al., 2016 [60]	✩✩✩✩	✩	✩✩	✩✩✩✩✩✩✩	7	High
Hassab et al., 2019 [43]	✩✩✩✩	✩✩	✩✩	✩✩✩✩✩✩✩✩	8	High
Al-Temaimi et al., 2015 [21]	✩✩✩✩	✩✩	✩✩	✩✩✩✩✩✩✩✩	8	High
AL-Eitan et al., 2025 [16]	✩✩✩✩	✩✩	✩✩	✩✩✩✩✩✩✩✩	8	High
Khorshid et al., 2013 [61]	✩✩✩✩	✩	✩✩	✩✩✩✩✩✩✩	7	High
Dursun et al., 2013 [64]	✩✩✩✩	✩	✩✩	✩✩✩✩✩✩✩	7	High
Abdelwahab et al., 2024 [65]	✩✩✩✩	✩✩	✩✩	✩✩✩✩✩✩✩✩	8	High
Fahmy et al., 2021 [66]	✩✩✩✩	✩	✩✩	✩✩✩✩✩✩✩	7	High
Cakina et al., 2018 [67]	✩✩✩✩	✩	✩✩	✩✩✩✩✩✩✩	7	High
Bulan et al., 2022 [25]	✩✩✩✩	✩	✩✩	✩✩✩✩✩✩✩	7	High

**Table 3:** Summary of the associations between *VDR* polymorphisms (FokI, BsmI, ApaI, and TaqI) and risk of MS.

Polymorphism	Genetic Model	OR (95% CI)	*p*-Value	Heterogeneity (I^2^)	*p*-Value (Egger’s Test)
FokI	Allelic	1.28 (1.00–1.63)	0.0521	66.0%	0.2230
	Dominant	1.27 (0.96–1.66)	0.0916	56.5%	0.0759
	Recessive	1.80 (1.19–2.71)	0.0055	49.9%	0.3772
	Homozygous	2.04 (1.37–3.04)	0.0004	40.6%	0.9629
	Heterozygous	1.17 (0.91–1.50)	0.2120	45.7%	0.1480
BsmI	Allelic	1.02 (0.81–1.29)	0.8479	71.1%	0.517
	Dominant	1.02 (0.78–1.33)	0.8952	57.4%	0.6129
	Recessive	1.18 (0.84–1.64)	0.3345	53.3%	0.3340
	Homozygous	1.14 (0.71–1.84)	0.5855	65.0%	0.3543
	Heterozygous	1.00 (0.80–1.25)	0.9838	27.6%	0.6335
ApaI	Allelic	1.43 (1.21–1.68)	<0.0001	43.2%	0.5648
	Dominant	1.67 (1.31–2.14)	<0.0001	52.0%	0.0817
	Recessive	1.41 (1.11–1.78)	0.0044	11.8%	0.9214
	Homozygous	1.91 (1.33–2.75)	0.0005	41.9%	0.2527
	Heterozygous	1.61 (1.25–2.06)	0.0002	48.5%	0.0329
TaqI	Allelic	1.25 (0.74–2.12)	0.3990	93.5%	0.9145
	Dominant	1.41 (0.75–2.65)	0.2824	89.3%	0.755
	Recessive	1.24 (0.54–2.86)	0.6074	81.2%	0.9610
	Homozygous	1.42 (0.46–4.42)	0.5433	86.7%	0.9972
	Heterozygous	1.41 (0.86–2.29)	0.1725	82.1%	0.58

**Abbreviations:** *VDR*, vitamin D receptor; MS, multiple sclerosis; OR, odds ratio; CI, confidence interval; I^2^, percentage of between-study heterogeneity. publication bias was evaluated using Egger’s test (*p*-value). **Notes:** Values are pooled ORs with 95% CIs derived from SNP-stratified meta-analyses. Genetic models include allelic, dominant, recessive, homozygous, and heterozygous comparisons.

**Table 4:** Association between *VDR* gene polymorphisms and MS stratified by ethnicity and genetic model.

Ethnicity	Polymorphism	Genetic Model	OR (95% CI)	*p*-Value	I^2^ (%)
Arabs	FokI	Allelic	1.02 [0.61–1.72]	0.9427	68.2
		Dominant	0.95 [0.57–1.58]	0.8325	55.4
		Recessive	2.20 [1.19–4.05]	0.0114	15.7
		Homozygous	2.23 [1.19–4.16]	0.0123	30.5
		Heterozygous	0.91 [0.63–1.31]	0.6037	19.9
	BsmI	Allelic	0.84 [0.62–1.13]	0.2426	41.8
		Dominant	0.82 [0.60–1.11]	0.2030	0
		Recessive	0.76 [0.27–2.13]	0.5953	55.6
		Homozygous	0.71 [0.26–1.96]	0.5107	52.4
		Heterozygous	0.85 [0.61–1.18]	0.3243	0
	ApaI	Allelic	1.37 [1.12–1.67]	0.0019	17.9
		Dominant	1.63 [1.17–2.27]	0.0039	27.5
		Recessive	1.29 [0.80–2.09]	0.2931	17.5
		Homozygous	1.65 [1.07–2.53]	0.0232	0
		Heterozygous	1.68 [1.10–2.56]	0.0157	42.7
	TaqI	Allelic	0.74 [0.36–1.52]	0.4149	85.3
		Dominant	0.75 [0.33–1.73]	0.5017	82.3
		Recessive	0.60 [1.18–2.03]	0.4142	61.9
		Homozygous	0.55 [0.09–3.15]	0.4982	70
		Heterozygous	0.86 [0.42–1.75]	0.6751	74.2
Iranian	FokI	Allelic	1.47 [1.01–2.15]	0.0458	72.6
		Dominant	1.60 [1.21–2.12]	0.0011	25.4
		Recessive	2.22 [0.80–6.17]	0.1266	72.6
		Homozygous	2.63 [0.86–7.99]	0.0887	68.2
		Heterozygous	1.43 [1.07–1.90]	0.0163	2.7
	BsmI	Allelic	1.15 [0.72–1.82]	0.5550	84.6
		Dominant	1.16 [0.65–2.07]	0.6056	81.4
		Recessive	1.27 [0.64–2.55]	0.4956	66.2
		Homozygous	1.30 [0.48–3.54]	0.6044	78.8
		Heterozygous	1.11 [0.69–1.79]	0.6788	70.7
	ApaI	Allelic	1.61 [1.22–2.12]	0.0008	50.9
		Dominant	2.04 [1.16–3.60]	0.0134	70.6
		Recessive	1.86 [1.07–3.23]	0.0277	39.2
		Homozygous	3.87 [1.54–9.69]	0.0039	58.9
		Heterozygous	1.83 [1.09–3.07]	0.0229	68.2
	TaqI	Allelic	2.49 [0.91–6.83]	0.0766	95.8
		Dominant	3.46 [1.07–11.14]	0.0378	92.2
		Recessive	3.33 [0.70–15.95]	0.1315	87.7
		Homozygous	6.31 [0.86–46.04]	0.0693	88.7
		Heterozygous	2.87 [1.21–6.78]	0.0165	85.4
Turkish	FokI	Allelic	1.26 [0.69–2.29]	0.4462	72.6
		Dominant	1.27 [0.63–2.57]	0.5103	75
		Recessive	1.86 [1.21–2.86]	0.0050	0
		Homozygous	2.00 [1.27–3.15]	0.0029	31.6
		Heterozygous	1.19 [0.61–2.31]	0.6098	73.4
	BsmI	Allelic	1.11 [0.88–1.39]	0.3800	0
		Dominant	1.07 [0.77–1.50]	0.6894	0
		Recessive	1.26 [0.83–1.91]	0.2765	0
		Homozygous	1.26 [0.79–2.01]	0.3231	0
		Heterozygous	1.00 [0.70–1.44]	0.9787	0
	ApaI	Allelic	1.25 [0.83–1.89]	0.2838	58.6
		Dominant	1.40 [0.76–2.58]	0.2748	57
		Recessive	1.20 [0.79–1.83]	0.3892	0
		Homozygous	1.45 [0.73–2.85]	0.2871	41.9
		Heterozygous	1.35 [0.76–2.40]	0.3031	41.6
	TaqI	Allelic	0.96 [0.69–1.32]	0.7790	62.4
		Dominant	1.02 [0.78–1.34]	0.8771	0
		Recessive	1.00 [0.53–1.87]	0.9927	68.5
		Homozygous	0.82 [0.36–1.89]	0.6409	68.3
		Heterozygous	0.99 [0.74–1.32]	0.9471	0

**Abbreviations:** *VDR*, vitamin D receptor; MS, multiple sclerosis; OR, odds ratio; CI, confidence interval; I^2^, percentage of between-study heterogeneity. **Notes:** Values are pooled ORs with 95% CIs derived from ethnicity-stratified meta-analyses. Genetic models include allelic, dominant, recessive, homozygous, and heterozygous comparisons.

## Data Availability

This is a research article, and all data generated or analyzed during this study are included in this published article [and its Supplementary Information Files]. All inquiries should be directed to the corresponding authors. Email: naldewik@hamad.qa; nader.al-dewik@kingston.ac.uk; Email: waelmohamed@iium.edu.my; wmy107@gmail.com.

## Supplementary Materials

The following supporting information can be downloaded at: https://www.mdpi.com/article/10.3390/jpm16060277/s1.

## Abbreviations

The following abbreviations are used in this manuscript:

AD	Alzheimer’s Disease
ALS	Amyotrophic Lateral Sclerosis
CI	Confidence Interval
CNS	Central Nervous System
EAE	Experimental Autoimmune Encephalomyelitis
GRADE	Grading of Recommendations Assessment, Development, and Evaluation
GWAS	Genome-Wide Association Study
HWE	Hardy–Weinberg Equilibrium
MENA&T	Middle East, North Africa, and Türkiye
MS	Multiple Sclerosis
NOS	Newcastle-Ottawa Scale
OR	Odds Ratio
PD	Parkinson’s Disease
PRISMA	Preferred Reporting Items for Systematic Reviews and Meta-Analyses
PROSPERO	International Prospective Register of Systematic Reviews
SNP	Single-Nucleotide Polymorphism
*VDR*	Vitamin D Receptor
