# Supplementary material for: Associations of Vitamin D Receptor (ApaI, FokI, TaqI, BsmI) Polymorphisms with Neurodegenerative Diseases in the Middle East, North Africa and Turkiye (MENA&T) Region: A Systematic Review and Meta-Analysis Toward Population-Specific Precision Medicine
Source: J Pers Med. 2026 May 22;16(6):277. doi: 10.3390/jpm16060277 (PMC13301335; doi:10.3390/jpm16060277)
Supplement: Supplementary file 1 [file jpm-16-00277-s001.zip › jpm-4210655-supplementary.pdf]

Supplementary Material

Table S1. Full search strategies for each database and date of inception were stratified for each database

Data Base	Date of Inception	Search Term	Result
Pubmed	12/11/2025	(("Vitamin D"[Mesh] OR "Cholecalciferol"[Mesh] OR "Calcitriol"[Mesh] OR "Vitamin D receptor"[Mesh] OR "Vitamin D" OR "Calcitriol" OR "VDR" OR "Vitamin D receptor" OR "VDR polymorphism" OR "Vitamin D receptor polymorphism" OR "VDR gene" OR "VDR variant" OR "ApaI" OR "TaqI" OR "BsmI" OR "FokI" OR "rs7975232" OR "rs731236" OR "rs1544410" OR "rs2228570") AND ("Neurodegenerative Diseases"[Mesh] OR "Multiple Sclerosis"[Mesh] OR "Parkinson Disease"[Mesh] OR "Alzheimer Disease"[Mesh] OR "multiple sclerosis" OR "Parkinson" OR "Alzheimer" OR "neurodegeneration" OR "neuroinflammation") AND ("Polymorphism, Genetic"[Mesh] OR "Genotype"[Mesh] OR "Alleles"[Mesh] OR "genotyping" OR "gene-environment interaction" OR "association study") AND ("Middle East"[Mesh] OR "Africa, Northern"[Mesh] OR "Arab populations" OR "Persian" OR "Berber" OR "Qatar"[MESH] OR "United Arab Emirates"[MESH] OR "Saudi Arabia"[MESH] OR "Bahrain"[MESH] OR "Kuwait"[MESH] OR "Oman"[MESH] OR "Yeman"[MESH] OR "Iraq"[MESH] OR "Syria"[MESH] OR "Lebanon"[MESH] OR "Jordan"[MESH] OR "Palestine" OR "Occupied Palestine" OR "Egypt"[MESH] OR "Sudan"[MESH] OR "Libya"[MESH] OR "Tunisia"[MESH] OR "Algeria"[MESH] OR "Morocco"[MESH] OR "Mauritania"[MESH] OR "Somalia"[MESH] OR "Djibouti"[MESH] OR "Comoros"[MESH] OR "Iran"[MESH] OR "Turkey"[Mesh]))	25
Web of Science	12/11/2025	(("Vitamin D" OR "Cholecalciferol" OR "Calcitriol" OR "Vitamin D receptor" OR "Vitamin D" OR "Calcitriol" OR "VDR" OR "Vitamin D receptor" OR "VDR polymorphism" OR "Vitamin D receptor polymorphism" OR "VDR gene" OR "VDR variant" OR "ApaI" OR "TaqI" OR "BsmI" OR "FokI" OR "rs7975232" OR "rs731236" OR "rs1544410" OR "rs2228570") AND ("Neurodegenerative Diseases" OR "Multiple Sclerosis" OR "Parkinson Disease" OR "Alzheimer Disease" OR "multiple sclerosis" OR "Parkinson" OR "Alzheimer" OR "neurodegeneration" OR "neuroinflammation") AND ("Polymorphism,	91

		Genetic" OR "Genotype" OR "Alleles" OR "genotyping" OR "gene-environment interaction" OR "association study") AND ("Middle East" OR "Africa, Northern" OR “Turkey”))	
Scopus	12/11/2025	(("Vitamin D" OR "Cholecalciferol" OR "Calcitriol" OR "Vitamin D receptor" OR "Vitamin D" OR "Calcitriol" OR "VDR" OR "Vitamin D receptor" OR "VDR polymorphism" OR "Vitamin D receptor polymorphism" OR "VDR gene" OR "VDR variant" OR "ApaI" OR "TaqI" OR "BsmI" OR "FokI" OR "rs7975232" OR "rs731236" OR "rs1544410" OR "rs2228570") AND ("Neurodegenerative Diseases" OR "Multiple Sclerosis" OR "Parkinson Disease" OR "Alzheimer Disease" OR "multiple sclerosis" OR "Parkinson" OR "Alzheimer" OR "neurodegeneration" OR "neuroinflammation") AND ("Polymorphism, Genetic" OR "Genotype" OR "Alleles" OR "genotyping" OR "gene-environment interaction" OR "association study") AND ("Middle East" OR "Africa, Northern" OR "Turkey" OR "Arab populations" OR "Persian" OR "Berber" OR "Qatar" OR "United Arab Emirates" OR "Saudi Arabia" OR "Bahrain" OR "Kuwait" OR "Oman" OR "Yeman" OR "Iraq" OR "Syria" OR "Lebanon" OR "Jordan" OR "Palestine" OR "Occupied Palestine" OR "Egypt" OR "Sudan" OR "Libya" OR "Tunisia" OR "Algeria" OR "Morocco" OR "Mauritania" OR "Somalia" OR "Djibouti" OR "Comoros" OR "Iran"))	35
Google Scholar	12/11/2025	allintitle: Vitamin D receptors OR vitmain D receptors ApaI OR TaqI OR BsmI OR FokI OR rs7975232 OR rs731236 OR rs1544410 OR rs2228570 OR Parkinson OR Disease OR Alzheimer OR Disease OR multiple OR sclerosis OR Persian OR Berber OR Qatar OR United OR Arab OR Emirates OR Saudi OR Arabia OR Bahrain OR Kuwait OR Oman OR Yeman OR Iraq OR Syria OR Lebanon OR Jordan OR Palestine OR Occupied OR Palestine OR Egypt OR Sudan OR Libya OR Tunisia OR Algeria OR Morocco OR Mauritania OR Somalia OR Djibouti OR Comoros OR Iran OR Turkey.	29
Embase	12/11/2025	(('vitamin d'/exp OR 'vitamin d' OR 'colecalciferol'/exp OR 'colecalciferol' OR 'vitamin d receptor'/exp OR 'vitamin d receptor' OR 'apal gene' OR 'taqi' OR 'bsml gene' OR 'fokl gene' OR 'rs7975232' OR 'rs731236' OR 'rs1544410' OR 'rs2228570') AND ('multiple sclerosis' OR 'parkinson disease' OR 'alzheimer disease' OR 'genetic polymorphism' OR 'allele') AND ('middle east' OR 'north african' OR 'turkey (republic)'))	113

Cochrane	12/11/2025	(("Vitamin D"[Mesh] OR "Cholecalciferol"[Mesh] OR "Calcitriol"[Mesh] OR "Vitamin D receptor"[Mesh] OR "Vitamin D" OR "Calcitriol" OR "VDR" OR "Vitamin D receptor" OR "VDR polymorphism" OR "Vitamin D receptor polymorphism" OR "VDR gene" OR "VDR variant" OR "ApaI" OR "TaqI" OR "BsmI" OR "FokI" OR "rs7975232" OR "rs731236" OR "rs1544410" OR "rs2228570") AND ("Neurodegenerative Diseases"[Mesh] OR "Multiple Sclerosis"[Mesh] OR "Parkinson Disease"[Mesh] OR "Alzheimer Disease"[Mesh] OR "multiple sclerosis" OR "Parkinson" OR "Alzheimer" OR "neurodegeneration" OR "neuroinflammation") AND ("Polymorphism, Genetic"[Mesh] OR "Genotype"[Mesh] OR "Alleles"[Mesh] OR "genotyping" OR "gene-environment interaction" OR "association study") AND ("Middle East"[Mesh] OR "Africa, Northern"[Mesh] OR “Arab populations” OR “Persian” OR “Berber” OR “Qatar”[MESH] OR “United Arab Emirates”[MESH] OR “Saudi Arabia”[MESH] OR “Bahrain”[MESH] OR “Kuwait”[MESH] OR “Oman”[MESH] OR “Yeman”[MESH] OR “Iraq”[MESH] OR “Syria”[MESH] OR “Lebanon”[MESH] OR “Jordan”[MESH] OR “Palestine” OR “Occupied Palestine” OR “Egypt”[MESH] OR “Sudan”[MESH] OR “Libya”[MESH] OR “Tunisia”[MESH] OR “Algeria”[MESH] OR “Morocco”[MESH] OR “Mauritania”[MESH] OR “Somalia”[MESH] OR “Djibouti”[MESH] OR “Comoros”[MESH] OR “Iran”[MESH] OR "Turkey"[Mesh]))	1

Table S2. GRADE certainty of evidence for association between VDR polymorphisms and neurodegenerative diseases (overall MENA&T populations)

Study design: Case–control studies; **Effect measure:** Odds ratio (OR); **Initial certainty:** Low (observational studies)

Disease	SNP	Genetic model	No. of studies	Risk of bias	Inconsistency	Indirectness	Imprecision	Publication bias	Overall certainty
MS	ApaI	Allelic	12	Not serious	Not serious	Not serious	Not serious	Not detected	Moderate
		Dominant	12	Not serious	Not serious	Not serious	Not serious	Not detected	Moderate
		Recessive	12	Not serious	Not serious	Not serious	Not serious	Not detected	Moderate
		Homozygous	12	Not serious	Not serious	Not serious	Not serious	Not detected	Moderate
		Heterozygous	12	Not serious	Not serious	Not serious	Not serious	Not detected	Moderate
MS	FokI	Allelic	11	Not serious	Serious	Not serious	Not serious	Not detected	Low
		Dominant	11	Not serious	Serious	Not serious	Not serious	Not detected	Low
		Recessive	11	Not serious	Serious	Not serious	Not serious	Not detected	Low–Moderate
		Homozygous	11	Not serious	Serious	Not serious	Not serious	Not detected	Low–Moderate
		Heterozygous	11	Not serious	Serious	Not serious	Not serious	Not detected	Low
MS	TaqI	All models	13	Not serious	Serious	Not serious	Serious	Not detected	Low
MS	BsmI	All models	10	Not serious	Not serious	Not serious	Serious	Not detected	Low
PD	ApaI	Allelic	2	Not serious	Not serious	Not serious	Serious	Not assessed	Low

		Dominant	2	Not serious	Not serious	Not serious	Serious	Not assessed	Low
		Recessive	2	Not serious	Not serious	Not serious	Very serious	Not assessed	Very low
		Homozygous	2	Not serious	Not serious	Not serious	Serious	Not assessed	Low
		Heterozygous	2	Not serious	Not serious	Not serious	Serious	Not assessed	Low
PD	FokI	All models	1	Not serious	Not applicable	Not serious	Very serious	Not assessed	Very low
PD	TaqI	All models	1	Not serious	Not applicable	Not serious	Very serious	Not assessed	Very low
PD	BsmI	All models	1	Not serious	Not applicable	Not serious	Very serious	Not assessed	Very low
AD	ApaI	All models	2	Not serious	Serious	Not serious	Very serious	Not assessed	Very low
AD	TaqI	All models	2	Not serious	Not serious	Not serious	Very serious	Not assessed	Very low
AD	FokI	All models	1	Not serious	Not applicable	Not serious	Very serious	Not assessed	Very low
AD	BsmI	All models	1	Not serious	Not applicable	Not serious	Very serious	Not assessed	Very low
AD	FokI	All models	1	Not serious	Not applicable	Not serious	Very serious	Not assessed	Very low
AD	BsmI	All models	1	Not serious	Not applicable	Not serious	Very serious	Not assessed	Very low

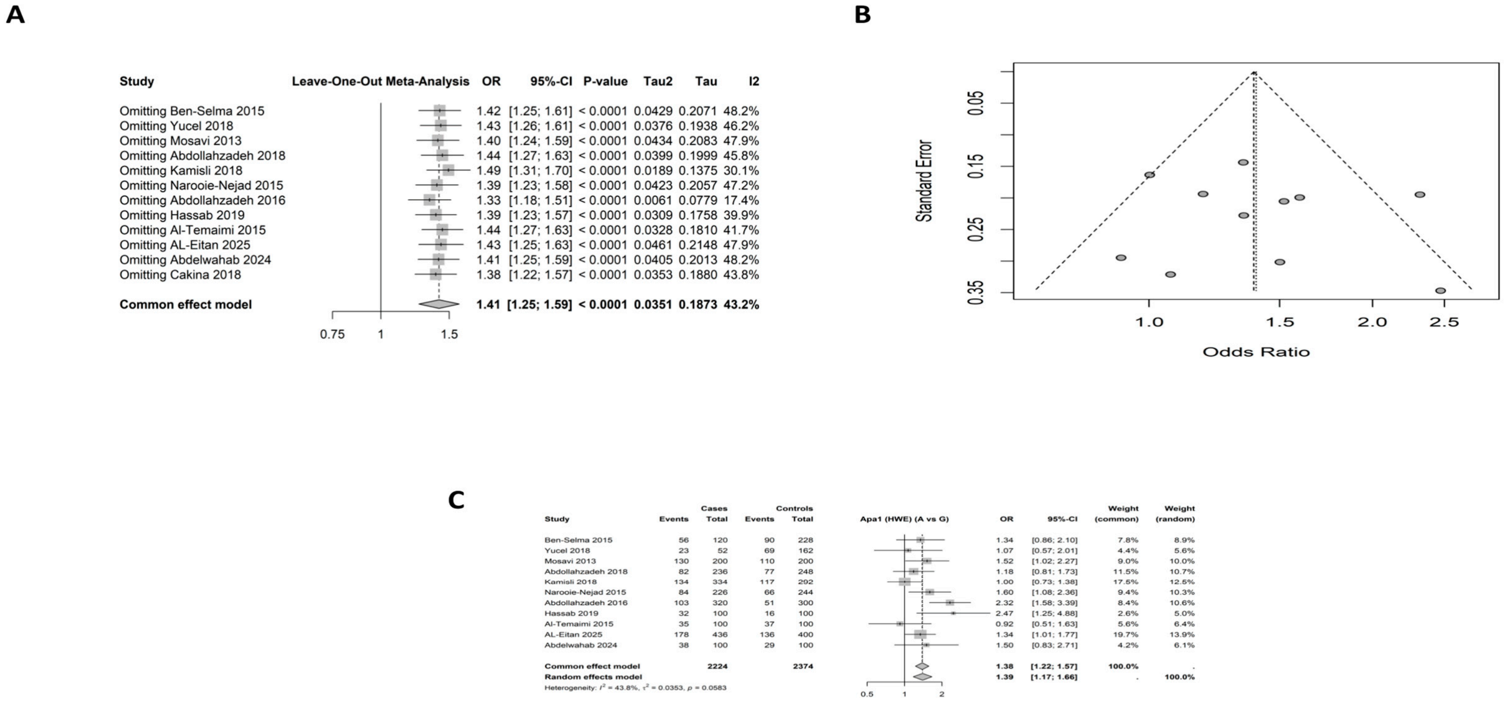

Figure S1. VDR ApaI allelic model analyses and multiple sclerosis risk in the overall population. These analyses support the main meta-analysis of the association between the vitamin D receptor (VDR) ApaI polymorphism and multiple sclerosis (MS) risk under the allelic model (A vs G) in the overall population. **(A)** Leave-one-out sensitivity analysis evaluating the influence of each individual study on the pooled effect estimate. **(B)** Funnel plot assessing small-study effects and potential publication bias. **(C)** Forest plot restricted to studies in which genotype distributions in control groups conformed to Hardy–Weinberg equilibrium (HWE). Pooled estimates were calculated using common-effect and random-effects models, with between-study heterogeneity assessed using Cochran's Q test and quantified by the I^2 statistic. The primary forest plot, including all eligible studies, is presented in the main text **[1-12]**

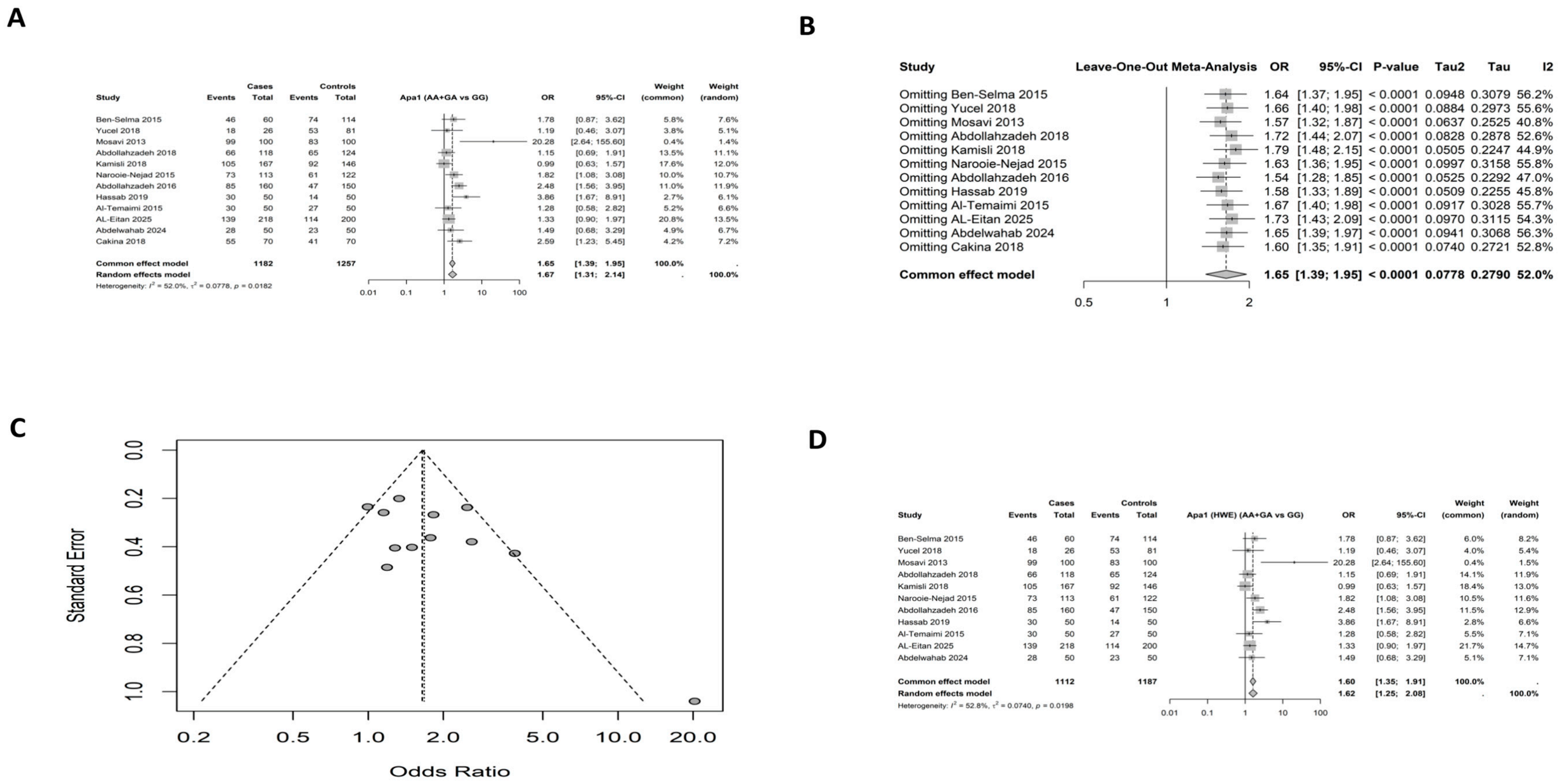

Figure S2. VDR ApaI dominant model analyses and multiple sclerosis risk in the overall population. These analyses support the main meta-analysis of the association between the vitamin D receptor (VDR) ApaI polymorphism and multiple sclerosis (MS) risk under the dominant genetic model (AA+AG vs GG). **(A)** Forest plot of individual studies and pooled estimates. **(B)** Leave-one-out sensitivity analysis. **(C)** Funnel plot assessing potential publication bias. **(D)** Forest plot restricted to studies that maintained the Hardy–Weinberg equilibrium in control groups. Pooled odds ratios (ORs) and 95% confidence intervals (CIs) were estimated using common-effect and random-effects models, with heterogeneity assessed using Cochran's Q test and the I^2 statistic**[1-12]**

A

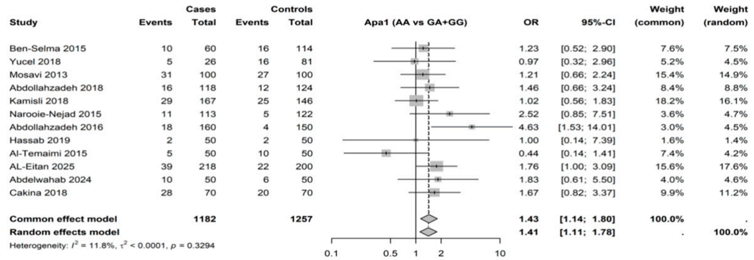

B

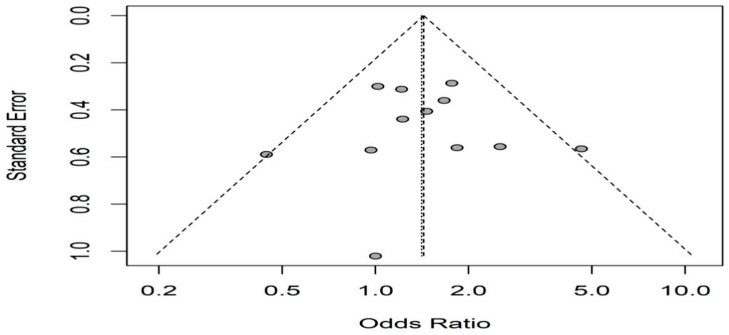

Figure S3. VDR ApaI recessive model analyses and multiple sclerosis risk in the overall population. These analyses support the main meta-analysis of the association between the vitamin D receptor (VDR) ApaI polymorphism and multiple sclerosis (MS) risk under the recessive genetic model (AA vs AG+GG). **(A)** Forest plot of individual studies and pooled estimates. **(B)** Funnel plot assessing potential publication bias. Pooled odds ratios (ORs) and 95% confidence intervals (CIs) were estimated using common-effect and random-effects models, with between-study heterogeneity evaluated using Cochran's Q test and the I^2 statistic **[1-12]**

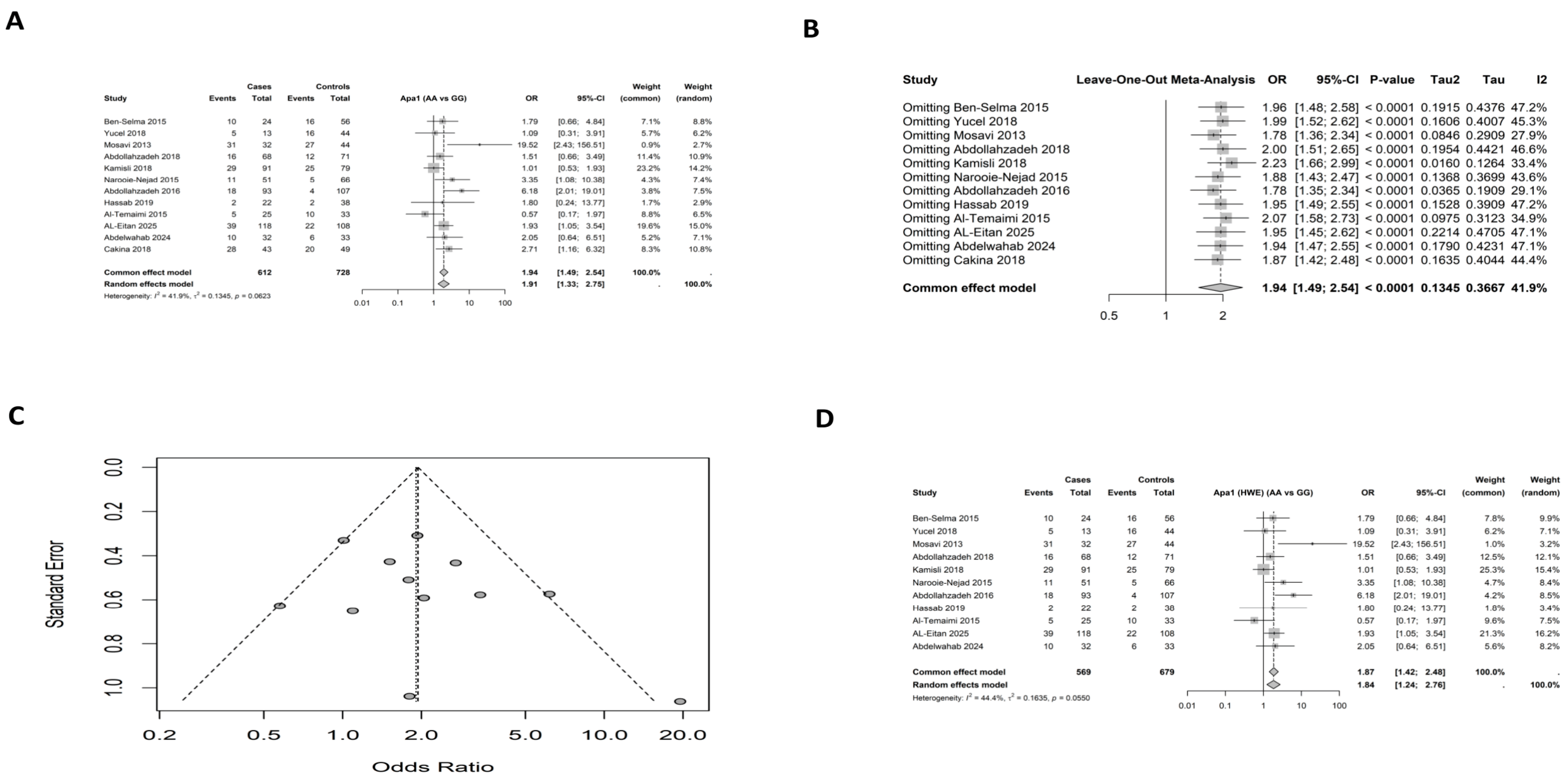

Figure S4. VDR ApaI homozygous model analyses and multiple sclerosis risk in the overall population. These analyses support the main meta-analysis of the association between the vitamin D receptor (VDR) ApaI polymorphism and multiple sclerosis (MS) risk under the homozygous genetic model (AA vs GG). **(A)** Forest plot of individual studies and pooled estimates. **(B)** Leave-one-out sensitivity analysis. **(C)** Funnel plot assessing potential publication bias. **(D)** Forest plot restricted to studies that maintained Hardy–Weinberg equilibrium in control groups. Pooled odds ratios (ORs) and 95% confidence intervals (CIs) were estimated using common-effect and random-effects models, with between-study heterogeneity evaluated using Cochran's Q test and the I^2 statistic **[1-12]**

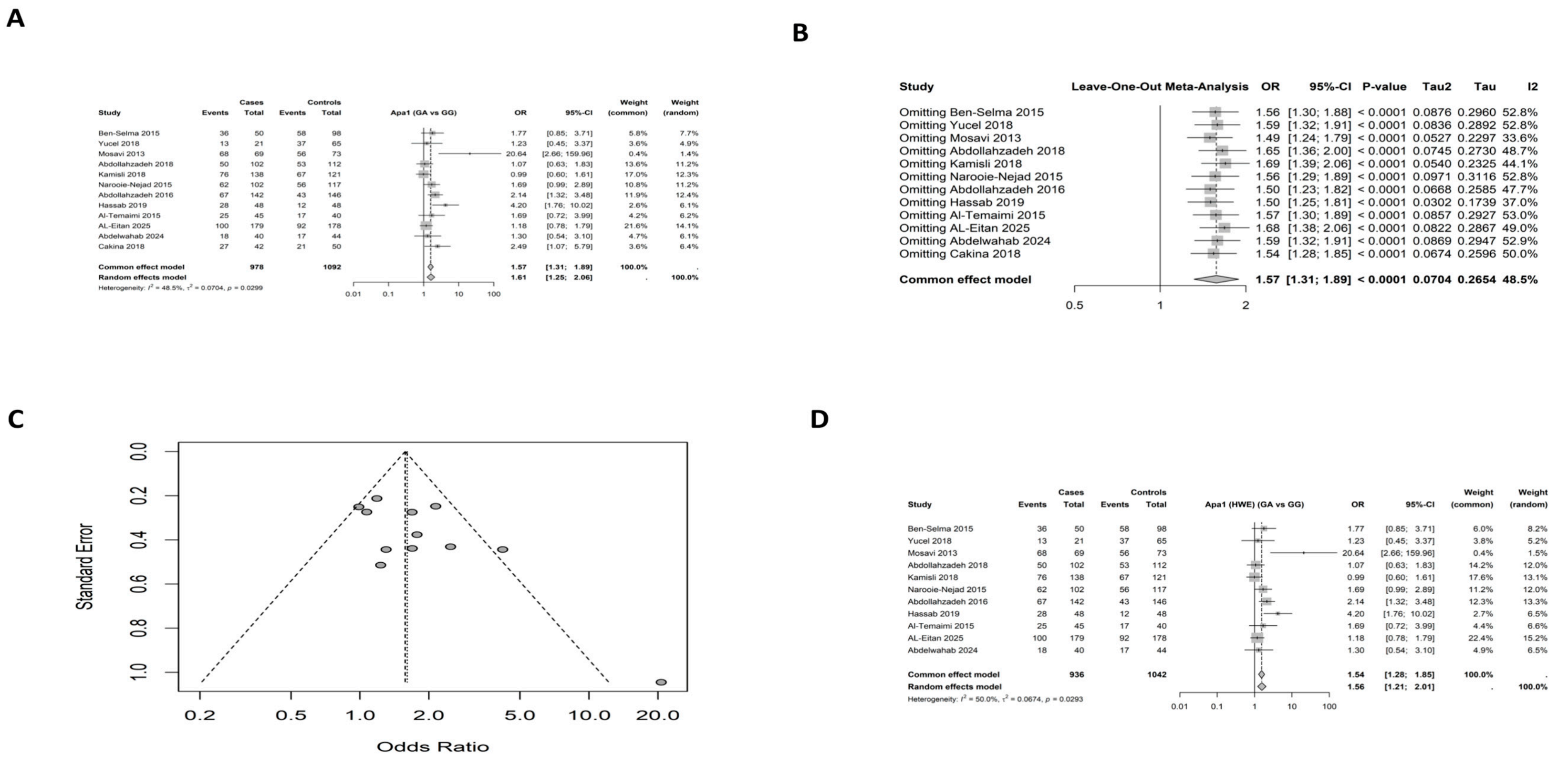

Figure S5. VDR ApaI heterozygous model analyses and multiple sclerosis risk in the overall population. These analyses support the main meta-analysis of the association between the vitamin D receptor (VDR) ApaI polymorphism and multiple sclerosis (MS) risk under the heterozygous genetic model (AG vs GG). **(A)** Forest plot of individual studies and pooled estimates. **(B)** Leave-one-out sensitivity analysis. **(C)** Funnel plot assessing potential publication bias. **(D)** Forest plot restricted to studies that maintained Hardy–Weinberg equilibrium in control groups. Pooled odds ratios (ORs) and 95% confidence intervals (CIs) were estimated using common-effect and random-effects models, with between-study heterogeneity evaluated using Cochran's Q test and the I^2 statistic **[1-12]**

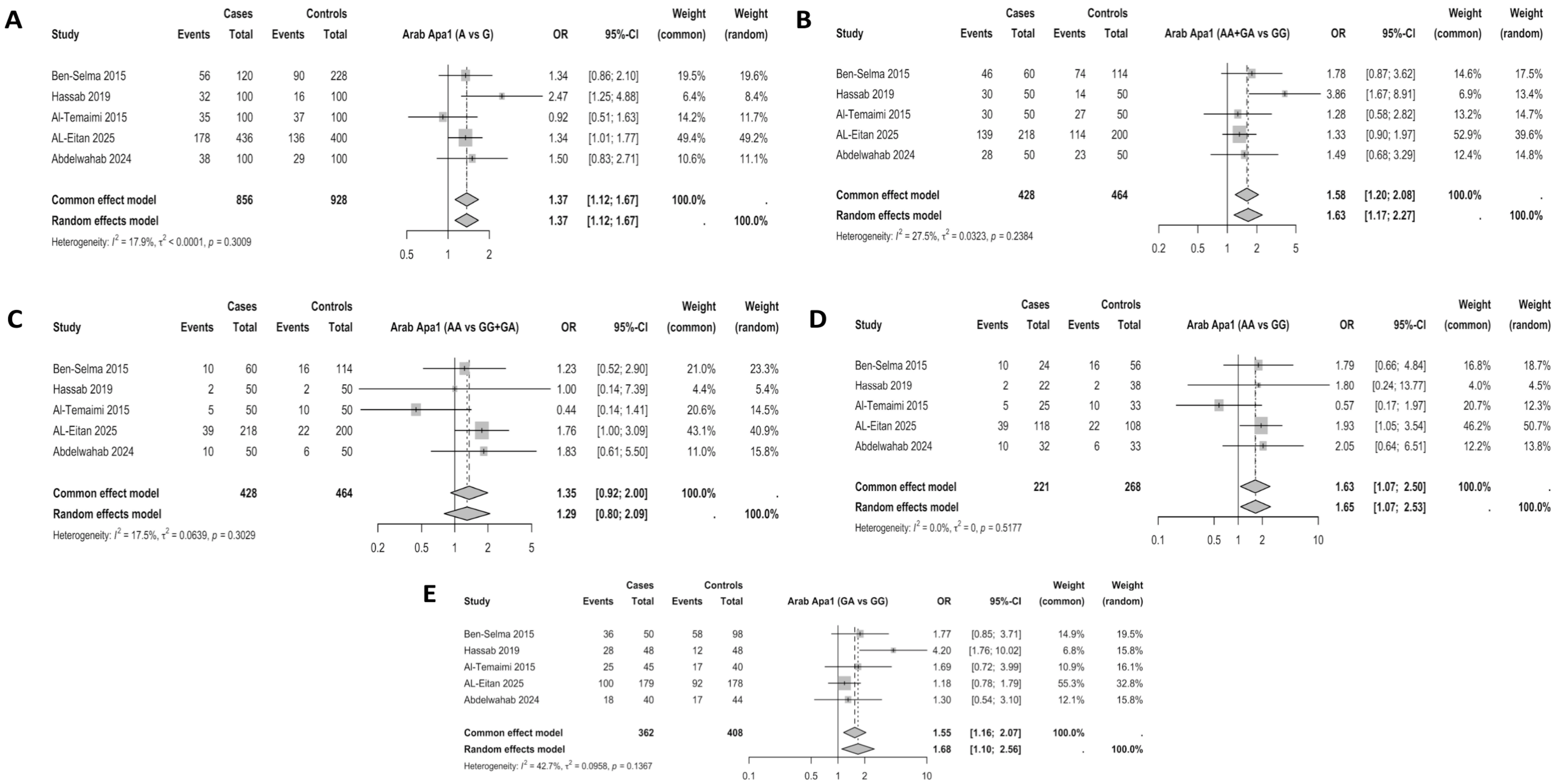

Figure S6. Association between the VDR ApaI polymorphism and multiple sclerosis risk in Arab populations across genetic models. Forest plots present pooled odds ratios (ORs) and 95% confidence intervals (CIs) for the association between the vitamin D receptor (VDR) ApaI polymorphism and multiple sclerosis (MS) susceptibility in Arab populations across five genetic models: **(A)** allelic (A vs G), **(B)** dominant (AA+AG vs GG), **(C)** recessive (AA vs AG+GG), **(D)** homozygous (AA vs GG), and **(E)** heterozygous (AG vs GG). Individual study estimates are shown as squares proportional to study weight, with horizontal lines representing 95% CIs. Diamonds indicate pooled estimates derived under common-effect and random-effects models. Between-study heterogeneity was assessed using Cochran's Q test and quantified with the I^2 statistic **[1,8-11]**

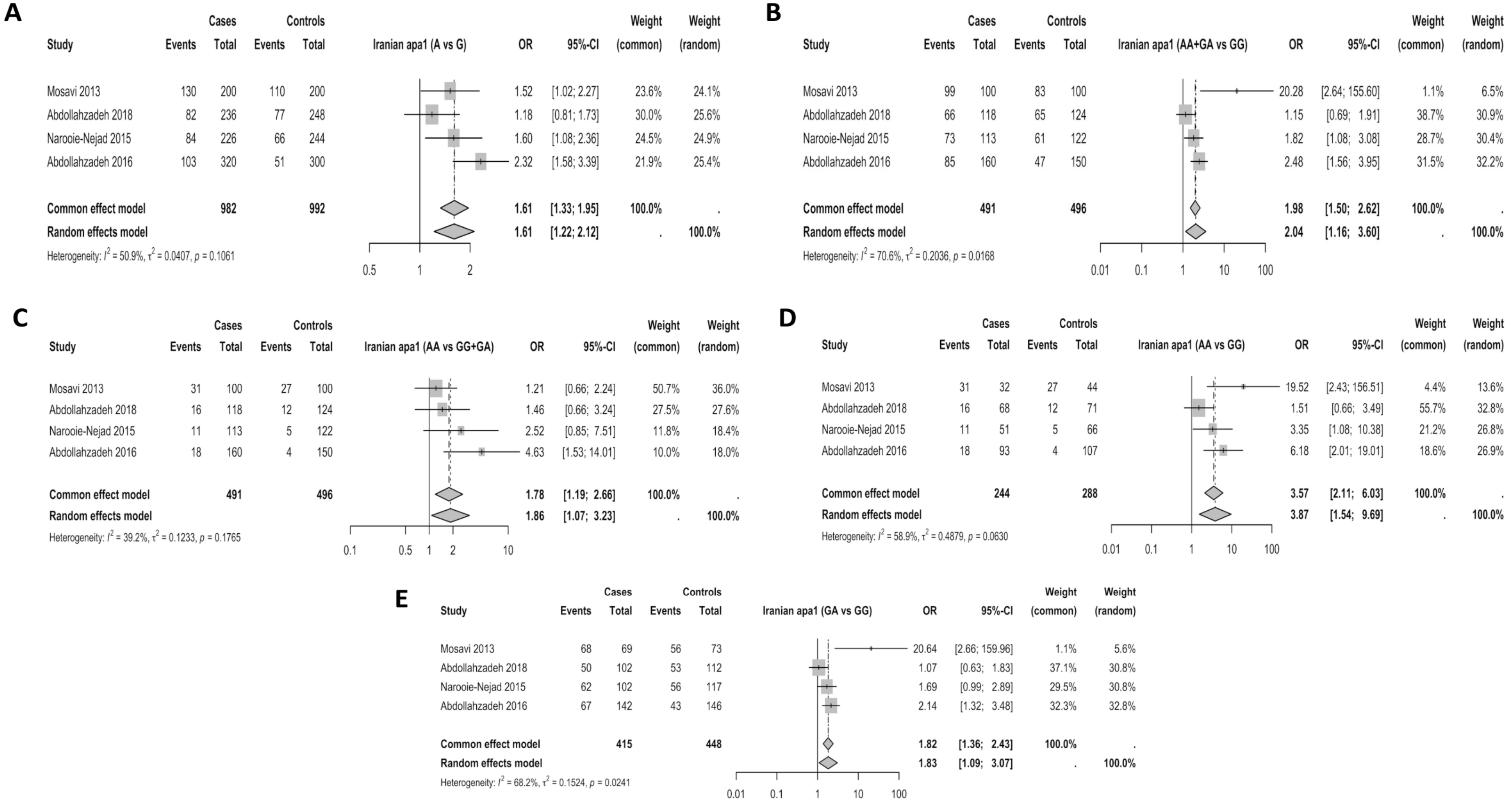

Figure S7. Association between the VDR ApaI polymorphism and multiple sclerosis risk in Iranian populations across genetic models. Forest plots display pooled odds ratios (ORs) and 95% confidence intervals (CIs) for the association between the vitamin D receptor (VDR) ApaI polymorphism and multiple sclerosis (MS) susceptibility in Iranian populations across five genetic models: **(A)** allelic (A vs G), **(B)** dominant (AA+AG vs GG), **(C)** recessive (AA vs AG+GG), **(D)** homozygous (AA vs GG), and **(E)** heterozygous (AG vs GG). Individual study estimates are represented by squares proportional to study weight, with horizontal lines indicating 95% CIs. Diamonds represent pooled estimates derived under common-effect and random-effects models. Between-study heterogeneity was evaluated using Cochran's Q test and quantified using the I^2 statistic **[3,4,6,7]**

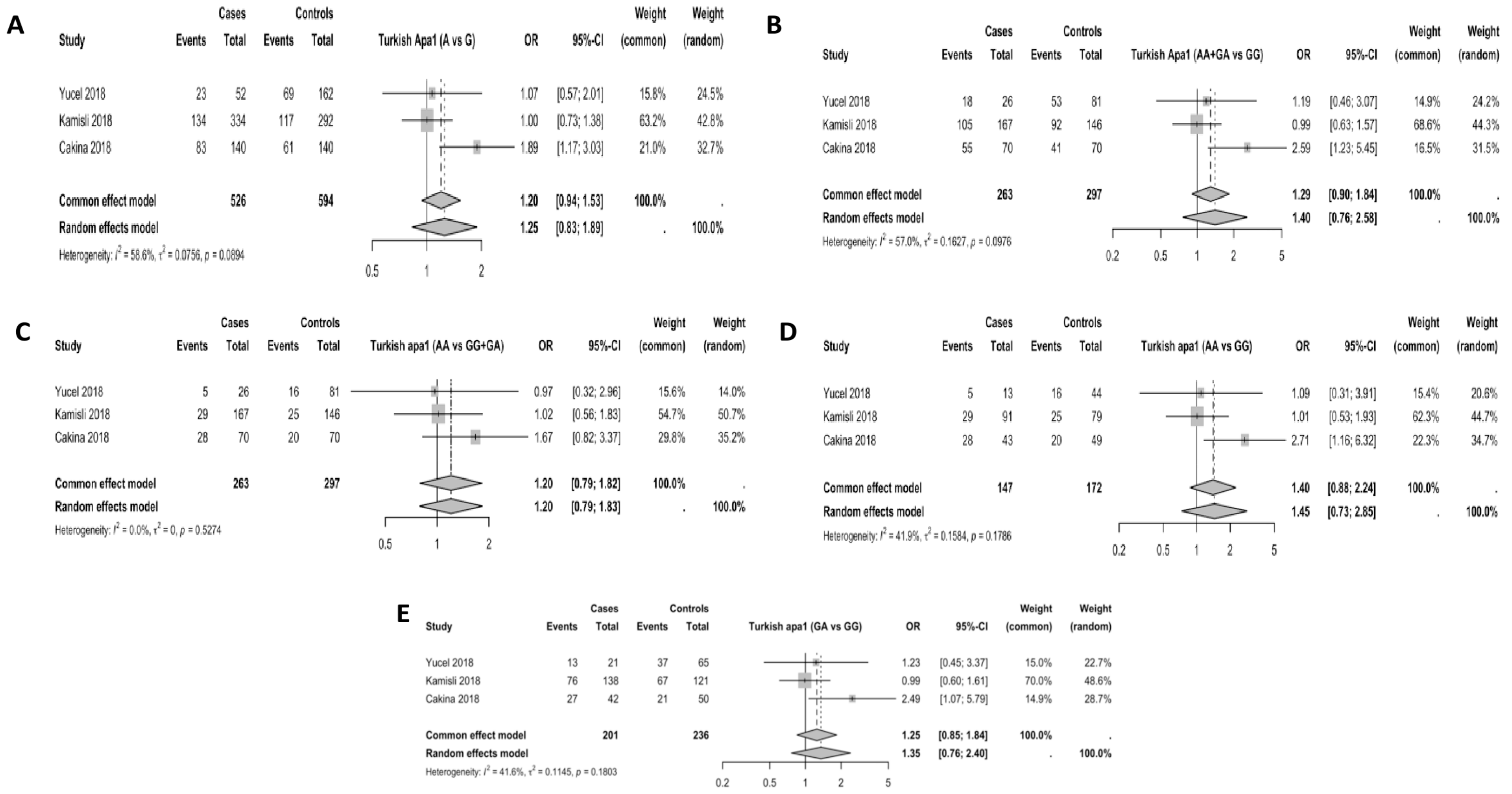

Figure S8. Association between the VDR ApaI polymorphism and multiple sclerosis risk in Turkish populations across genetic models. Forest plots illustrate pooled odds ratios (ORs) and 95% confidence intervals (CIs) for the association between the vitamin D receptor (VDR) ApaI polymorphism and multiple sclerosis (MS) susceptibility in Turkish populations across five genetic models: **(A)** allelic (A vs G), **(B)** dominant (AA+AG vs GG), **(C)** recessive (AA vs AG+GG), **(D)** homozygous (AA vs GG), and **(E)** heterozygous (AG vs GG). Individual study estimates are displayed as squares proportional to study weight, with horizontal lines indicating 95% CIs. Diamonds represent pooled estimates derived under common-effect and random-effects models. Between-study heterogeneity was assessed using Cochran's Q test and quantified with the I² statistic **[2,5,12]**.

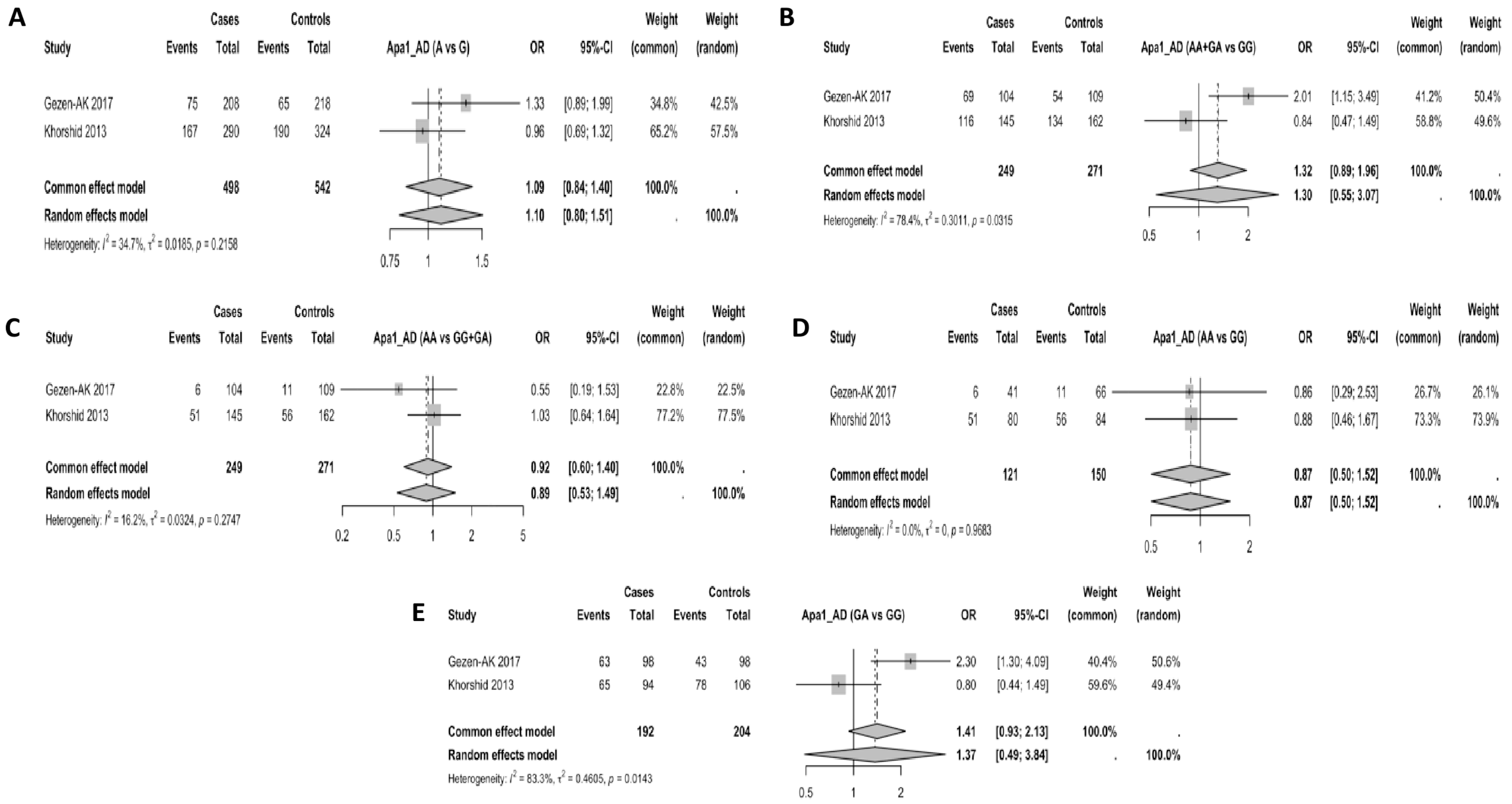

Figure S9. Association between the VDR ApaI polymorphism and Alzheimer's disease risk across genetic models. Forest plots present pooled odds ratios (ORs) and 95% confidence intervals (CIs) for the association between the vitamin D receptor (VDR) ApaI polymorphism and susceptibility to Alzheimer's disease (AD) across five genetic models: **(A)** allelic (A vs G), **(B)** dominant (AA+AG vs GG), **(C)** recessive (AA vs AG+GG), **(D)** homozygous (AA vs GG), and **(E)** heterozygous (AG vs GG). Individual study estimates are shown as squares proportional to study weight, with horizontal lines indicating 95% CIs. Diamonds represent pooled estimates derived under common-effect and random-effects models. Between-study heterogeneity was assessed using Cochran's Q test and quantified using the I² statistic **[13,14]**

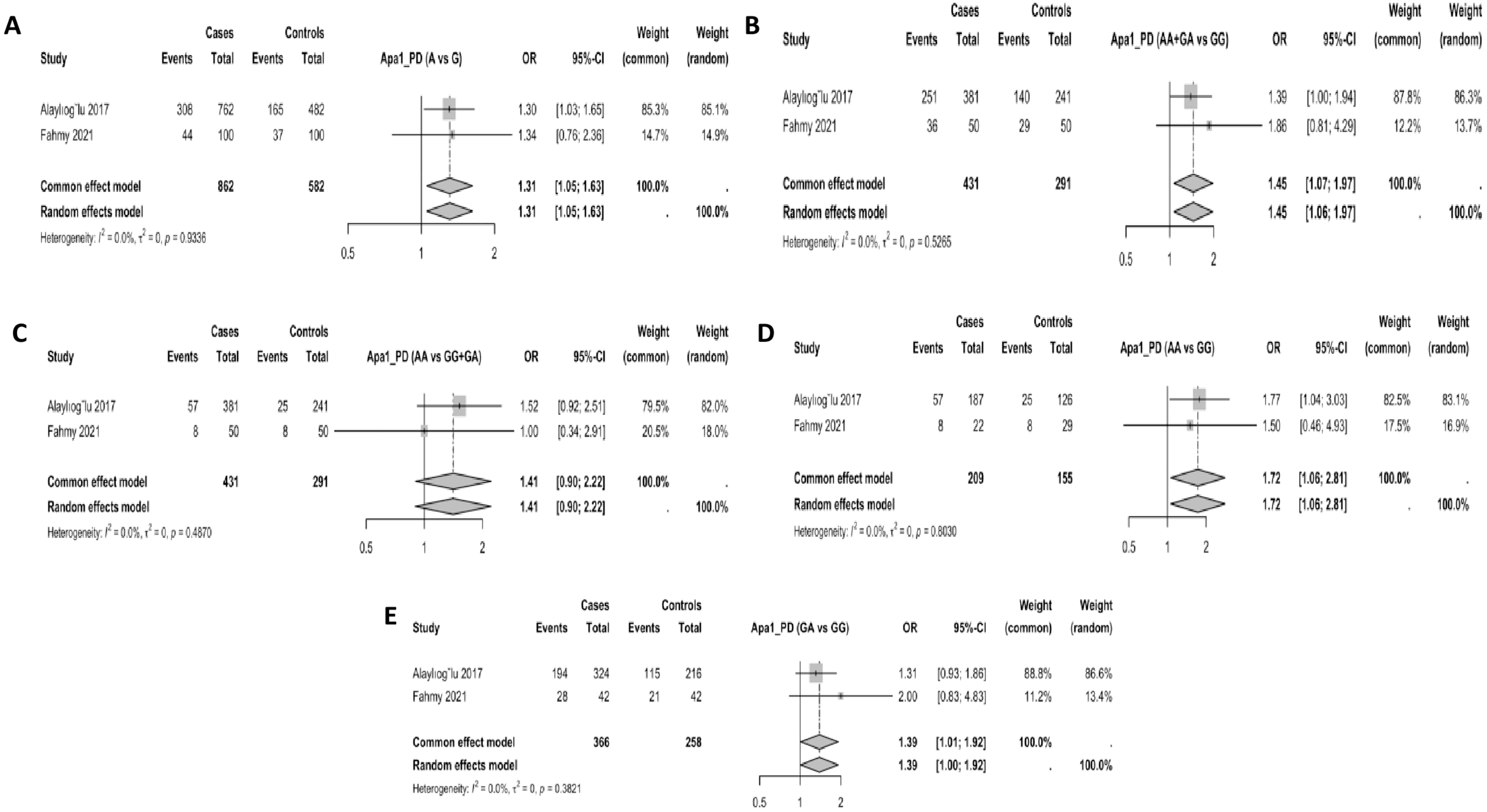

Figure S10. Association between the VDR ApaI polymorphism and Parkinson's disease risk across genetic models. Forest plots present pooled odds ratios (ORs) and 95% confidence intervals (CIs) for the association between the vitamin D receptor (VDR) ApaI polymorphism and susceptibility to Parkinson's disease (PD) across five genetic models: **(A)** allelic (A vs G), **(B)** dominant (AA+AG vs GG), **(C)** recessive (AA vs AG+GG), **(D)** homozygous (AA vs GG), and **(E)** heterozygous (AG vs GG). Individual study estimates are shown as squares proportional to study weight, with horizontal lines indicating 95% CIs. Diamonds represent pooled estimates derived under common-effect and random-effects models. Between-study heterogeneity was assessed using Cochran's Q test and quantified using the I^2 statistic **[15,16]**

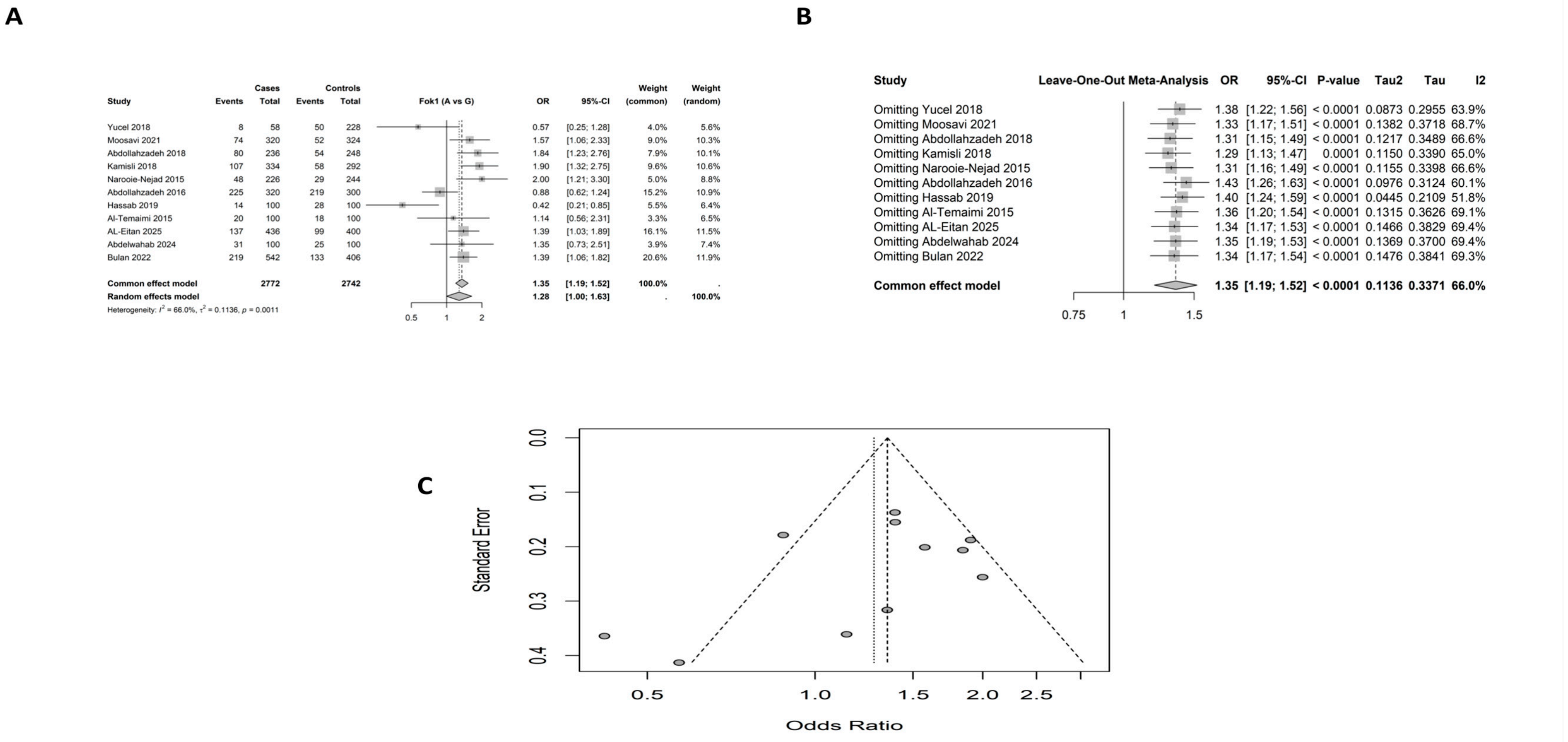

Figure S11. VDR FokI allelic model analyses and multiple sclerosis risk in the overall population. These analyses support the main meta-analysis of the association between the vitamin D receptor (VDR) FokI polymorphism and multiple sclerosis (MS) risk under the allelic genetic model (A vs G) in the overall population. (A) Forest plot of individual studies and pooled estimates. (B) Leave-one-out sensitivity analysis assessing the influence of each study on the pooled effect estimate. (C) Funnel plot evaluating small-study effects and potential publication bias. Pooled odds ratios (ORs) and 95% confidence intervals (CIs) were estimated using common-effect and random-effects models, with between-study heterogeneity assessed using Cochran's Q test and quantified by the I² statistic **[2,4,5,7-9,17-19]**.

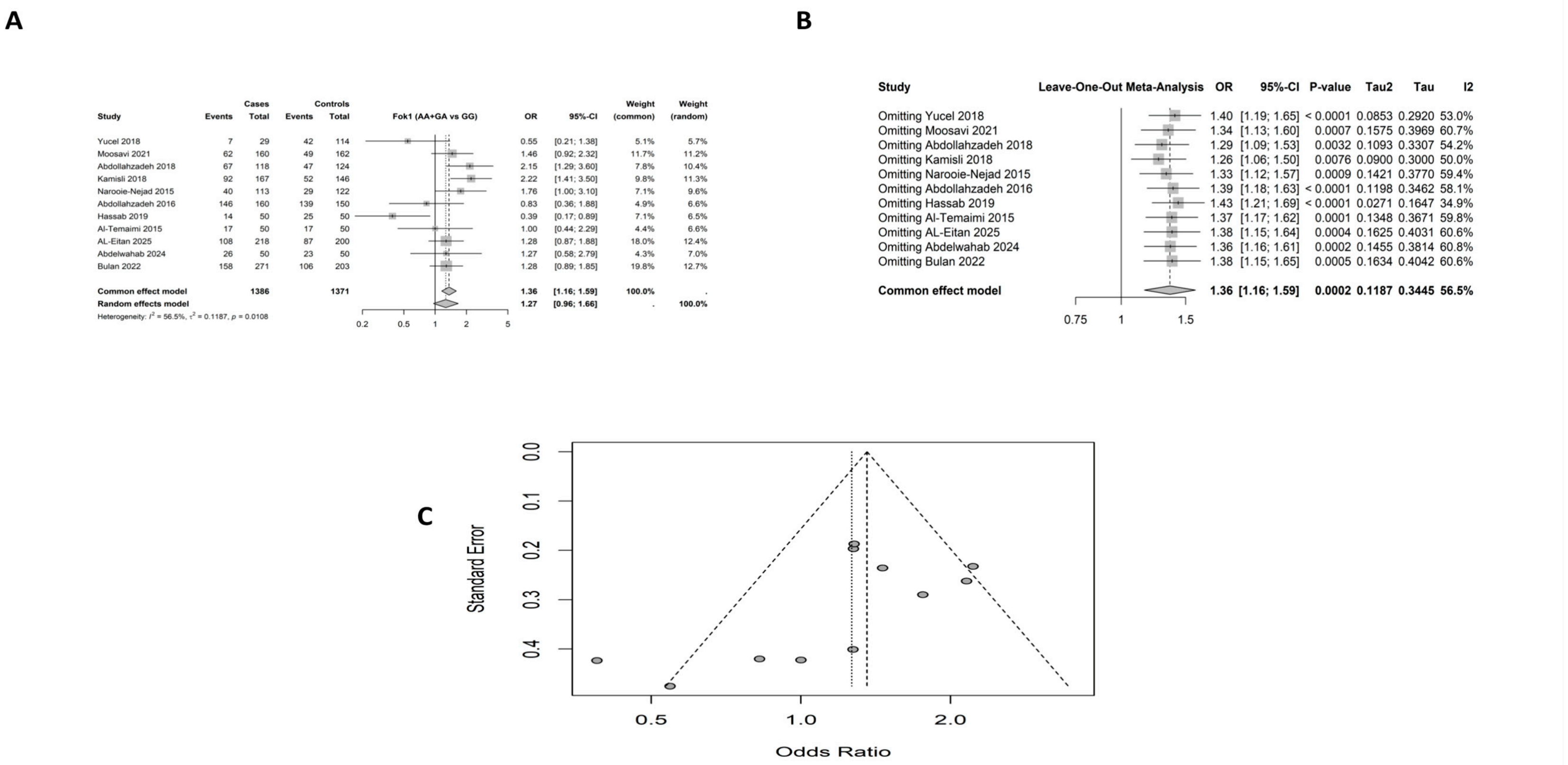

Figure S12. VDR FokI dominant model analyses and multiple sclerosis risk in the overall population. Association analysis of the vitamin D receptor (VDR) FokI polymorphism with multiple sclerosis (MS) risk under a dominant genetic model (AA+AG vs GG) in the overall population. (A) Forest plot of individual studies and pooled effect estimates. (B) Leave-one-out sensitivity analysis assessing the influence of each study on the pooled estimate. (C) Funnel plot evaluating small-study effects and potential publication bias. Pooled odds ratios (ORs) and 95% confidence intervals (CIs) were estimated using common-effect and random-effects models, with between-study heterogeneity assessed using Cochran's Q test and quantified by the I^2 statistic **[2,4,5,7-9,17-19]**.

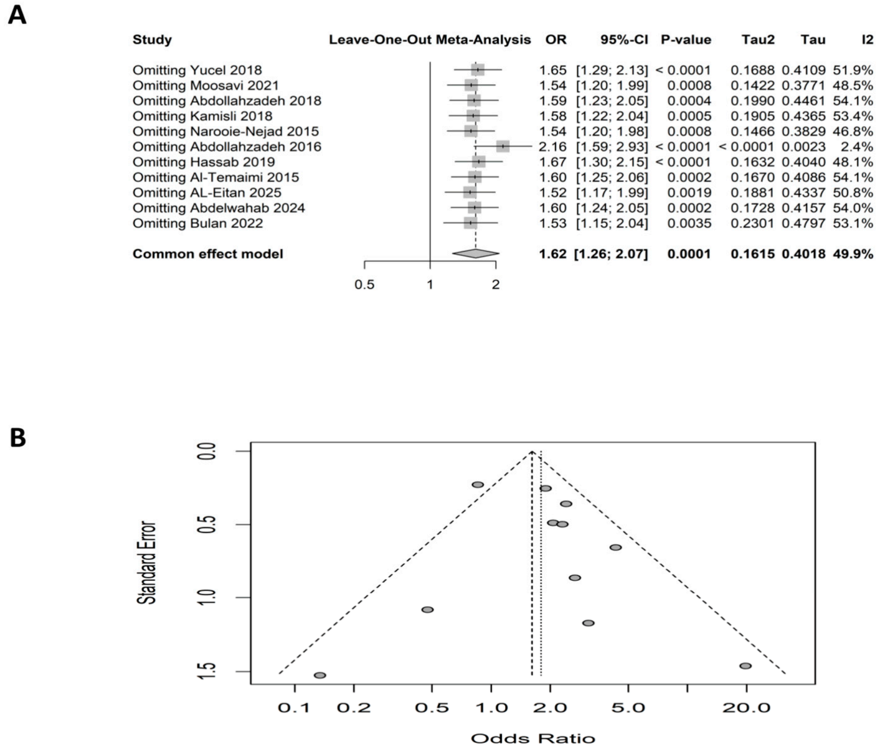

Figure S13. VDR FokI recessive model analyses and multiple sclerosis risk in the overall population. Association analysis of the vitamin D receptor (VDR) FokI polymorphism with multiple sclerosis (MS) risk under a recessive genetic model (AA vs AG+GG) in the overall population. (A) Leave-one-out sensitivity analysis assessing the influence of individual studies on the pooled effect estimate. (B) Funnel plot evaluating small-study effects and potential publication bias. Pooled odds ratios (ORs) and 95% confidence intervals (CIs) were estimated using common-effect and random-effects models, with between-study heterogeneity assessed using Cochran's Q test and quantified by the I^2 statistic **[2,4,5,7-9,17-19]**.

A

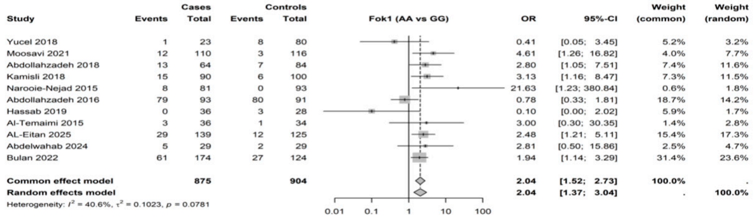

B

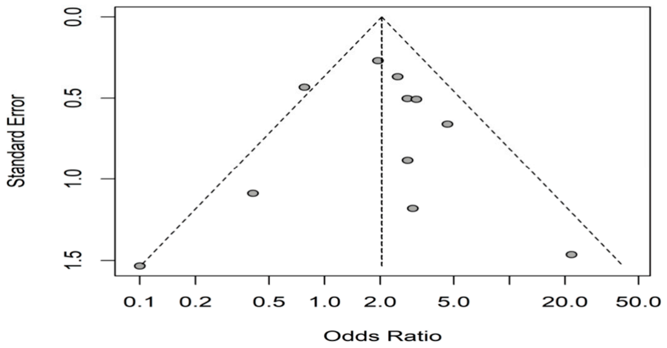

Figure S14. VDR FokI homozygous model analyses and multiple sclerosis risk in the overall population. Association analysis between the vitamin D receptor (VDR) FokI polymorphism and multiple sclerosis (MS) risk under the homozygous genetic model (AA vs GG) in the overall population. (A) Forest plot of individual studies and pooled effect estimates. (B) Funnel plot evaluating small-study effects and potential publication bias. Pooled odds ratios (ORs) and 95% confidence intervals (CIs) were estimated using common-effect and random-effects models, with between-study heterogeneity assessed using Cochran's Q test and quantified by the I^2 statistic **[2,4,5,7-9,17-19]**.

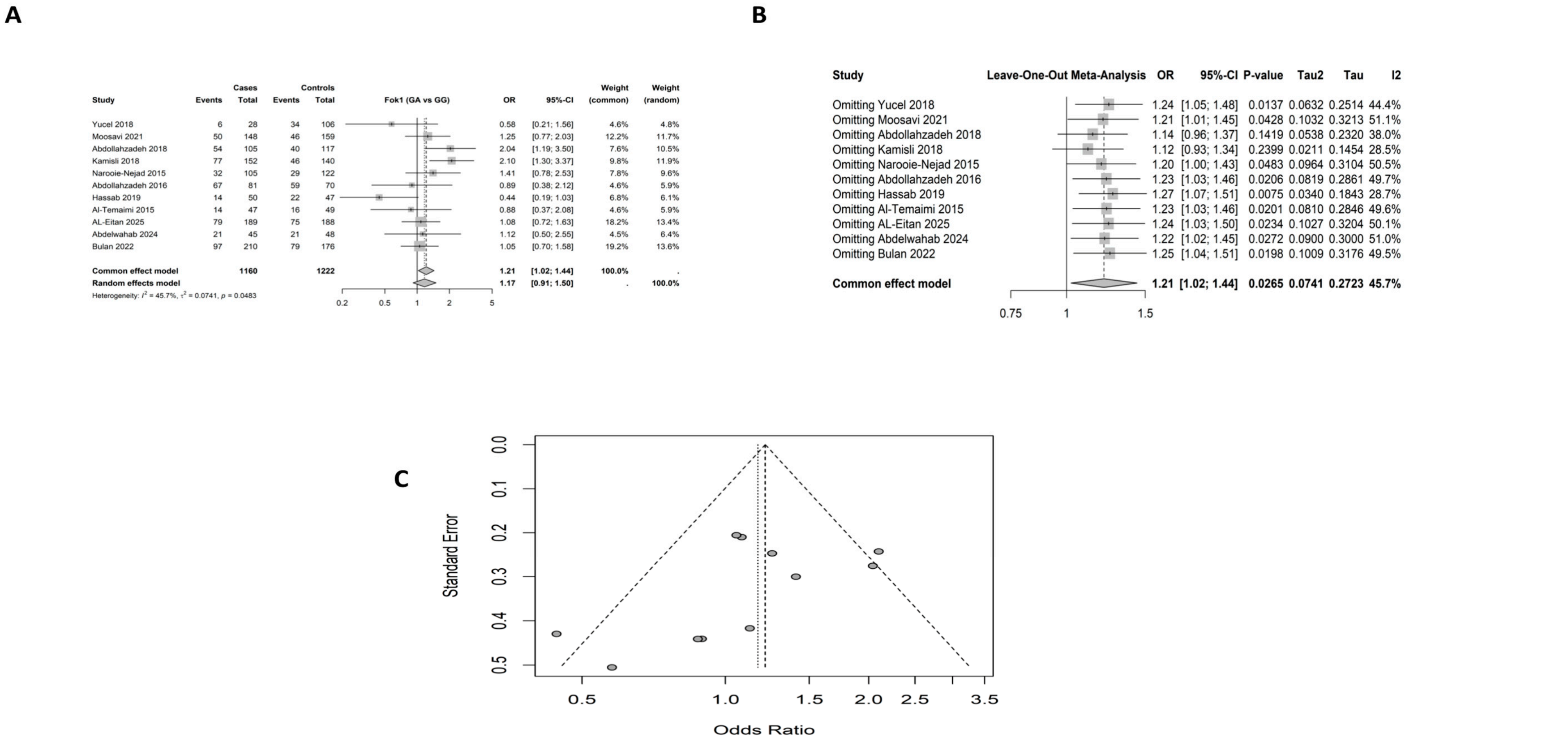

Figure S15. VDR FokI heterozygous model analyses and multiple sclerosis risk in the overall population. Association analysis between the vitamin D receptor (VDR) FokI polymorphism and multiple sclerosis (MS) risk under the heterozygous genetic model (GA vs GG) in the overall population. (A) Forest plot of individual studies and pooled effect estimates. (B) Leave-one-out sensitivity analysis assessing the influence of each study on the pooled estimate. (C) Funnel plot evaluating small-study effects and potential publication bias. Pooled odds ratios (ORs) and 95% confidence intervals (CIs) were estimated using common-effect and random-effects models, with between-study heterogeneity assessed using Cochran's Q test and quantified by the I^2 statistic **[2,4,5,7-9,17-19]**.

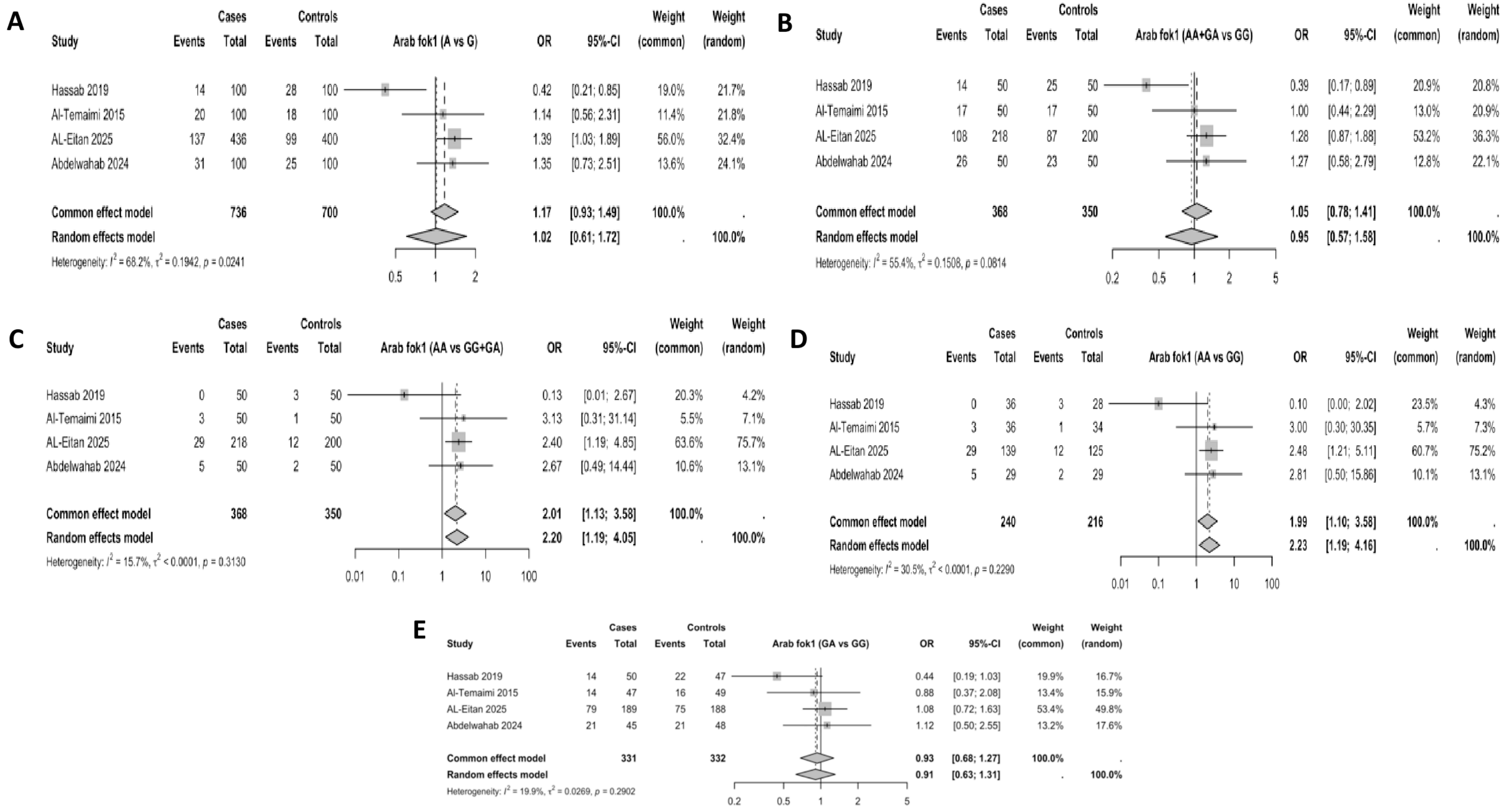

Figure S16. Association between the VDR FokI polymorphism and multiple sclerosis risk in Arab populations across genetic models. Forest plots present pooled odds ratios (ORs) and 95% confidence intervals (CIs) for the association between the vitamin D receptor (VDR) FokI polymorphism and multiple sclerosis (MS) susceptibility in Arab populations across five genetic models: (A) allelic (A vs G), (B) dominant (AA+AG vs GG), (C) recessive (AA vs AG+GG), (D) homozygous (AA vs GG), and (E) heterozygous (AG vs GG). Individual study estimates are shown as squares proportional to study weight, with diamonds representing pooled estimates derived under common-effect and random-effects models. Between-study heterogeneity was assessed using Cochran's Q test and the I^2 statistic **[8-11]**.

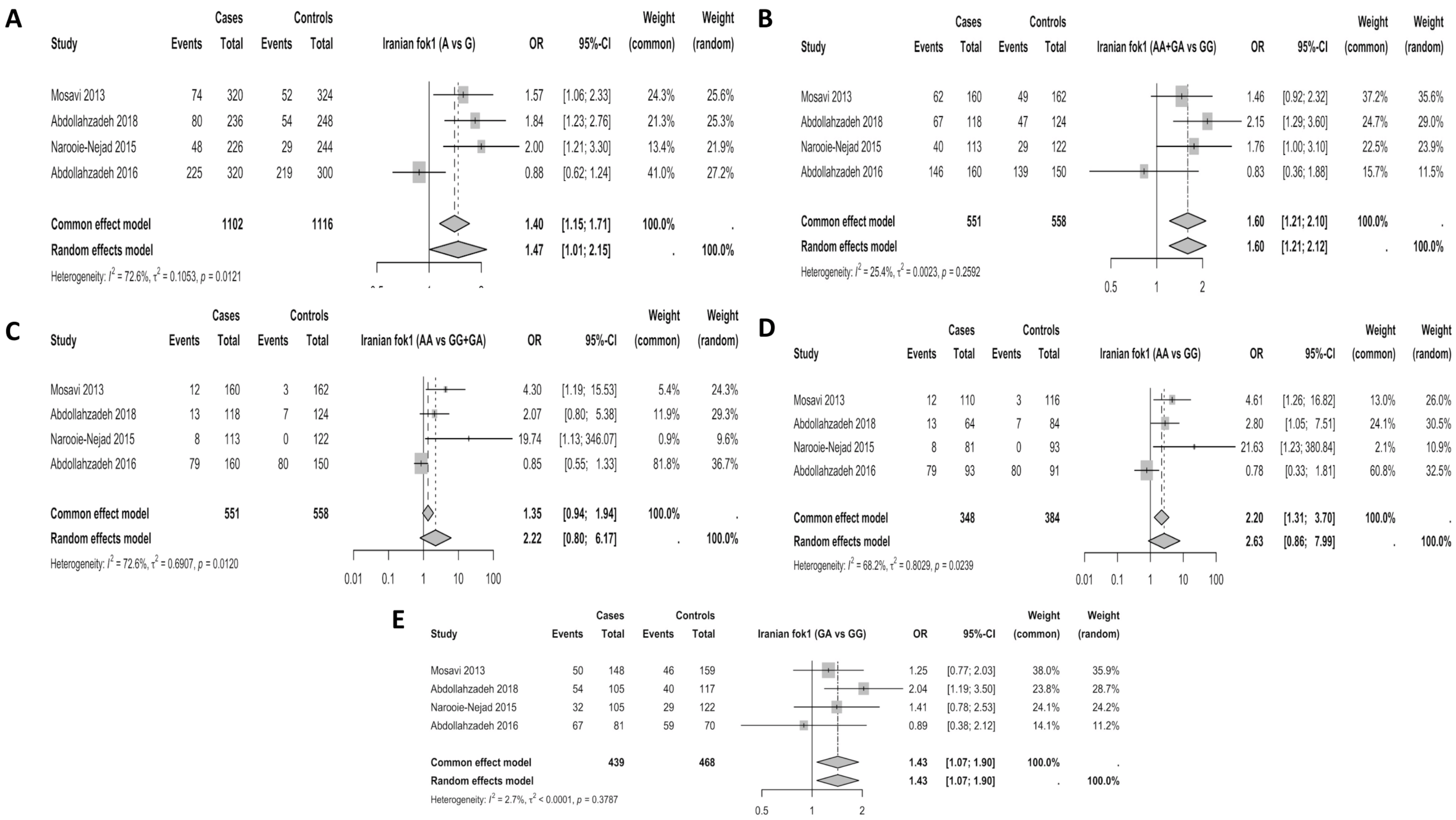

Figure S17. Association between the VDR FokI polymorphism and multiple sclerosis risk in Iranian populations across genetic models. Forest plots illustrate pooled ORs and 95% CIs for the association between the VDR FokI polymorphism and MS risk in Iranian populations across allelic, dominant, recessive, homozygous, and heterozygous genetic models (**A–E**). Squares indicate study-specific effect estimates weighted by study precision, while diamonds denote pooled effects from common-effect and random-effects models. Statistical heterogeneity was evaluated using Cochran's Q test and quantified with the I² statistic **[3,4,7,18]**.

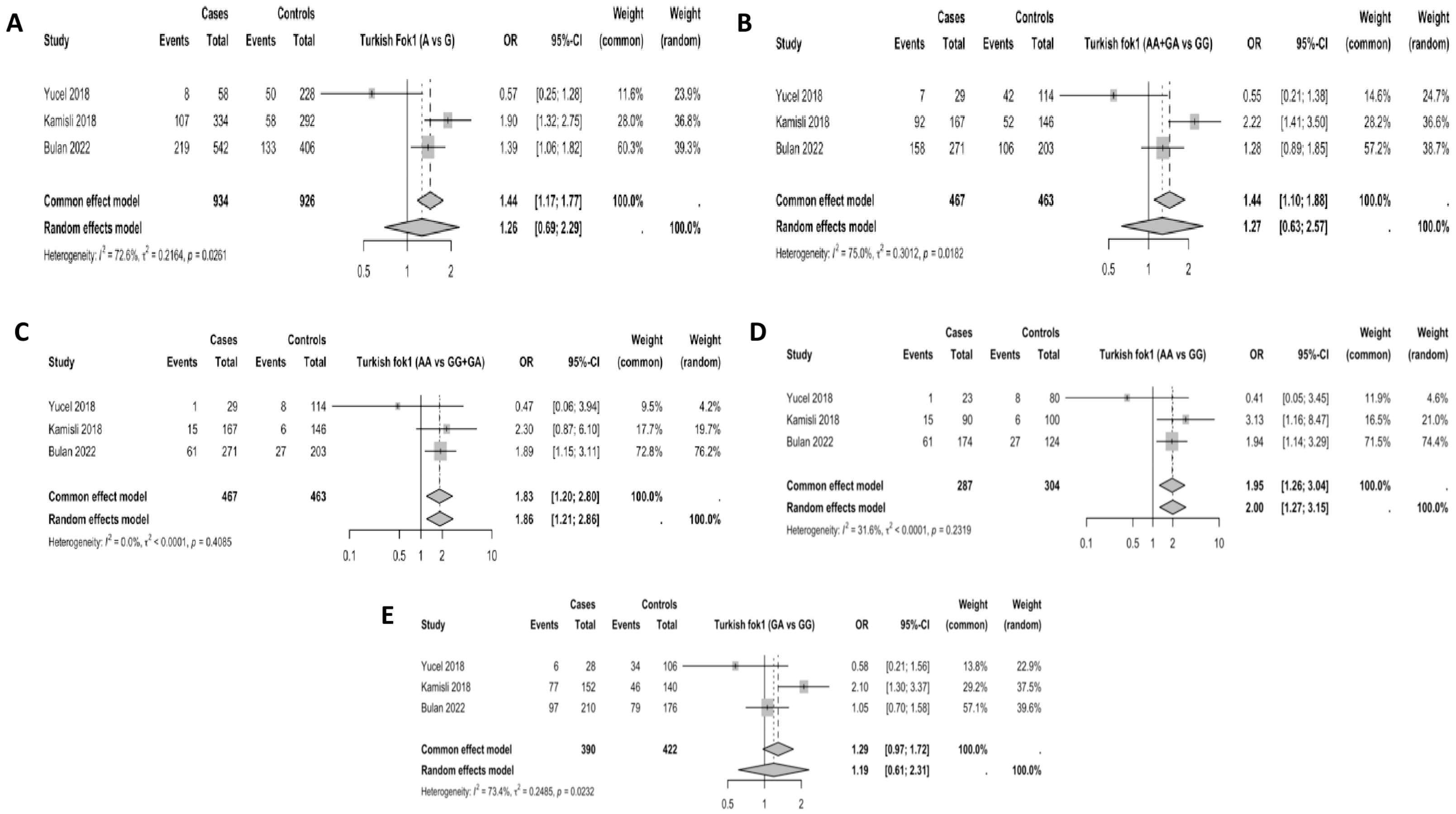

Figure S18. Association between the VDR FokI polymorphism and multiple sclerosis risk in Turkish populations across genetic models. Forest plots show pooled ORs and 95% CIs for the association between the VDR FokI polymorphism and MS susceptibility in Turkish populations under five genetic models: allelic, dominant, recessive, homozygous, and heterozygous (**A–E**). Individual and pooled effect estimates are displayed using common-effect and random-effects models, with between-study heterogeneity assessed by Cochran's Q test and the I² statistic **[2,5,19]**.

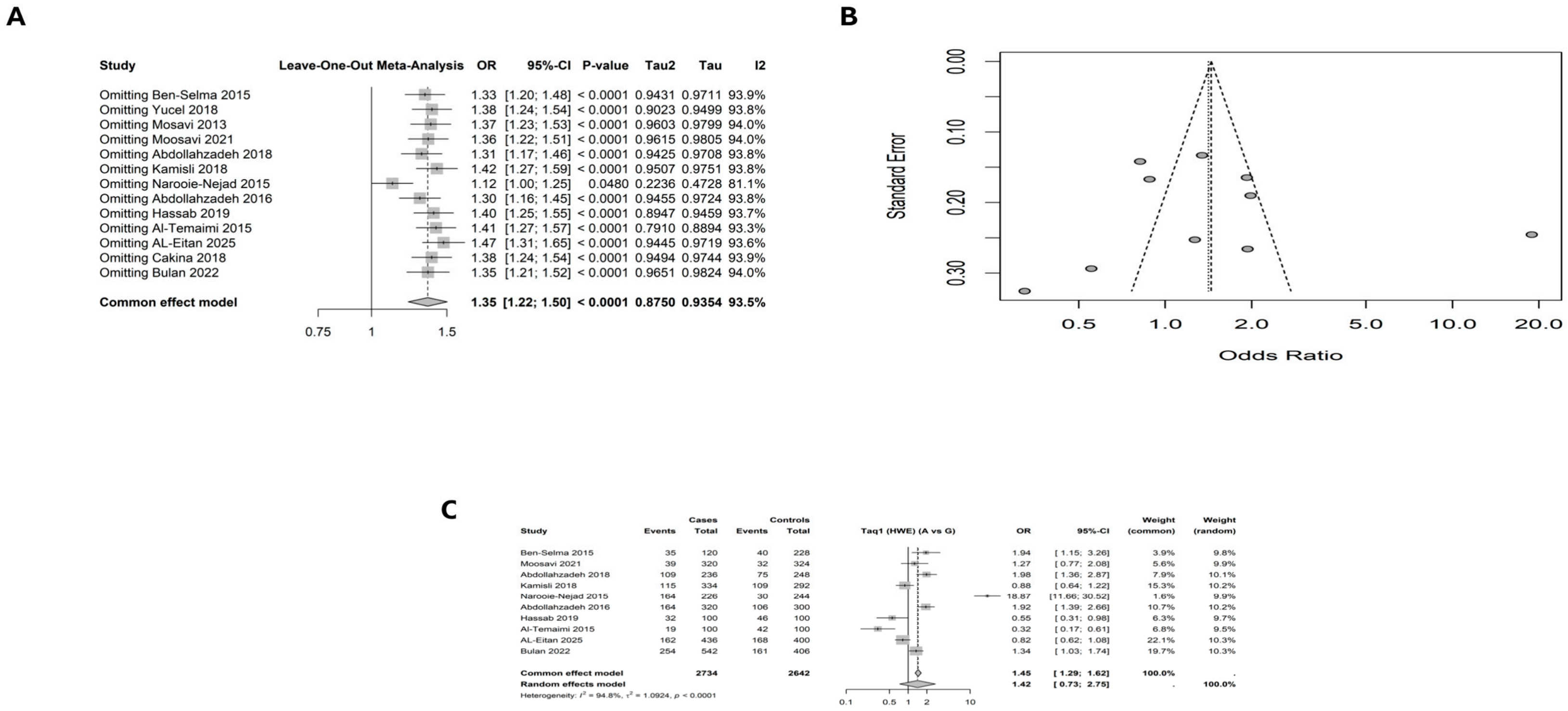

Figure S19. VDR Taq1 allelic model analyses and multiple sclerosis risk in the overall population. Association analysis between the vitamin D receptor (VDR) TaqI polymorphism and multiple sclerosis (MS) risk under the allelic genetic model (A vs G) in the overall population. **(A)** Leave-one-out sensitivity analysis evaluating the influence of individual studies on the pooled effect estimate. **(B)** Funnel plot assessing small-study effects and potential publication bias. **(C)** Forest plot restricted to studies in which control groups conformed to Hardy–Weinberg equilibrium (HWE). Pooled odds ratios (ORs) and 95% confidence intervals (CIs) were estimated using common-effect and random-effects models, with between-study heterogeneity assessed using Cochran's Q test and quantified by the I^2 statistic **[1-10,12,17,19]**.

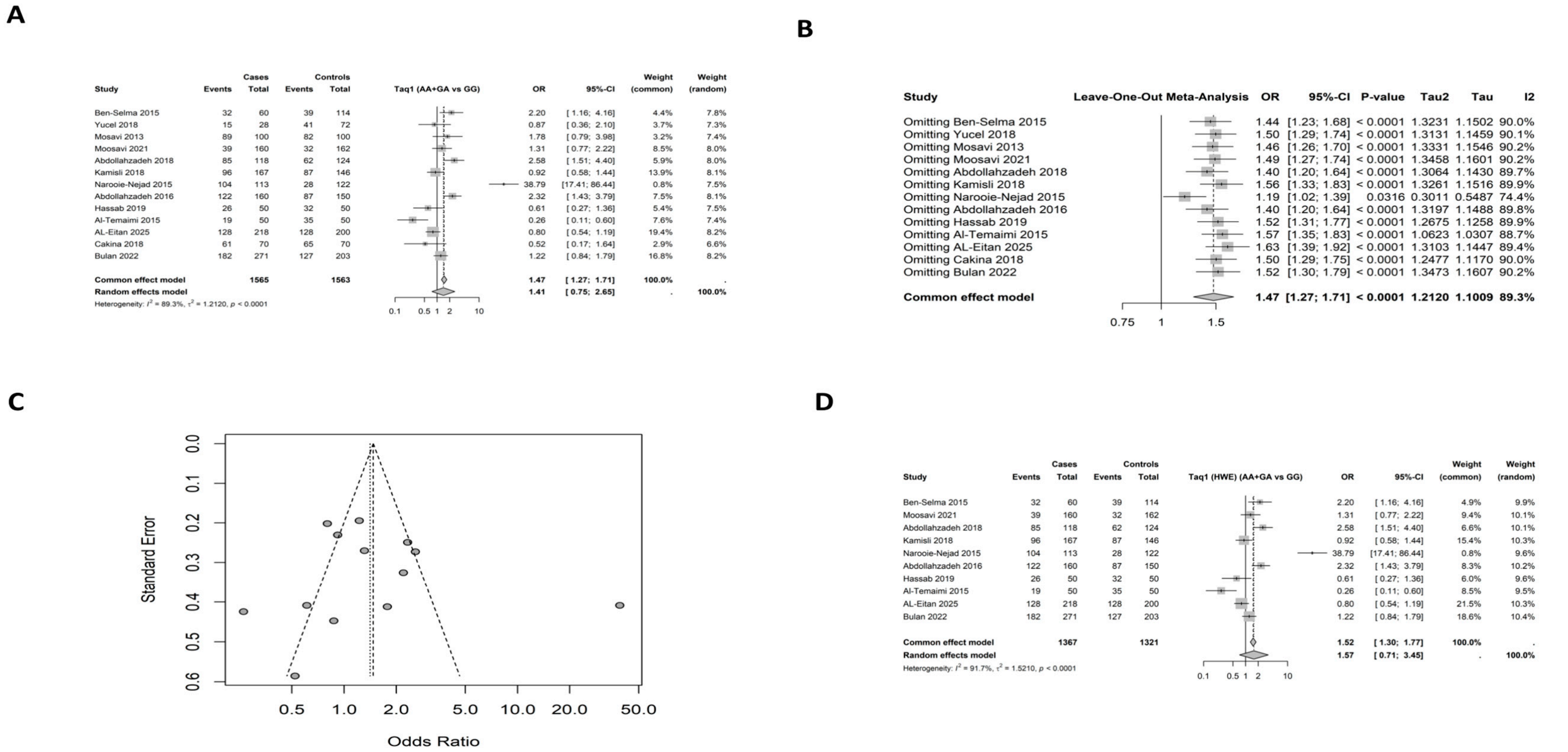

Figure S20. VDR Taq1 dominant model analyses and multiple sclerosis risk in the overall population. Association analysis of the vitamin D receptor (VDR) TaqI polymorphism with multiple sclerosis (MS) risk under a dominant genetic model (AA+GA vs GG) in the overall population. **(A)** Forest plot of individual studies and pooled effect estimates. **(B)** Leave-one-out sensitivity analysis assessing the influence of each study on the pooled estimate. **(C)** Funnel plot evaluating small-study effects and potential publication bias. **(D)** Forest plot restricted to studies in which control groups conformed to Hardy–Weinberg equilibrium (HWE). Pooled odds ratios (ORs) and 95% confidence intervals (CIs) were estimated using common-effect and random-effects models, with between-study heterogeneity assessed using Cochran's Q test and quantified by the I^2 statistic **[1-10,12,17,19]**.

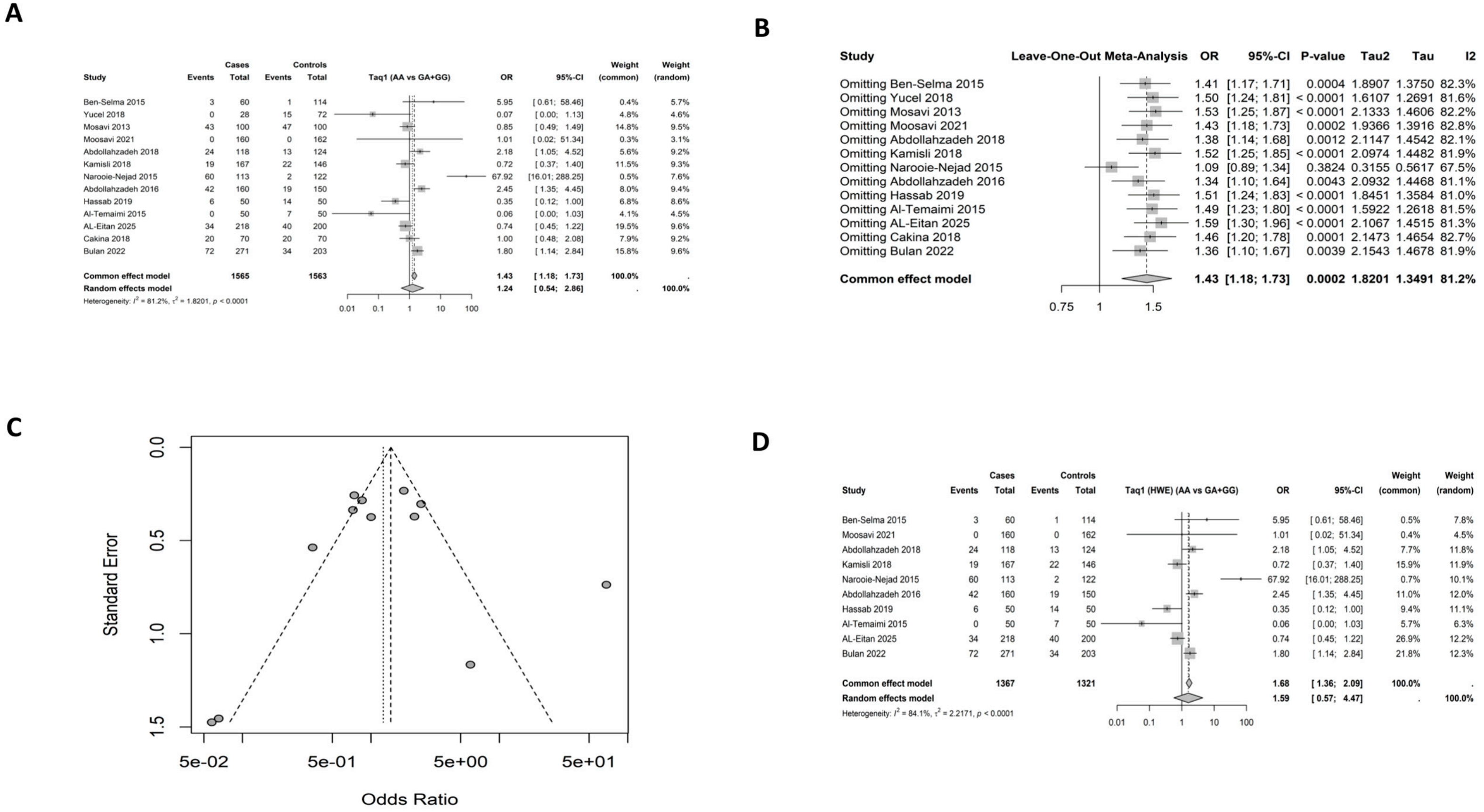

Figure S21. VDR Taq1 recessive model analyses and multiple sclerosis risk in the overall population. Association analysis between the vitamin D receptor (VDR) TaqI polymorphism and multiple sclerosis (MS) risk under the recessive genetic model (AA vs GA+GG) in the overall population. **(A)** Forest plot of individual studies and pooled effect estimates. **(B)** Leave-one-out sensitivity analysis assessing the influence of each study on the pooled estimate. **(C)** Funnel plot evaluating small-study effects and potential publication bias. **(D)** Forest plot restricted to studies in which control groups conformed to Hardy–Weinberg equilibrium (HWE). Pooled odds ratios (ORs) and 95% confidence intervals (CIs) were estimated using common-effect and random-effects models, with between-study heterogeneity assessed using Cochran's Q test and quantified by the I^2 statistic **[1-10,12,17,19]**.

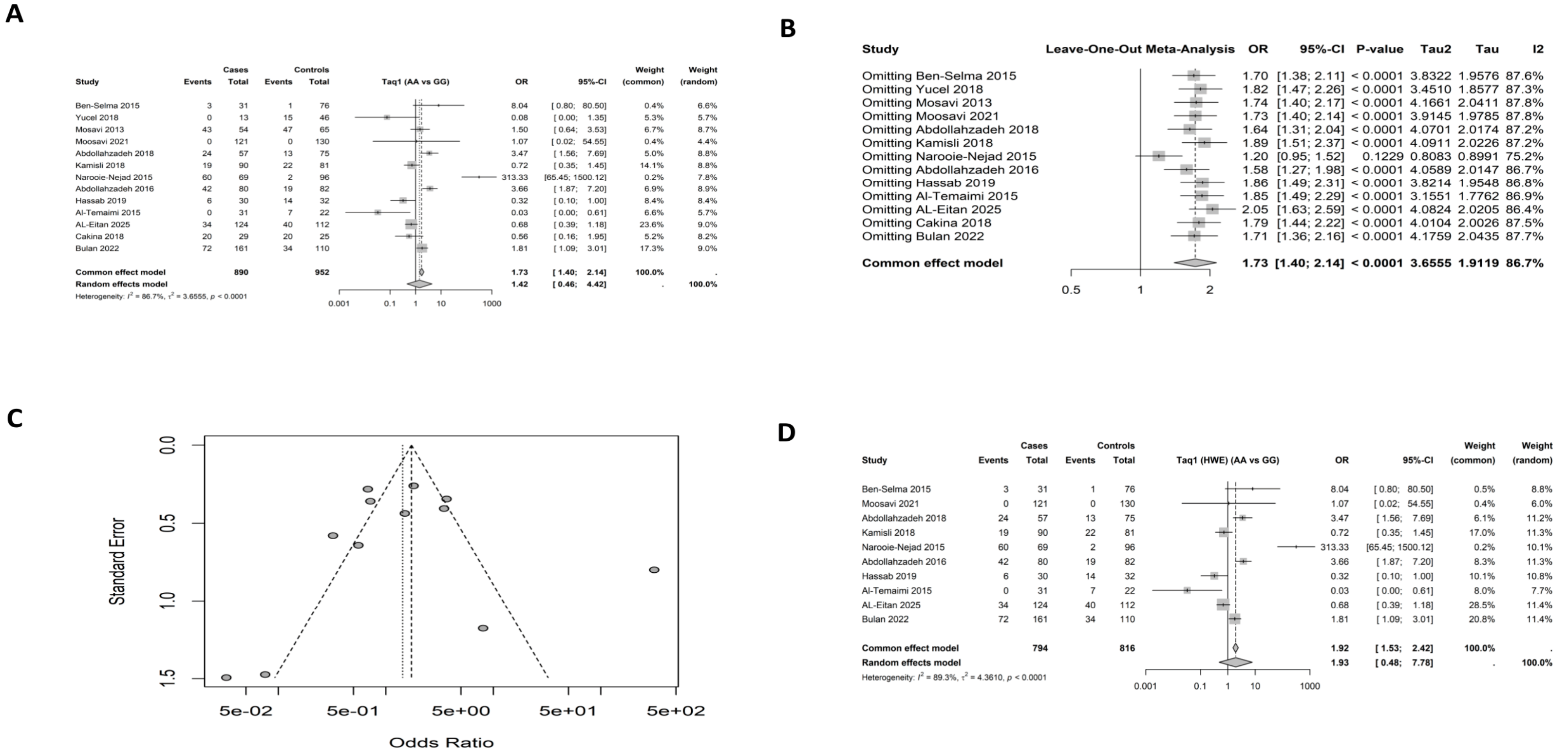

Figure S22. VDR Taq1 homozygous model analyses and multiple sclerosis risk in the overall population. Association analysis between the vitamin D receptor (VDR) TaqI polymorphism and multiple sclerosis (MS) risk under the homozygous genetic model (AA vs GG) in the overall population. **(A)** Forest plot of individual studies and pooled effect estimates. **(B)** Leave-one-out sensitivity analysis evaluating the influence of each study on the pooled estimate. **(C)** Funnel plot assessing small-study effects and potential publication bias. **(D)** Forest plot restricted to studies in which control groups conformed to Hardy–Weinberg equilibrium (HWE). Pooled odds ratios (ORs) and 95% confidence intervals (CIs) were estimated using common-effect and random-effects models, with between-study heterogeneity assessed using Cochran's Q test and quantified by the I^2 statistic **[1-10,12,17,19]**.

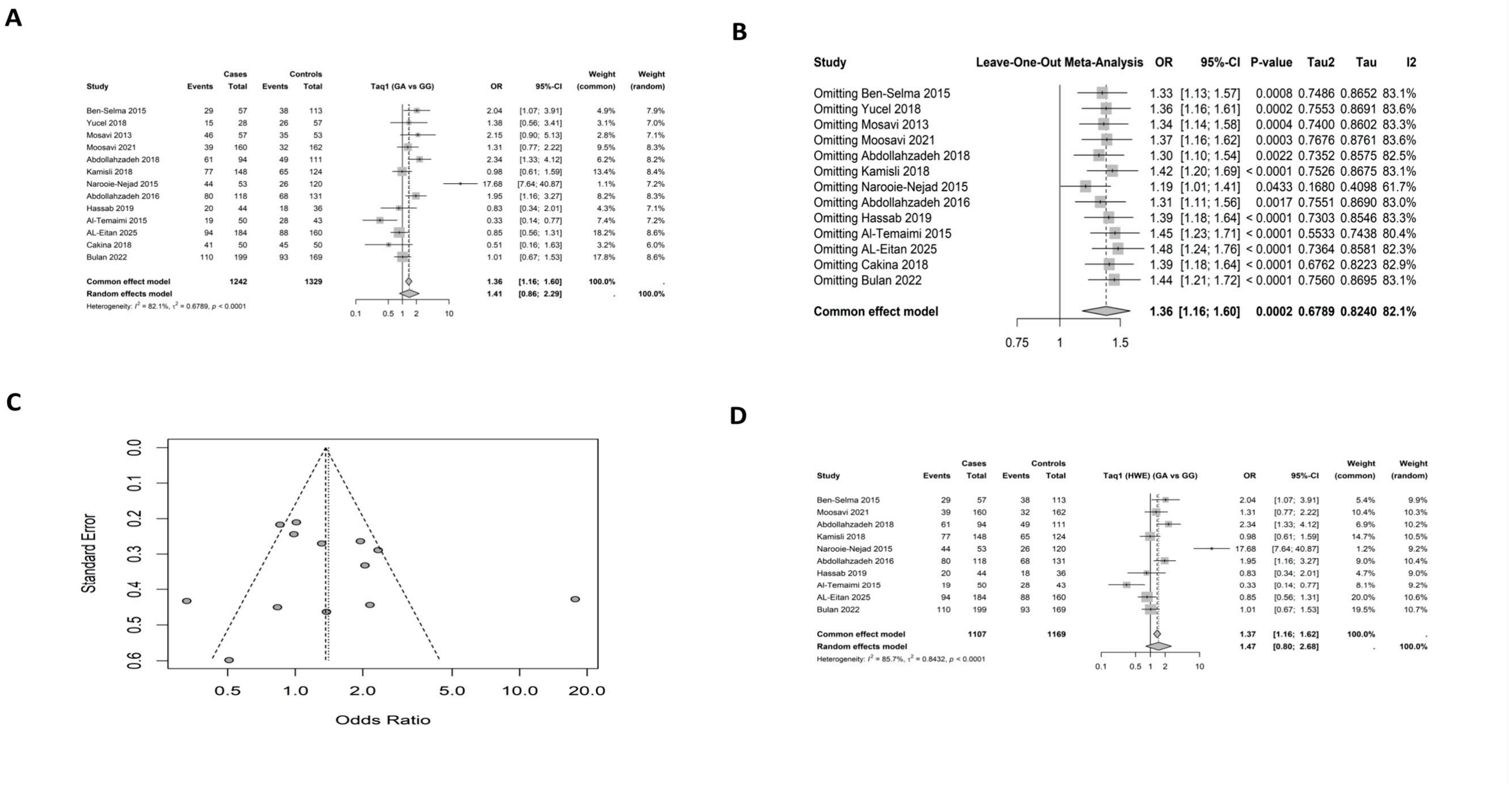

Figure S23. VDR Taq1 heterozygous model analyses and multiple sclerosis risk in the overall population. Association analysis of the vitamin D receptor (VDR) TaqI polymorphism with multiple sclerosis (MS) risk under the heterozygous genetic model (GA vs GG) in the overall population. **(A)** Forest plot of individual studies and pooled effect estimates. **(B)** Leave-one-out sensitivity analysis evaluating the influence of each study on the pooled estimate. **(C)** Funnel plot assessing small-study effects and potential publication bias. **(D)** Forest plot restricted to studies in which control groups conformed to Hardy–Weinberg equilibrium (HWE). Pooled odds ratios (ORs) and 95% confidence intervals (CIs) were estimated using common-effect and random-effects models, with between-study heterogeneity assessed using Cochran's Q test and quantified by the I^2 statistic **[1-10,12,17,19]**.

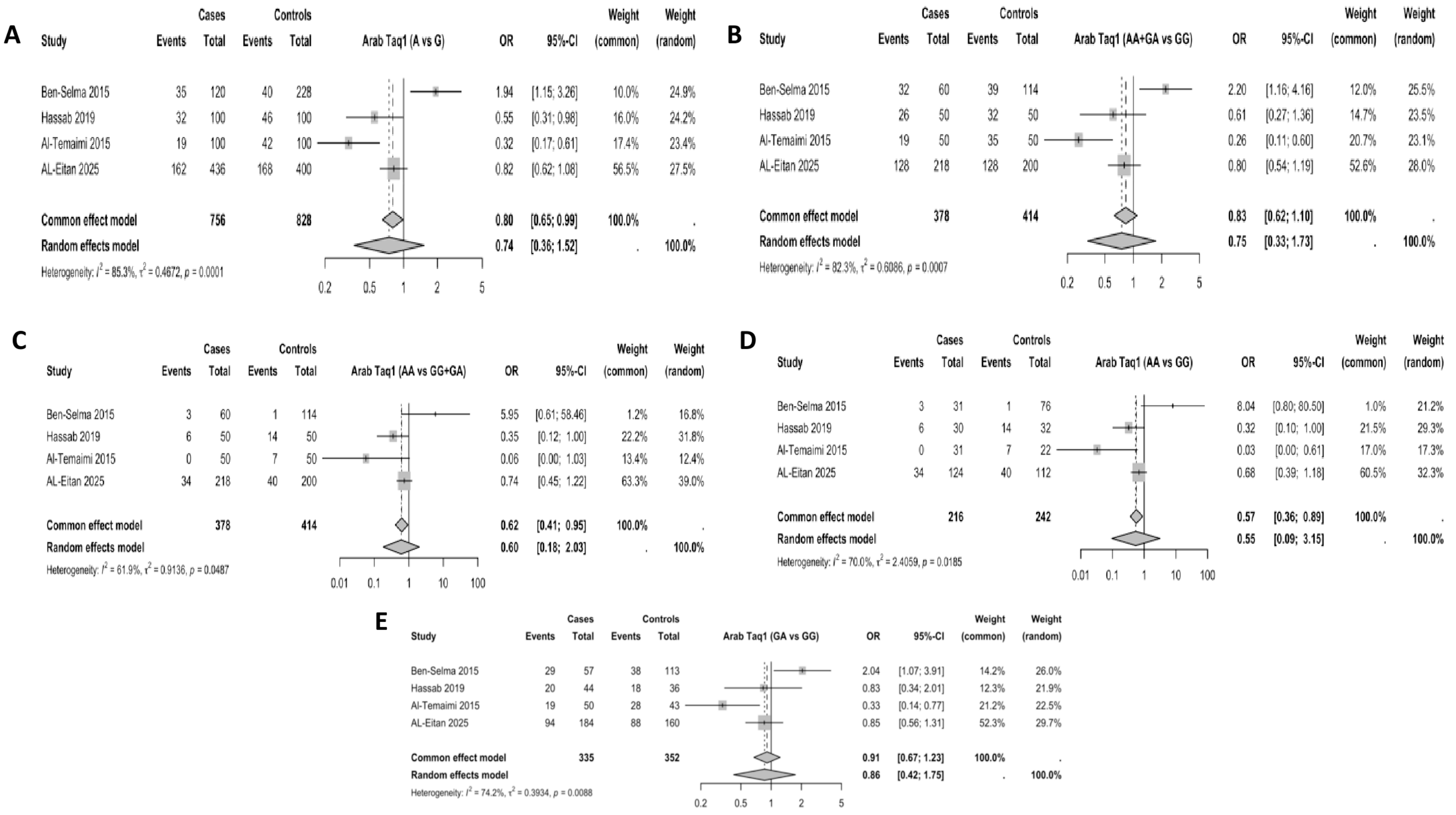

Figure S24. Association between the VDR Taq1 polymorphism and multiple sclerosis risk in Arab populations across genetic models. Forest plots summarizing the association between the vitamin D receptor (VDR) TaqI polymorphism and multiple sclerosis (MS) risk in Arab populations under different genetic models: **(A)** allelic (A vs G), **(B)** dominant (AA+GA vs GG), **(C)** recessive (AA vs GA+GG), **(D)** homozygous (AA vs GG), and **(E)** heterozygous (GA vs GG). Pooled odds ratios (ORs) and 95% confidence intervals (CIs) were estimated using common-effect and random-effects models. Between-study heterogeneity was assessed using Cochran's Q test and quantified with the I² statistic **[1,8-10]**.

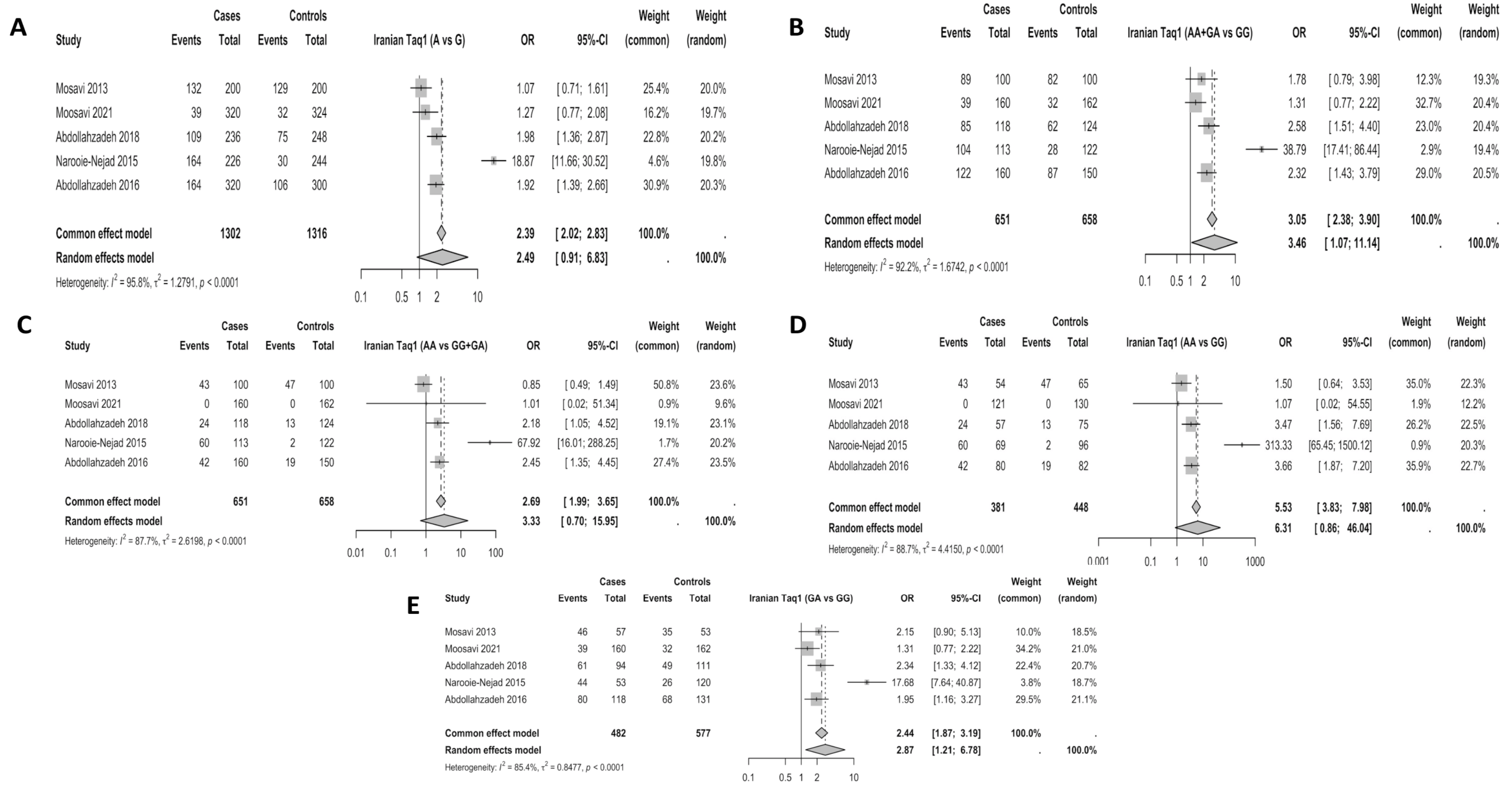

Figure S25. Association between the VDR Taq1 polymorphism and multiple sclerosis risk in Iranian populations across genetic models. Forest plots summarizing the association between the vitamin D receptor (VDR) TaqI polymorphism and multiple sclerosis (MS) risk in Iranian populations under different genetic models: **(A)** allelic (A vs G), **(B)** dominant (AA+GA vs GG), **(C)** recessive (AA vs GA+GG), **(D)** homozygous (AA vs GG), and **(E)** heterozygous (GA vs GG). Pooled odds ratios (ORs) and 95% confidence intervals (CIs) were estimated using common-effect and random-effects models. Between-study heterogeneity was assessed using Cochran's Q test and quantified with the I^2 statistic **[3,4,6,7,17]**.

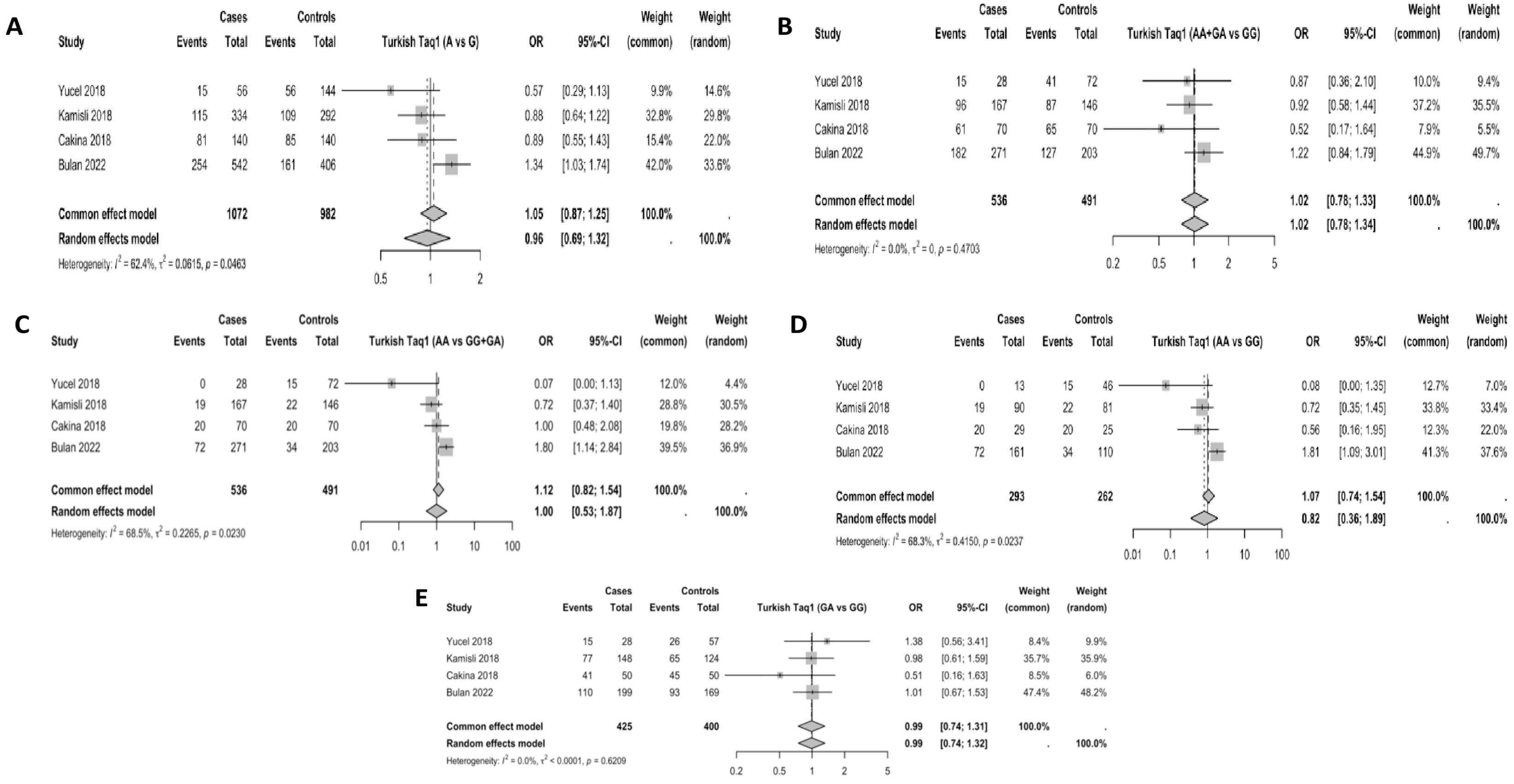

Figure S26. Association between the VDR Taq1 polymorphism and multiple sclerosis risk in Turkish populations across genetic models. Forest plots summarizing the association between the vitamin D receptor (VDR) TaqI polymorphism and multiple sclerosis (MS) risk in Turkish populations under different genetic models: **(A)** allelic (A vs G), **(B)** dominant (AA+GA vs GG), **(C)** recessive (AA vs GA+GG), **(D)** homozygous (AA vs GG), and **(E)** heterozygous (GA vs GG). Pooled odds ratios (ORs) and 95% confidence intervals (CIs) were estimated using common-effect and random-effects models. Between-study heterogeneity was assessed using Cochran's Q test and quantified with the I^2 statistic **[2,5,12,19]**.

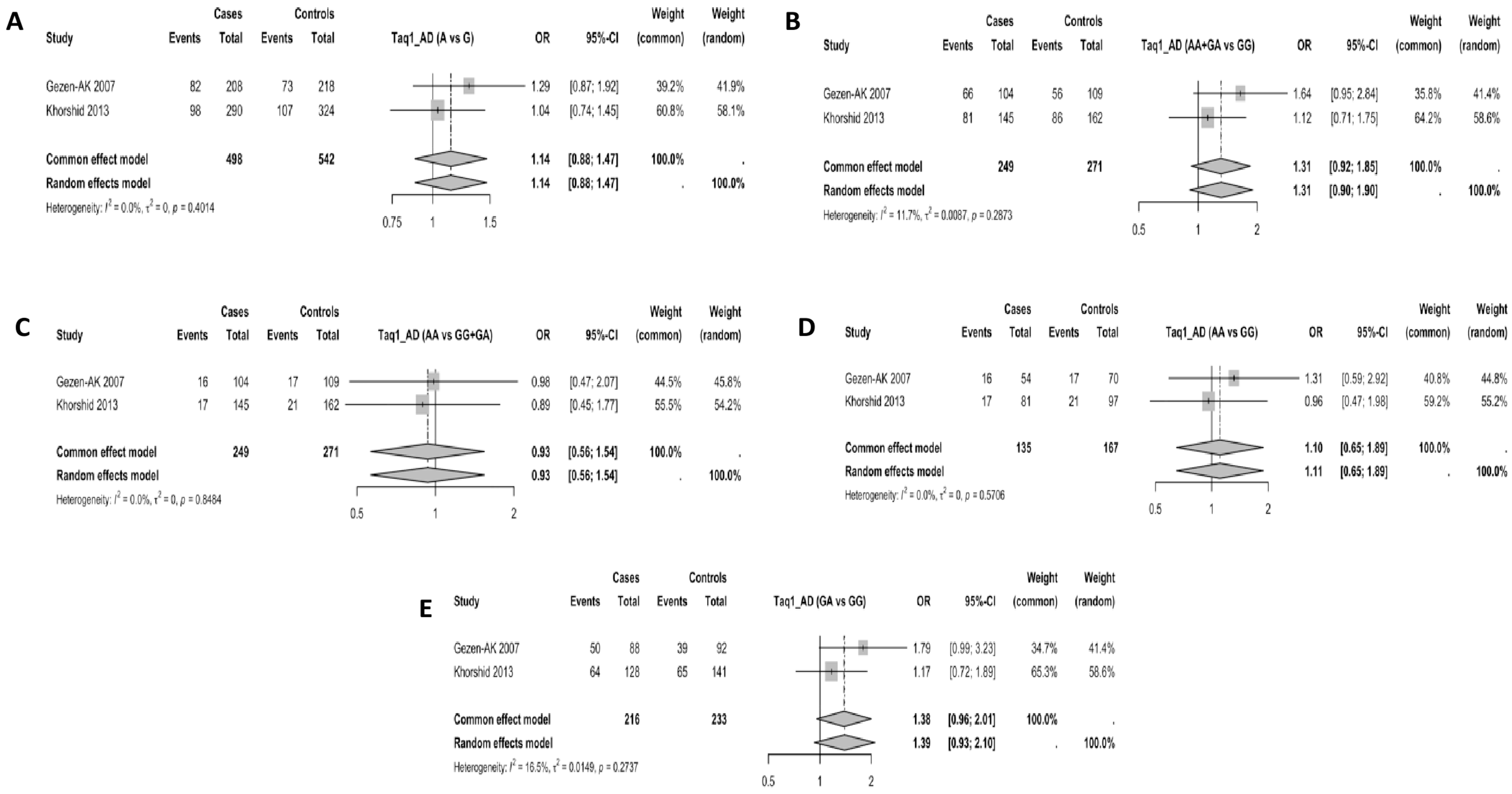

Figure S27. Association between the VDR Taq1 polymorphism and Alzheimer's disease risk across genetic models. Forest plots present pooled odds ratios (ORs) and 95% confidence intervals (CIs) for the association between the vitamin D receptor (VDR) Taq1 polymorphism and susceptibility to Alzheimer's disease (AD) across five genetic models: **(A)** allelic (A vs G), **(B)** dominant (AA+AG vs GG), **(C)** recessive (AA vs AG+GG), **(D)** homozygous (AA vs GG), and **(E)** heterozygous (AG vs GG). Individual study estimates are shown as squares proportional to study weight, with horizontal lines indicating 95% CIs. Diamonds represent pooled estimates derived under common-effect and random-effects models. Between-study heterogeneity was assessed using Cochran's Q test and quantified using the I^2 statistic **[13,14]**.

A

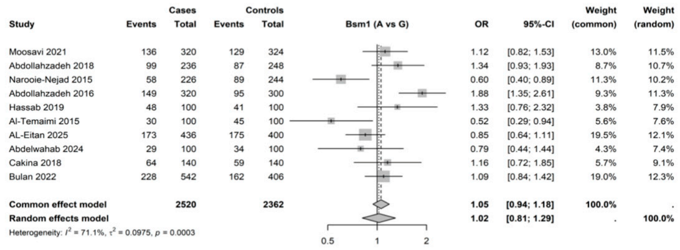

B

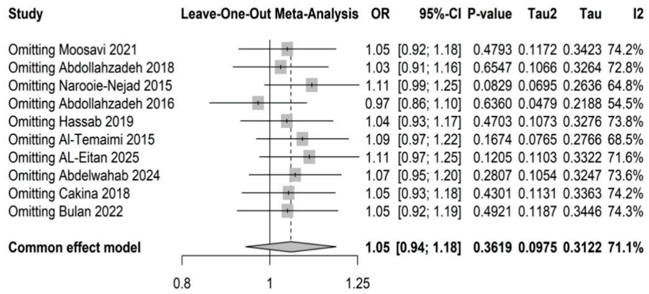

C

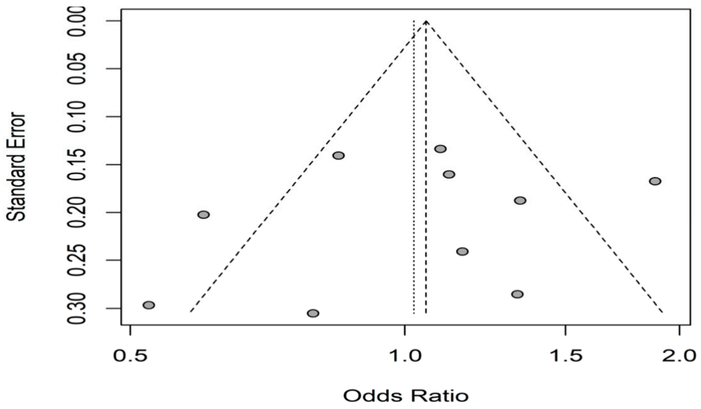

Figure S28. VDR Bsm1 allelic model analyses and multiple sclerosis risk in the overall population. Association analyses between the vitamin D receptor (VDR) BsmI polymorphism and disease risk under the allelic genetic model (A vs G) in the overall population are shown. **(A)** Forest plot of individual studies and pooled effect estimates. **(B)** Leave-one-out sensitivity analysis evaluating the influence of each study on the pooled estimate. **(C)** Funnel plot assessing small-study effects and potential publication bias. Pooled odds ratios (ORs) and 95% confidence intervals (CIs) were estimated using common-effect and random-effects models, with between-study heterogeneity assessed using Cochran's Q test and quantified by the I^2 statistic **[4,7-12,17,18,19]**.

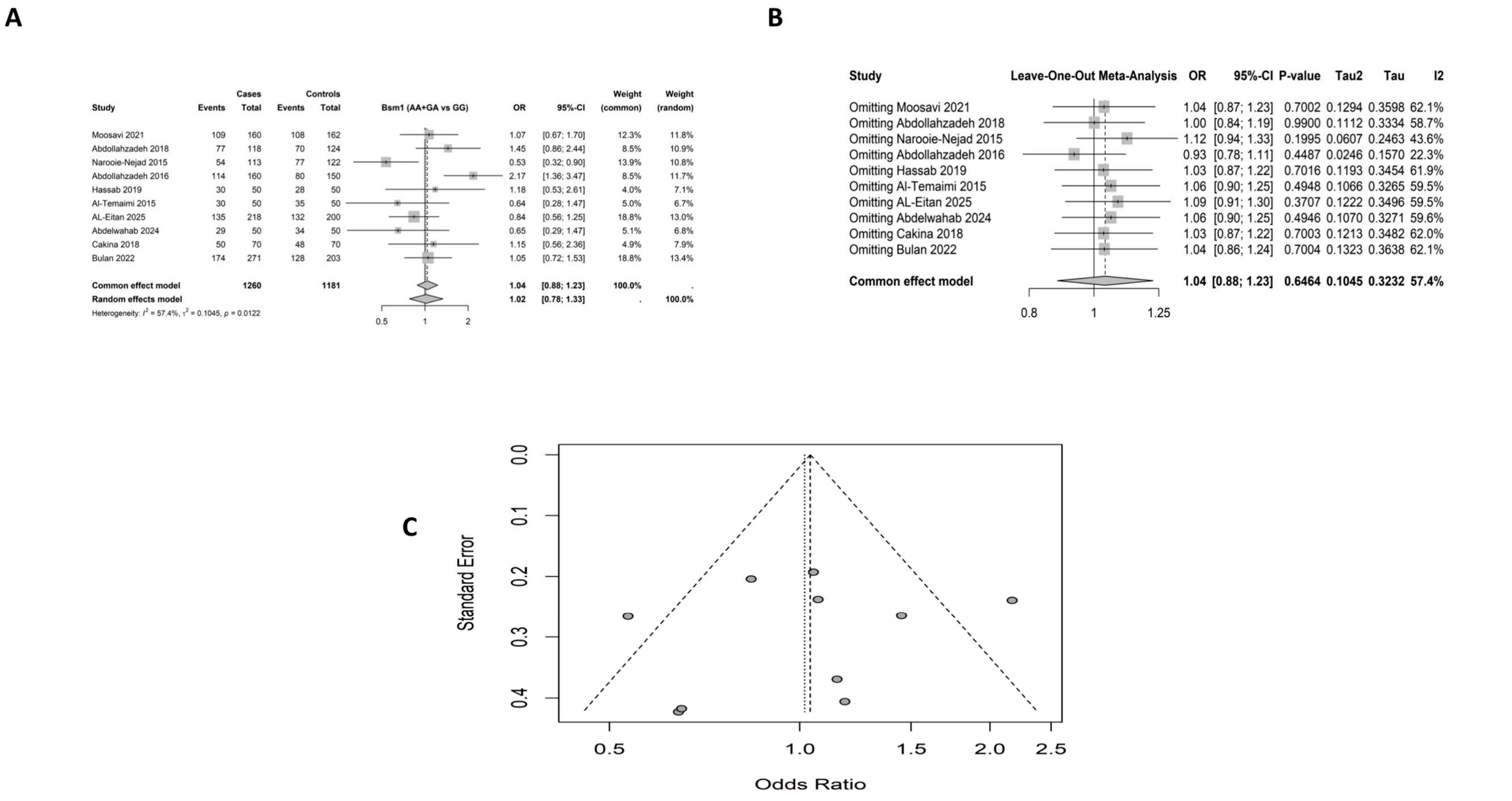

Figure S29. VDR Bsm1 dominant model analyses and multiple sclerosis risk in the overall population. Association analyses between the vitamin D receptor (VDR) BsmI polymorphism and disease risk under the dominant genetic model (AA + GA vs GG) in the overall population are shown. **(A)** Forest plot of individual studies and pooled effect estimates. **(B)** Leave-one-out sensitivity analysis evaluating the influence of each study on the pooled estimate. **(C)** Funnel plot assessing small-study effects and potential publication bias. Pooled odds ratios (ORs) and 95% confidence intervals (CIs) were estimated using common-effect and random-effects models, with between-study heterogeneity assessed using Cochran's Q test and quantified by the I^2 statistic **[4,7-12,17,18,19]**.

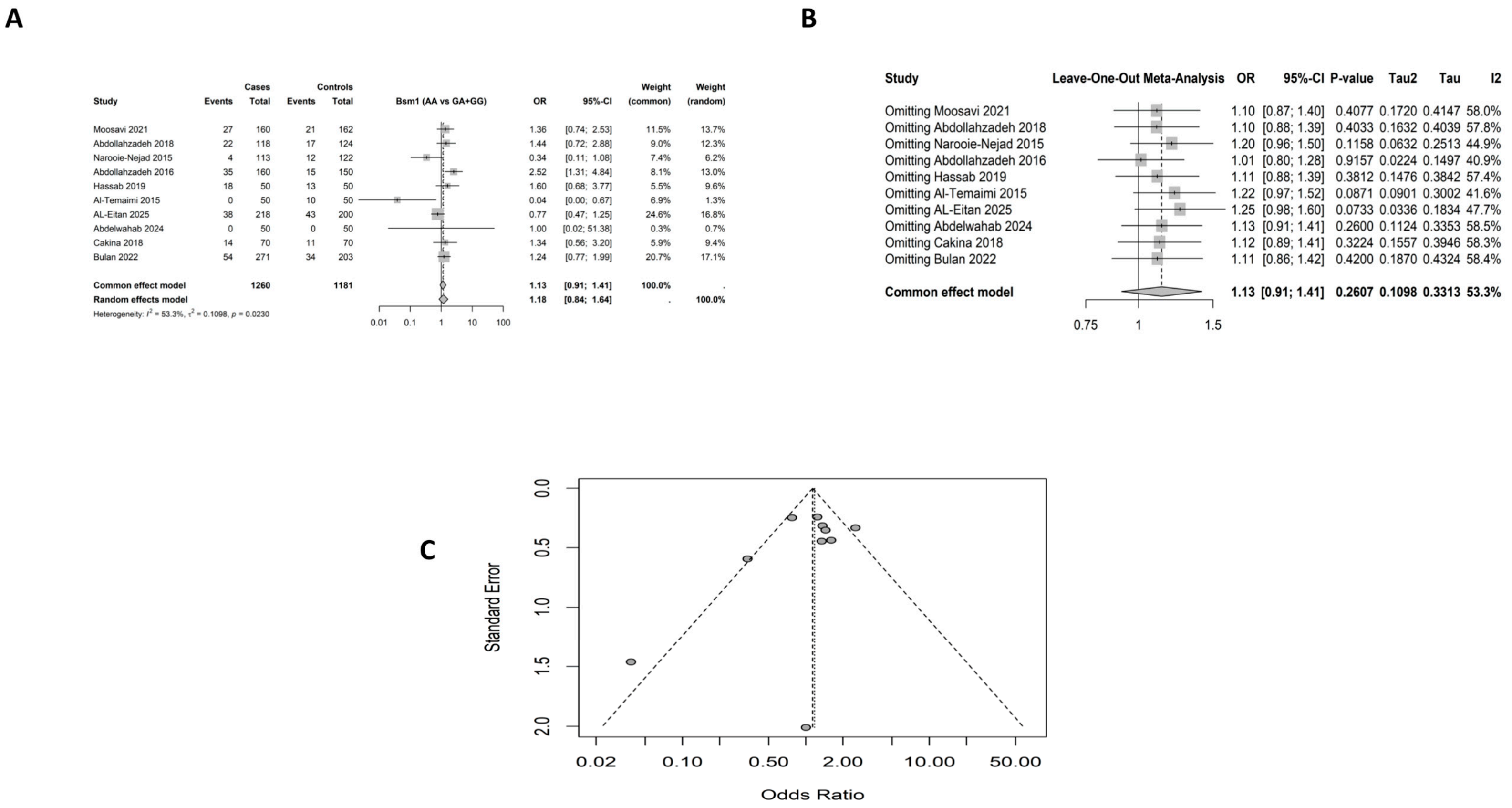

Figure S30. VDR Bsm1 recessive model analyses and multiple sclerosis risk in the overall population. Association analysis between the vitamin D receptor (VDR) BsmI polymorphism and disease risk under the recessive genetic model (AA vs GA + GG) in the overall population is shown. **(A)** Forest plot of individual studies and pooled effect estimates. **(B)** Leave-one-out sensitivity analysis evaluating the influence of each study on the pooled estimate. **(C)** Funnel plot assessing small-study effects and potential publication bias. Pooled odds ratios (ORs) and 95% confidence intervals (CIs) were estimated using common-effect and random-effects models, with between-study heterogeneity assessed using Cochran’s Q test and quantified by the I^2 statistic **[4,7-12,17,18,19]**.

A

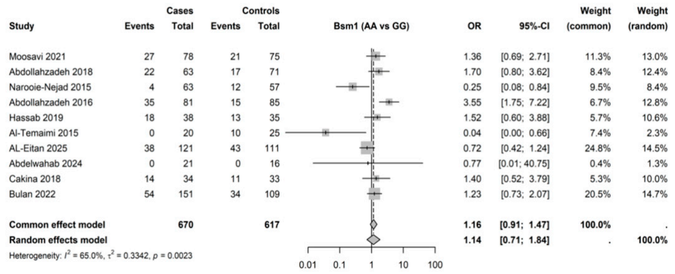

B

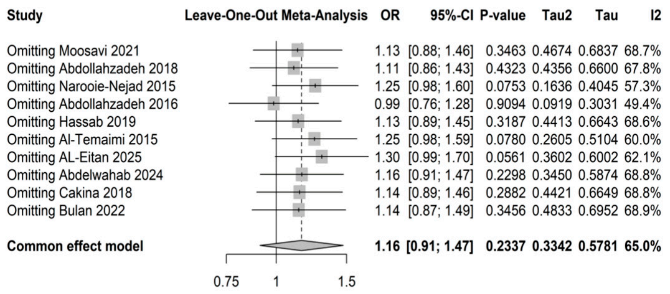

C

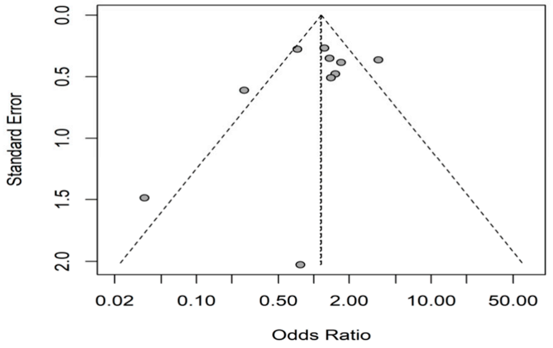

Figure S31. VDR Bsm1 homozygous model analyses and multiple sclerosis risk in the overall population. Association analysis between the vitamin D receptor (VDR) BsmI polymorphism and disease risk under the homozygous genetic model (AA vs GG) in the overall population are shown. **(A)** Forest plot of individual studies and pooled effect estimates. **(B)** Leave-one-out sensitivity analysis evaluating the influence of each study on the pooled estimate. **(C)** Funnel plot assessing small-study effects and potential publication bias. Pooled odds ratios (ORs) and 95% confidence intervals (CIs) were estimated using common-effect and random-effects models, with between-study heterogeneity assessed using Cochran's Q test and quantified by the I² statistic **[4,7-12,17,18,19]**.

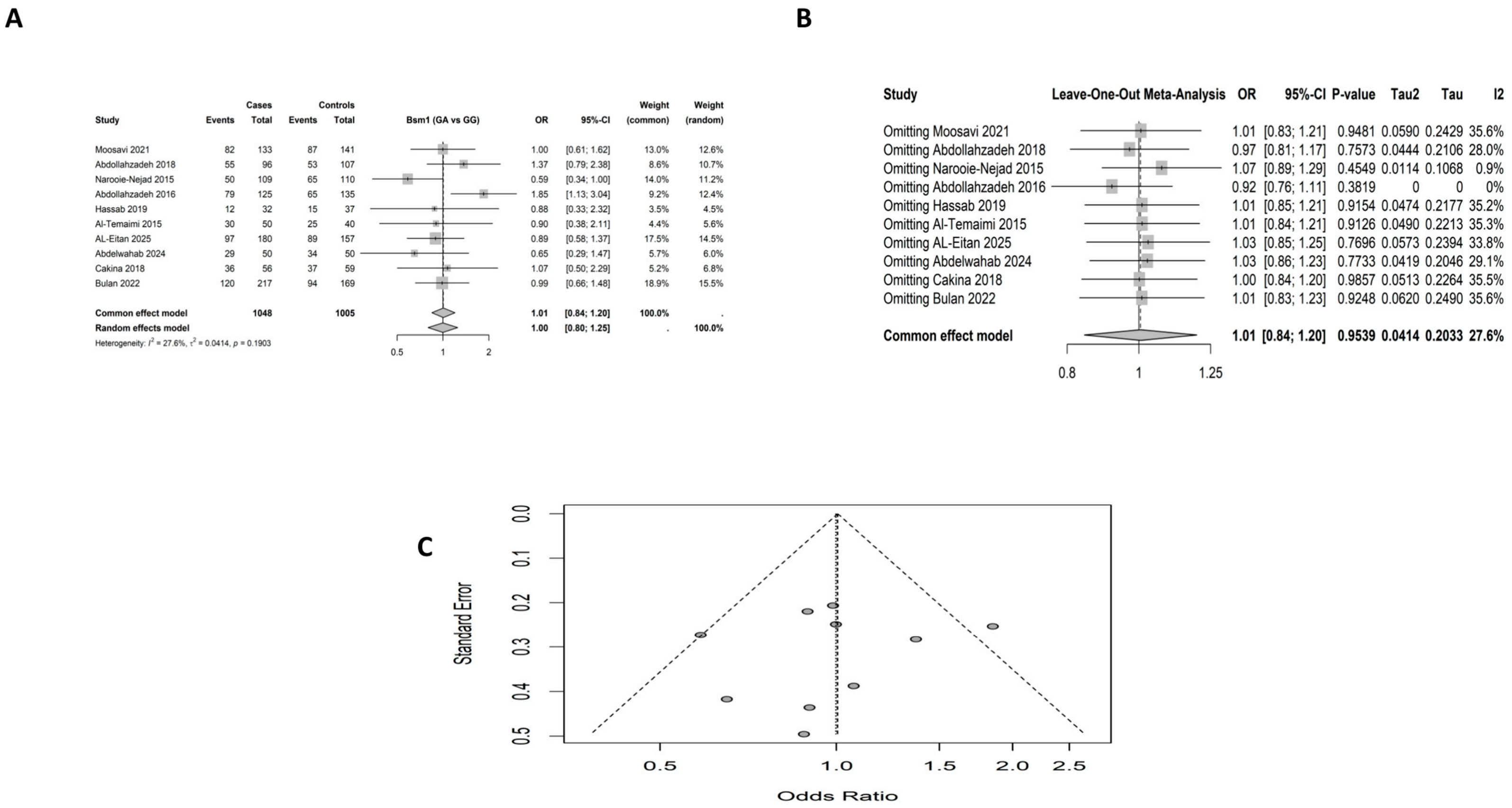

Figure S32. VDR Bsm1 heterozygous model analyses and multiple sclerosis risk in the overall population. Association analysis between the vitamin D receptor (VDR) BsmI polymorphism and disease risk under the heterozygous genetic model (GA vs GG) in the overall population are presented. **(A)** Forest plot showing individual study estimates and the pooled effect size. **(B)** Leave-one-out sensitivity analysis assessing the influence of each study on the overall estimate. **(C)** Funnel plot evaluating small-study effects and potential publication bias. Pooled odds ratios (ORs) and 95% confidence intervals (CIs) were calculated using common-effect and random-effects models, with between-study heterogeneity evaluated using Cochran’s Q test and quantified by the I^2 statistic **[4,7-12,17,18,19]**.

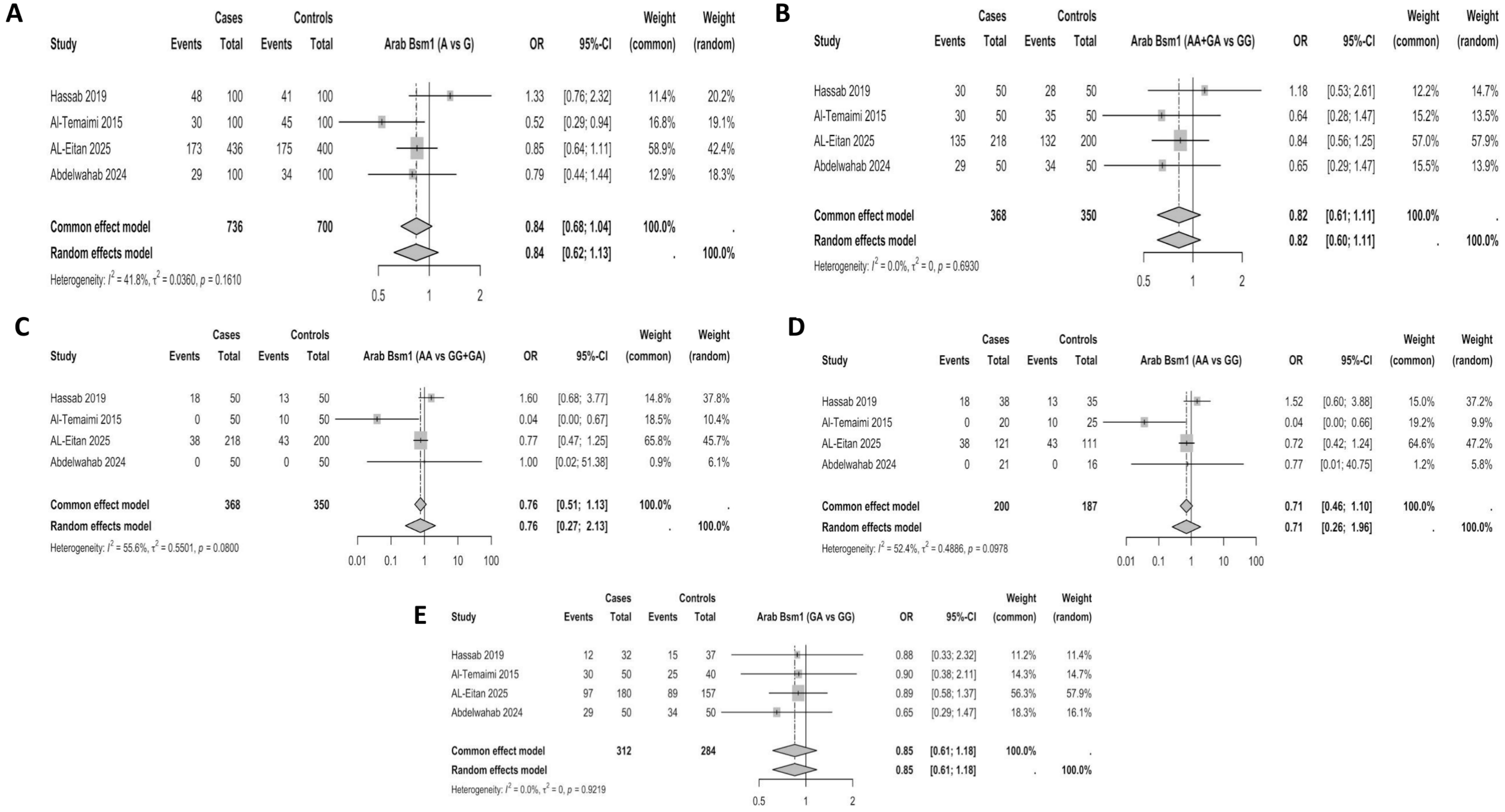

Figure S33. Association between the VDR Bsm1 polymorphism and multiple sclerosis risk in Arab populations across genetic models. Forest plots summarizing the association between the vitamin D receptor (VDR) Bsm1 polymorphism and multiple sclerosis (MS) risk in Arab populations under different genetic models: **(A)** allelic (A vs G), **(B)** dominant (AA+GA vs GG), **(C)** recessive (AA vs GA+GG), **(D)** homozygous (AA vs GG), and **(E)** heterozygous (GA vs GG). Pooled odds ratios (ORs) and 95% confidence intervals (CIs) were estimated using common-effect and random-effects models. Between-study heterogeneity was assessed using Cochran's Q test and quantified with the I^2 statistic **[8-11]**.

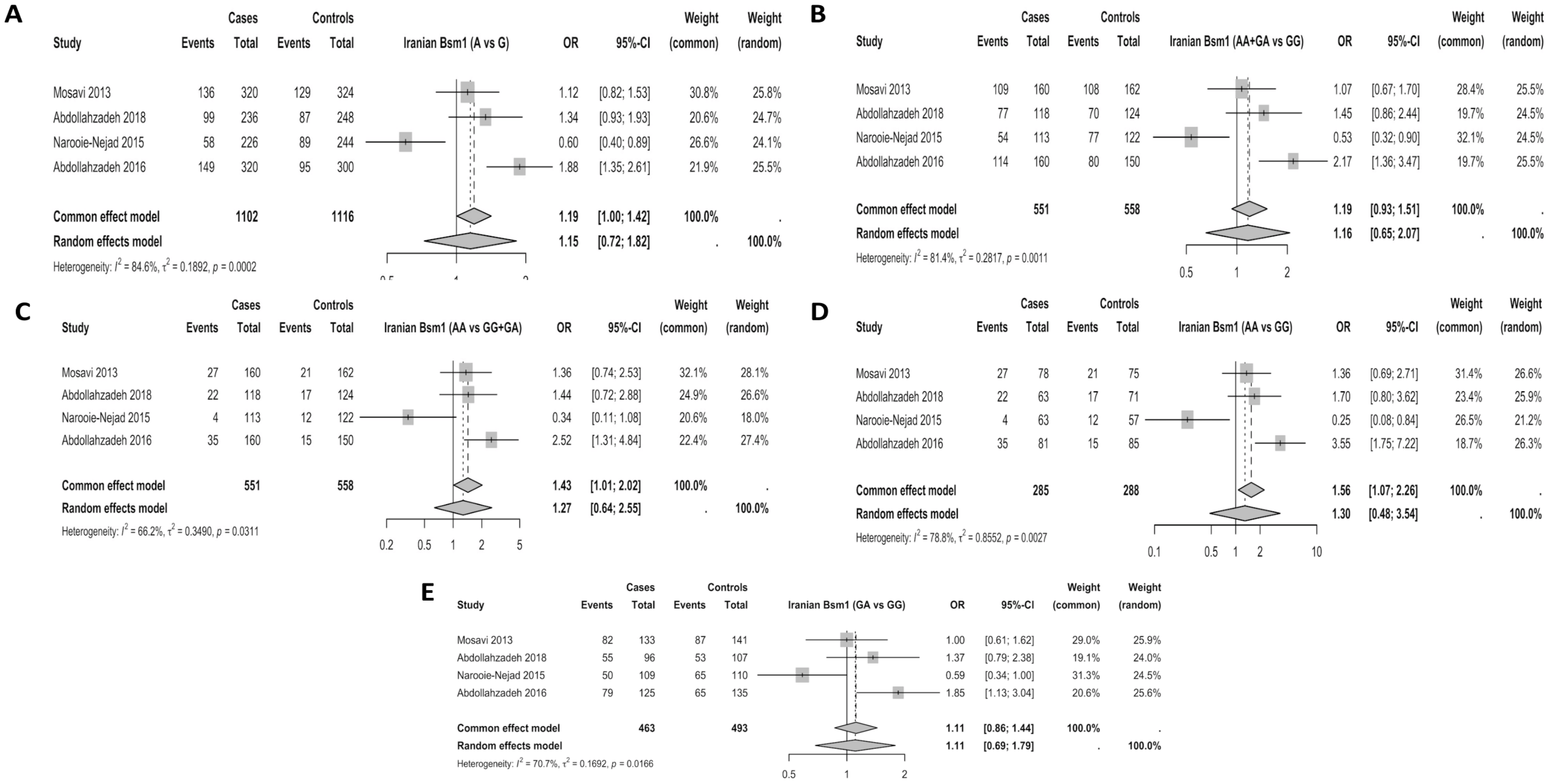

Figure S34. Association between the VDR Bsm1 polymorphism and multiple sclerosis risk in Iranian populations across genetic models. Forest plots summarizing the association between the vitamin D receptor (VDR) Bsm1 polymorphism and multiple sclerosis (MS) risk in Iranian populations under different genetic models: **(A)** allelic (A vs G), **(B)** dominant (AA+GA vs GG), **(C)** recessive (AA vs GA+GG), **(D)** homozygous (AA vs GG), and **(E)** heterozygous (GA vs GG). Pooled odds ratios (ORs) and 95% confidence intervals (CIs) were estimated using common-effect and random-effects models. Between-study heterogeneity was assessed using Cochran's Q test and quantified with the I² statistic **[3,4,7,18]**.

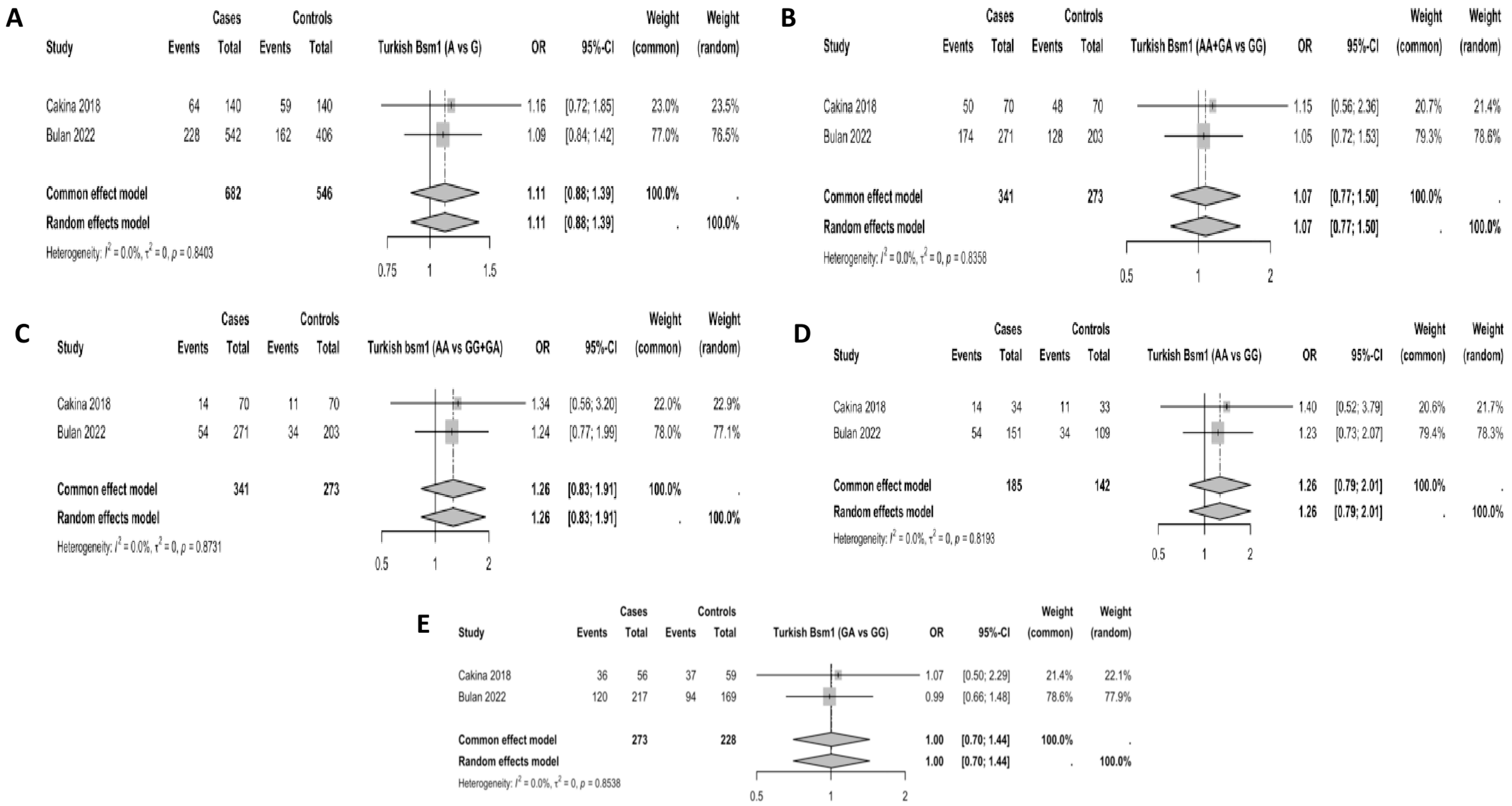

Figure S35. Association between the VDR Bsm1 polymorphism and multiple sclerosis risk in Turkish populations across genetic models. Forest plots summarizing the association between the vitamin D receptor (VDR) Bsm1 polymorphism and multiple sclerosis (MS) risk in Turkish populations under different genetic models: **(A)** allelic (A vs G), **(B)** dominant (AA+GA vs GG), **(C)** recessive (AA vs GA+GG), **(D)** homozygous (AA vs GG), and **(E)** heterozygous (GA vs GG). Pooled odds ratios (ORs) and 95% confidence intervals (CIs) were estimated using common-effect and random-effects models. Between-study heterogeneity was assessed using Cochran's Q test and quantified with the I^2 statistic **[12,19]**.

References

1. Ben-Selma, W.; Ben-Fredj, N.; Chebel, S.; Frih-Ayed, M.; Aouni, M.; Boukadida, J. Age- and gender-specific effects on VDR gene polymorphisms and risk of the development of multiple sclerosis in Tunisians: a preliminary study. *Int J Immunogenet* **2015**, *42*, 174-181, doi:10.1111/iji.12197.
2. Yucel, F.E.; Kamıslı, O.; Acar, C.; Sozen, M.; Tecellioğlu, M.; Ozcan, C. Analysis of Vitamin D Receptor Polymorphisms in Patients with Familial Multiple Sclerosis. *Med Arch* **2018**, *72*, 58-61, doi:10.5455/medarh.2017.72.58-61.
3. Mosavi, R.; Arababadi, M.K.; Hassanshahi, G.; Azin, H.; Araste, M.; Salehabad, V.A.; Vazirinejad, R.; Pourali, R.; Hakimi, H.; Kennedy, D. Association of a polymorphism within intron 8 but not exon 9 of the vitamin D receptor in patients with multiple sclerosis of southeastern iranian ethnicity. *Laboratory Medicine* **2013**, *44*, 215-219.
4. Abdollahzadeh, R.; Moradi Pordanjani, P.; Rahmani, F.; Mashayekhi, F.; Azarnezhad, A.; Mansoori, Y. Association of VDR gene polymorphisms with risk of relapsing-remitting multiple sclerosis in an Iranian Kurdish population. *Int J Neurosci* **2018**, *128*, 505-511, doi:10.1080/00207454.2017.1398158.
5. Kamisli, O.; Acar, C.; Sozen, M.; Tecellioglu, M.; Yücel, F.E.; Vaizoglu, D.; Özcan, C. The association between vitamin D receptor polymorphisms and multiple sclerosis in a Turkish population. *Mult Scler Relat Disord* **2018**, *20*, 78-81, doi:10.1016/j.msard.2018.01.002.
6. Narooie-Nejad, M.; Moossavi, M.; Torkamanzehi, A.; Moghtaderi, A.; Salimi, S. Vitamin D Receptor Gene Polymorphism and the Risk of Multiple Sclerosis in South Eastern of Iran. *J Mol Neurosci* **2015**, *56*, 572-576, doi:10.1007/s12031-015-0513-x.
7. Abdollahzadeh, R.; Fard, M.S.; Rahmani, F.; Moloudi, K.; Kalani, B.S.; Azarnezhad, A. Predisposing role of vitamin D receptor (VDR) polymorphisms in the development of multiple sclerosis: A case-control study. *J Neurol Sci* **2016**, *367*, 148-151, doi:10.1016/j.jns.2016.05.053.
8. Hassab, A.H.; Deif, A.H.; Elneely, D.A.; Tawadros, I.M.; Fayad, A.I. Protective association of VDR gene polymorphisms and haplotypes with multiple sclerosis patients in Egyptian population. *Egyptian Journal of Medical Human Genetics* **2019**, *20*, 4.
9. Al-Temaimi, R.A.; Al-Enezi, A.; Al-Serri, A.; Alroughani, R.; Al-Mulla, F. The Association of Vitamin D Receptor Polymorphisms with Multiple Sclerosis in a Case-Control Study from Kuwait. *PLoS One* **2015**, *10*, e0142265, doi:10.1371/journal.pone.0142265.
10. Al-Eitan, L.; Darabseh, S. VDR gene variants FokI and ApaI: Factors associated with susceptibility to multiple sclerosis. *PLoS One* **2025**, *20*, e0332473, doi:10.1371/journal.pone.0332473.
11. Abdelwahab, N.R.; Mabrouk, R.R.; Zakaria, N.M.; Abdel Nasser, A.; Mostafa, A.A.; Wahba, N.S. Vitamin D receptor gene polymorphism in Egyptian multiple sclerosis patients. *Egypt J Immunol* **2024**, *31*, 44-54.

12. Cakina, S.; Ocak, O.; Ozkan, A.; Yucel, S.; Karaman, H.I.O. Vitamin D receptor gene polymorphisms in multiple sclerosis disease: A case-control study. *Revista Romana de Medicina de Laborator* **2018**, *26*, 489-495.
13. Gezen-Ak, D.; Dursun, E.; Ertan, T.; Hanağasi, H.; Gürvit, H.; Emre, M.; Eker, E.; Oztürk, M.; Engin, F.; Yilmazer, S. Association between vitamin D receptor gene polymorphism and Alzheimer's disease. *Tohoku J Exp Med* **2007**, *212*, 275-282, doi:10.1620/tjem.212.275.
14. Khorram Khorshid, H.R.; Gozalpour, E.; Saliminejad, K.; Karimloo, M.; Ohadi, M.; Kamali, K. Vitamin D Receptor (VDR) Polymorphisms and Late-Onset Alzheimer's Disease: An Association Study. *Iran J Public Health* **2013**, *42*, 1253-1258.
15. Fahmy, E.M.; Elawady, M.E.; Sharaf, S.; Heneidy, S.; Ismail, R.S. Vitamin D receptor gene polymorphisms and idiopathic Parkinson disease: an Egyptian study. *The Egyptian Journal of Neurology, Psychiatry and Neurosurgery* **2021**, *57*, 102.
16. Gezen-Ak, D.; Alaylıoğlu, M.; Genç, G.; Gündüz, A.; Candaş, E.; Bilgiç, B.; Atasoy İ, L.; Apaydın, H.; Kızıltan, G.; Gürvit, H.; et al. GC and VDR SNPs and Vitamin D Levels in Parkinson's Disease: The Relevance to Clinical Features. *Neuromolecular Med* **2017**, *19*, 24-40, doi:10.1007/s12017-016-8415-9.
17. Moosavi, E.; Rafiei, A.; Yazdani, Y.; Eslami, M.; Saeedi, M. Association of serum levels and receptor genes BsmI, TaqI and FokI polymorphisms of vitamin D with the severity of multiple sclerosis. *J Clin Neurosci* **2021**, *84*, 75-81, doi:10.1016/j.jocn.2020.12.008.
18. Narooie-Nejad, M.; Moossavi, M.; Torkamanzehi, A.; Moghtaderi, A. Positive association of vitamin D receptor gene variations with multiple sclerosis in South East Iranian population. *Biomed Res Int* **2015**, *2015*, 427519, doi:10.1155/2015/427519.
19. Bulan, B.; Hoscan, A.Y.; Keskin, S.N.; Cavus, A.; Culcu, E.A.; Isik, N.; List, E.O.; Arman, A. Vitamin D Receptor Polymorphisms Among the Turkish Population are Associated with Multiple Sclerosis. *Balkan J Med Genet* **2022**, *25*, 41-50, doi:10.2478/bjmg-2022-0003.
